# Cost-effectiveness of different monitoring strategies in a screening and treatment programme for hepatitis B in The Gambia

**DOI:** 10.7189/jogh.13.04004

**Published:** 2023-01-20

**Authors:** Nora Schmit, Shevanthi Nayagam, Maud Lemoine, Gibril Ndow, Yusuke Shimakawa, Mark R Thursz, Timothy B Hallett

**Affiliations:** 1 MRC Centre for Global Infectious Disease Analysis, Department of Infectious Disease Epidemiology, Faculty of Medicine, Imperial College London, London, UK; 2 Division of Digestive Diseases, Department of Metabolism, Digestion and Reproduction, Imperial College London, London, UK; 3 Medical Research Council Unit The Gambia at London School of Hygiene and Tropical Medicine, Fajara, The Gambia; 4 Unité d’Épidémiologie des Maladies Émergentes, Institut Pasteur, Paris, France

## Abstract

**Background:**

Clinical management of chronic hepatitis B virus (HBV) infection is complex and access to antiviral treatment remains limited in sub-Saharan Africa. International guidelines recommend monitoring at least annually for disease progression among HBV-infected people not meeting treatment criteria at initial diagnosis. This study aimed to assess the impact and cost-effectiveness of alternative strategies for monitoring.

**Methods:**

We used a mathematical model of HBV transmission and natural history, calibrated to all available West African data, to project the population-level health impact, costs and cost-effectiveness of different monitoring strategies for HBV-infected individuals not initially eligible for antiviral treatment. We assumed that these patients were found in the year 2020 in a hypothetical community-based screening programme in The Gambia. Monitoring frequencies were varied between every 5 and every 1 year and targeted different age groups.

**Results:**

The currently recommended annual monitoring frequency was likely to be not cost-effective in comparison with other strategies in this setting. 5-yearly monitoring in 15-45-year olds, at US$338 per disability-adjusted life year averted, had the highest probability of being the most effective cost-effective monitoring strategy.

**Conclusions:**

Monitoring less frequently than once a year is a cost-effective strategy in a community-based HBV screening and treatment programme in The Gambia, with the optimal strategy depending on the cost-effectiveness threshold. Efficiencies may be gained by prioritising the 15-45-year age group for more intensive monitoring.

An estimated 80 million people have chronic hepatitis B virus (HBV) infection and 250 000 people die from HBV-associated liver disease annually in sub-Saharan Africa [1,2], despite availability of a hepatitis B vaccine and highly effective antiviral treatment such as tenofovir disoproxil fumarate (TDF) to prevent disease progression.

In high-prevalence settings, the World Health Organization (WHO) recommends general population testing for hepatitis B and routine access to antiviral treatment for eligible adults [3]. However, scaling up access in resource-limited high-prevalence settings is challenging because of the need for large-scale active case finding to diagnose chronic infection, which often remains asymptomatic, and the complex clinical management [4-6]. HBV-infected individuals must be assessed for treatment eligibility, as immediate antiviral treatment is only indicated according to clinical criteria, and those not meeting these at initial assessment require regular monitoring for future disease progression [7,8]. Previous economic evaluations have suggested various approaches for expanding access to testing and treatment in sub-Saharan Africa [9-11], but the feasibility and cost-effectiveness of monitoring the chronic carriers ineligible for treatment at initial diagnosis remain uninvestigated. Current recommendations of at least annual monitoring are based on international liver association guidelines [8,12], but it is not known whether this is cost-effective in resource-limited settings. Lack of longitudinal data in African populations means that geographical differences in the epidemiology and natural history of hepatitis B are currently not considered [13].

Therefore, we hypothesised that annual monitoring of initially treatment-ineligible HBV-infected individuals would not be cost-effective in a case study of a population-based treatment programme in The Gambia. With this aim, we assembled an extensive epidemiological data set of the HBV epidemic in The Gambia in a mathematical model, and investigated the health impact and cost-effectiveness of monitoring treatment-ineligible chronic carriers at different frequencies and focusing on different age groups.

## METHODS

### Model development and data sources

Data sources for developing a mathematical model were assembled using a scoping review of the published literature on HBV epidemiology and natural history in sub-Saharan Africa. Screening and review of the 5972 articles identified in a Medline database search identified The Gambia as having the most high-quality data to serve as a case study for modelling HBV control strategies (Section 2A in the **Online Supplementary Document**).

**Figure 1:**
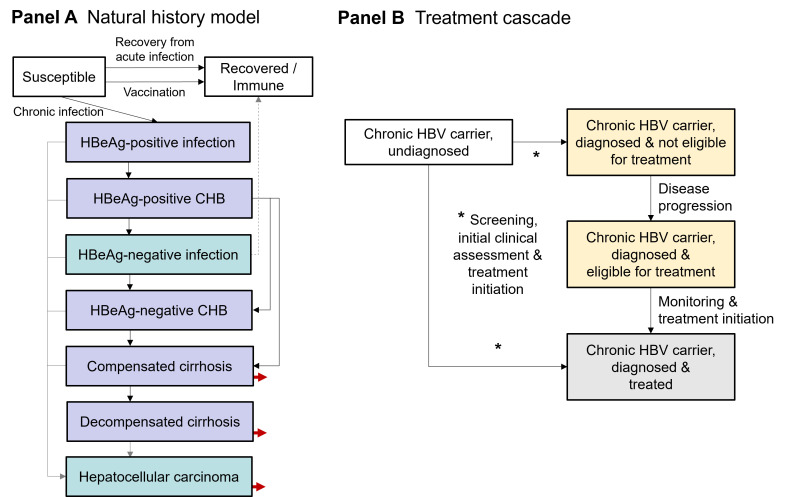
Model structure. **Panel A** shows an overview of the natural history model structure with the infection process, vaccination and further subdivisions into disease states in untreated chronic hepatitis B virus (HBV) carriers. The age, time- and sex-specific background mortality and net migration rates experienced in all compartments, as well as births into the susceptible and hepatitis B e antigen (HBeAg)-positive infection compartments, are not shown. Red arrows indicate additional mortality due to HBV-related liver disease. Purple compartments contain chronic HBV carriers eligible for antiviral treatment, whereas carriers in turquoise compartments do not meet treatment criteria. The dashed grey line represents serological recovery from chronic infection. **Panel B** shows the treatment cascade for chronic carriers. Model compartments are represented as boxes and arrows show annual transition rates between compartments, which can vary by age, sex and over time. Treatment initiation of undiagnosed carriers only applies to treatment-eligible disease states. Compartments in white contain the population to screen for hepatitis B surface antigen (HBsAg) and compartments in yellow contain the diagnosed population to monitor for disease progression to treatment eligibility. Carriers initiated on treatment remain on treatment for life or until serological recovery. CHB = chronic hepatitis B

Based on these data, we developed a dynamic deterministic model of hepatitis B transmission specific to a West African setting. The age- and sex-structured model projects the population of The Gambia from 1950 to 2100 according to their HBV infection, disease and treatment status. The model structure was adapted from published mathematical models [1,14] to reflect key epidemiological and clinical features of the West African HBV epidemic. HBV transmission occurs horizontally and vertically from mother to child at birth, accounting for the age-dependent risk of developing chronic infection and pre-vaccination HBV epidemiology in sub-Saharan Africa, where horizontal transmission in young children was the main source of new chronic infections [14,15]. The model includes historical coverage levels of infant vaccination against HBV since 1990 [16]. Chronic HBV infection was subdivided into seven compartments with different rates of developing liver disease according to clinical understanding of the natural history (**Figure 1**) [12]. Long-term chronic HBV infection can lead to hepatitis B-related mortality via development of liver cirrhosis or hepatocellular carcinoma (HCC).

In all treatment scenarios, chronic HBV carriers initiate indefinite antiviral therapy with the first-line nucleotide analogue TDF according to reference criteria for treatment eligibility developed by the European Association for the Study of the Liver (EASL) [12]. Treatment is assumed to stop disease progression to cirrhosis and reduces progression to HCC by 73%-81% depending on disease state at treatment initiation [17]. Patients are assumed to remain on antiviral therapy until death or until the rare outcome of serological recovery [12].

### Model calibration

We extracted data on HBV and liver disease burden in The Gambia and disease progression in West African HBV carriers from 38 studies included in the scoping review (Table S1 in the **Online Supplementary Document**). The model was calibrated to these data in a Bayesian framework by drawing 1 million parameter sets from prior distributions of all transmission and natural history parameters (Table S2.4 in the **Online Supplementary Document**). A rejection-sampling Approximate Bayesian Computation algorithm was used to obtain parameter sets producing the closest match between simulated trajectories and the available epidemiological data [18,19]. Forward projections under different intervention scenarios were made using 183 parameter sets accepted in the calibration to propagate uncertainty in input parameter values to model outcomes. Full details on modelling methods and calibration are provided in Section 2B and Section 2C in the **Online Supplementary Document**. All analyses were conducted in *R* statistical software version 4.0.3 [20].

### Intervention scenarios and model outcomes

We simulated different scenarios to investigate the potential impact and cost of monitoring initially treatment-ineligible HBV carriers in a hypothetical population-based screening and treatment programme for HBV in The Gambia. The base-case scenario assumed continuation of the current status quo of HBV control, corresponding to 3-dose infant vaccination at 93% coverage. We assumed no coverage of other interventions due to low estimates for timely birth dose vaccination within 24 hours and treatment coverage in The Gambia [2,21].

To this, we first compared the population health impact and cost of the screening and treatment programme without monitoring, and with annual monitoring potentially leading to subsequent treatment initiation. The programme was modelled as a mass screening and treatment campaign among adults in 2020, including serological testing for hepatitis B surface antigen (HBsAg) to identify HBV carriers, linkage to care for HBsAg-positive individuals to evaluate treatment eligibility, and initiation on antiviral therapy for treatment-eligible chronic carriers with annual monitoring for adherence and viral suppression [8,12]. Coverage levels at each stage of care were based on WHO service coverage targets for 2030 (**Table 1**) [22].

**Table 1:** Overview of scenarios for monitoring of carriers not eligible for antiviral treatment at initial assessment

Scenario*	Infant vaccination coverage (%)	Screening and treatment coverage	Monitoring frequency	Monitored age groups
Base-case	93	/	/	/
Screening and treatment without monitoring	93	90% screening for HBsAg, 80% clinical assessment among HBsAg-positive individuals, and 100% treatment initiation among identified treatment-eligible carriers.	/	/
Screening and treatment with recommended monitoring frequency	93	90% screening for HBsAg, 80% clinical assessment among HBsAg-positive individuals, 100% treatment initiation among identified treatment-eligible carriers, and 80% monitoring uptake among treatment-ineligible carriers at each visit	Every 1 year	All ages (15+)
Screening and treatment with alternative monitoring strategies	93	90% screening for HBsAg, 80% clinical assessment among HBsAg-positive individuals, 100% treatment initiation among identified treatment-eligible carriers, and 80% monitoring uptake among treatment-ineligible carriers at each visit	Every 5, 4, 3, 2 or 1 years	All ages (15+), 15-30, 15-45, 30+ or 45+ years

HBsAg – hepatitis B surface antigen

*Screening is assumed to take place in 2020 among 15-65 year olds and at the given coverage levels. Infant vaccination coverage is modelled at historical levels and continued at the most recent estimate of 93% in all scenarios.

Second, we compared the cost-effectiveness of these scenarios with alternative monitoring strategies less frequent than once a year. We created 25 monitoring scenarios using combinations of different average time intervals between monitoring assessments and targeting different age groups. We restricted monitoring frequencies to every 5, 4, 3, 2 and 1 year, as ensuring follow-up over longer intervals was considered infeasible. Since liver disease progression in chronic HBV carriers increases with age, we explored applying the same monitoring frequency across all ages, or offering monitoring only to 15-30, 15-45, over-30 or over-45-year olds.

The primary outcomes were cumulative HBV-related deaths and disability-adjusted life-years (DALYs) averted by the modelled treatment interventions over the 2020-2100 period compared to the base-case scenario. DALYs represent the years of life lost due to premature HBV-related death or disability associated with decompensated cirrhosis and HCC, calculated using disability weights from the Global Burden of Disease Study (Section 2D in the **Online Supplementary Document** [23].

### Cost-effectiveness analysis

Costs were estimated from a health care provider perspective in 2020 US dollars (US$). Costing data were collected in The Gambia or derived from a previous global study [10] and included active case finding using a rapid diagnostic test, diagnostic tests involved in clinical assessments, and antiviral treatment using TDF (**Table 2**, Section 2D in the **Online Supplementary Document**). As access to medical care for advanced liver disease is very limited in The Gambia, we made the conservative assumption that the treatment programme would not save costs associated with management of cirrhosis or HCC [6].

**Table 2:** Cost data in 2020 US$

Stage of care	Included resources*	Unit cost per person (US$)	Total cost per person (US$)	Range in one-way sensitivity analysis (US$)†	Source
Screening (one-time cost)	HBsAg rapid diagnostic test	1.70	8.30	4.00-17.00	[9,10]
	Programme cost	6.60			
Initial clinical assessment (one-time cost)	Viral load	13.00	33.00	16.50-200.00‡	Local data
	ALT	6.70			
	HBeAg	7.50			
	FibroScan	5.80			
Treatment (per year)	Tenofovir disoproxil fumarate	32.00	66.50	51.00-155.00§	[10], local data
	Monitoring: viral load	13.00			
	Monitoring: ALT	6.70			
	Monitoring: serum creatinine	6.70			
	Monitoring: urine dipstick	0.60			
	Monitoring: HBsAg	7.50			
Monitoring for treatment-ineligible carriers (per monitoring assessment	Viral load	13.00	25.50	12.75-51.00	Local data
	ALT	6.70			
	FibroScan	5.80			

ALT – alanine aminotransferase, HBeAg – hepatitis B e antigen, HBsAg – hepatitis B surface antigen

*Programme costs for community-based screening include human resources, consumables, transport, training and supervision. Laboratory costs include the associated cost of human resources, upfront purchase and maintenance of diagnostic devices.

†Cost estimates at each stage of care were halved or doubled in one-way sensitivity analyses unless otherwise indicated. Given that the initial and monitoring assessments largely include the same diagnostic tests, a joint increase in their costs by the same ratio was also explored.

‡Upper value based on [9].

§Range derived from minimum and maximum estimates for tenofovir disoproxil fumarate or monitoring based on [9,24].

Costs and health outcomes were discounted at 3% per year. The base-case and various screening and treatment strategies were compared by calculating incremental cost-effectiveness ratios (ICER) between non-dominated strategies as defined by

*ICER* = *(Cost _B_ – Cost _A_)* / *(DALYs averted _B_ – DALYs averted _A_)*

where strategy (B) is the next most effective strategy compared to (A).

We compared the ICERs to previously proposed cost-effectiveness thresholds based on benchmarks of health opportunity costs in The Gambia of 0.52 and 0.69 times the gross domestic product (GDP) per capita, corresponding to US$404 and US$537 per DALY averted, respectively [25]. The most effective cost-effective strategy was defined as the most effective scenario with a median ICER below US$404 per DALY averted. We calculated the probability of a given strategy being the most effective cost-effective across simulations for a range of thresholds of up to 3 times the GDP per capita [25].

### Sensitivity analysis

Uncertainty in transmission and natural history parameters are accounted for in calibration; results are reported as the median and 95% credible intervals (CrIs, 2.5^th^ and 97.5^th^ percentiles) of epidemic trajectories from the calibrated parameter sets. We calculated partial rank correlation coefficients to investigate how sensitive the estimated impact and ICERs were to parameters varied in the calibration [26]. We further varied fixed parameters within plausible ranges to investigate sensitivity of the model outcomes to parameters of treatment effect on disease progression, the coverage and costs at different stages of care, the infectiousness on treatment, discounting rates and a shorter time horizon.

## RESULTS

### Model fits

**Figure 2:**
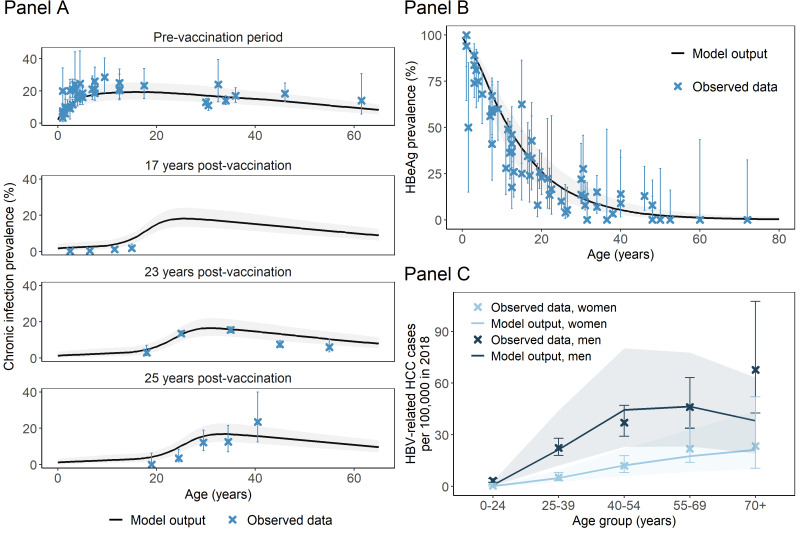
Comparisons between observed data and model outputs. **Panel A** shows the age-specific chronic hepatitis B virus (HBV) infection prevalence in The Gambia from various publications before 1990 (pre-vaccination), and from 17 [27], 23 [6] and 25 [28] years after introduction of the vaccine (2007, 2013 and 2015). **Panel B** shows the age-specific hepatitis B e antigen (HBeAg) prevalence in chronic HBV carriers derived from the sources in **Table 1****. Panel C** shows the age- and sex-specific HBV-related hepatocellular carcinoma (HCC) incidence rate in The Gambia in 2018 [29].

The model produced good fits to most published data sources used for calibration and reproduced the key patterns of HBV epidemiology and natural history in The Gambia by age, sex and over time, including chronic infection prevalence, hepatitis B e antigen (HBeAg) prevalence and liver disease outcomes (**Figure 2**, Section 2C in the **Online Supplementary Document**). Consistent with previous estimates [2,30], the all-age chronic HBV prevalence in 2020 was 5% (95% CrI = 3%-8%), after substantial declines in chronic infection incidence and HBV-related deaths due to infant vaccination (Figure S3.1 in the **Online Supplementary Document**).

### Population health impact of the screening and treatment programme without and with annual monitoring

A one-time population-wide screening and treatment intervention in 15-65-year olds without monitoring of those initially ineligible for antiviral treatment was estimated to avert 24% (95% CrI = 15%-34%) of 17 441 (95% CrI = 7394-31 003) HBV-related deaths and 25% (95% CrI = 14%-36%) of 433 134 (95% CrI = 167 916-854 370) DALYs projected to occur under the base-case scenario by 2100. Providing annual monitoring averted an additional 53% (95% CrI = 30%-75%) of the DALYs that would occur in the diagnosed cohort without monitoring, but 68% (95% CrI = 57%-81%) of these total averted DALYs resulted from the initial screening and treatment initiation rather than the subsequent monitoring.

**Figure 3:**
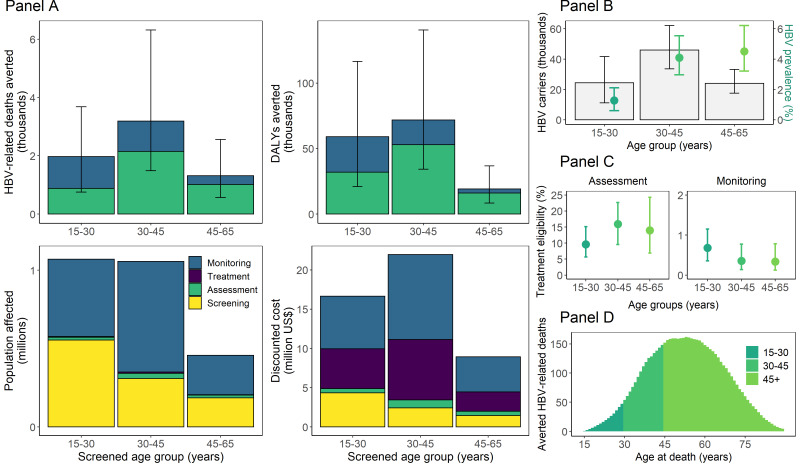
Population-level impact of the screening and treatment programme with annual monitoring. **Panel A** shows the epidemiological outcomes compared to the base-case scenario, and resource utilisation and costs involved with different stages of care over the 2020-2100 period by screened age group. Note that the population affected by monitoring represents the total number of monitoring assessments over time. **Panel B** explains the distribution of impact by age in relation to the distribution of chronic infection in the population at the time of screening. **Panel C** explains the distribution of impact by age in relation to the percentage of carriers eligible for treatment at initial and monitoring assessments. **Panel D** explains the distribution of impact by age in relation to the age distribution of HBV-related deaths averted by the treatment programme.

The population-level impact, resource utilisation and costs of screening, treating and monitoring different age groups in the current epidemic context of The Gambia is shown in **Figure 3**. The number of HBV-related deaths and DALYs averted by the treatment programme with annual monitoring was lowest among 45-65-year olds (**Figure 3****,** panel A). Despite a lower HBV prevalence in 15-30-year olds having benefited from routine vaccination (**Figure 3****,** panel B), the absolute number of DALYs averted was high among both 15-30 and 30-45-year olds due to the modelled observation that a larger proportion of monitoring assessments led to treatment initiation among 15-30-year olds (**Figure 3****,** panel C) and that 30% (95% CrI = 29%-47%) of HBV-related deaths averted by treatment were projected to occur before the age of 45 years (**Figure 3****,** panel D). Costs were also highest in under 45-year olds, and annual monitoring of those initially ineligible for treatment accounted for 46% of total costs (**Figure 3****,** panel A).

### Cost-effectiveness of alternative monitoring strategies

The one-time screening and treatment programme without monitoring averted an average of 76 703 DALYs at a cost of US$264 (95% CrI = 163-493) per DALY averted compared to the base-case (**Table 3**). Although this average value is below the cost-effectiveness threshold of US$404 per DALY averted, the programme without monitoring was dominated in 51% of simulations, where it had a higher ICER but smaller impact than a programme with monitoring. Compared to no monitoring, increasing monitoring frequencies were associated with an increase in overall health impact and cost, with a diminishing marginal impact on DALYs averted (**Table 3**, Figure S3.2 in the **Online Supplementary Document**). All age-specific monitoring strategies more frequent than every 4 years were dominated by age-specific strategies focusing less frequent monitoring on younger age groups, or by monitoring all groups. Monitoring 15-45-year-olds every 5 years was the most effective strategy with an ICER below the assumed cost-effectiveness threshold, at US$338 (95% CrI = 161-844) per DALY averted (**Table 3**). Compared to no treatment, this strategy averted 3258 (95% CrI = 1620-6110) HBV-related deaths and 88 916 (95% CrI = 41 776-162 768) DALYs.

**Table 3:** Costs and disability-adjusted life years averted between 2020 and 2100 for different treatment and monitoring strategies

Monitoring strategy	Cost (million US$)	DALYs averted vs. base-case (thousands)	Incremental costs (million US$)	Incremental DALYs averted (thousands)	ICER*
Base-case: no treatment	Reference				
No monitoring	20.60 (15.70-26.40)	76.70 (37.40-138.90)	/	/	Dominated (51%)
5-yearly in 15-30 years	21.60 (16.20-27.40)	79.40 (38.80-144.90)	21.60 (16.20-27.40)	79.40 (38.80-144.90)	263 (161-494)
4-yearly in 15-30 years	21.80 (16.30-27.60)	80.10 (39.00-145.70)	/	/	Dominated (92%)
3-yearly in 15-30 years	22.00 (16.50-27.90)	80.70 (39.20-146.70)	/	/	Dominated (98%)
2-yearly in 15-30 years	22.40 (16.70-28.60)	81.40 (39.40-148.10)	/	/	Dominated (100%)
Yearly in 15-30 years	23.50 (17.30-30.10)	82.30 (39.50-150.50)	/	/	Dominated (100%)
5-yearly in 15-45 years	24.40 (18.20-31.50)	88.90 (41.80-162.80)	2.90 (1.60-4.70)	8.50 (3.00-18.70)	338 (161-844)
4-yearly in 15-45 years	25.00 (18.50-32.40)	90.10 (42.10-165.20)	0.70 (0.30-1.00)	1.20 (0.40-2.80)	552 (231-1498)
5-yearly in 45+ years	25.20 (18.90-31.90)	87.10 (41.40-159.70)	/	/	Dominated (100%)
3-yearly in 15-45 years	26.00 (19.10-33.80)	91.80 (42.40-168.10)	/	/	Dominated (87%)
4-yearly in 45+ years	26.00 (19.60-32.90)	88.40 (41.70-161.60)	/	/	Dominated (100%)
3-yearly in 45+ years	27.30 (20.60-34.50)	89.30 (42.00-163.90)	/	/	Dominated (100%)
2-yearly in 15-45 years	27.60 (20.10-36.30)	94.00 (42.80-172.20)	/	/	Dominated (98%)
5-yearly in 30+ years	27.70 (20.70-35.60)	94.40 (43.60-174.10)	/	/	Dominated (93%)
5-yearly in all ages	28.30 (21.00-36.50)	96.60 (43.90-177.40)	3.30 (2.10-4.40)	5.80 (1.40-13.20)	591 (258-1818)
4-yearly in 30+ years	29.00 (21.60-37.20)	95.80 (44.10-177.00)	/	/	Dominated (99%)
2-yearly in 45+ years	29.60 (22.50-37.60)	90.50 (42.40-166.60)	/	/	Dominated (100%)
4-yearly in all ages	29.70 (22.00-38.30)	98.20 (44.40-180.50)	1.40 (0.80-2.00)	1.50 (0.50-3.90)	915 (354-2557)
3-yearly in 30+ years	30.90 (23.10-39.80)	97.80 (44.50-180.40)	/	/	Dominated (99%)
3-yearly in all ages	32.00 (23.50-41.10)	100.00 (44.80-184.10)	2.20 (1.30-3.20)	1.80 (0.60-4.60)	1237 (457-3655)
Yearly in 15-45 years	32.30 (23.30-42.80)	96.50 (43.20-177.00)	/	/	Dominated (99%)
2-yearly in 30+ years	34.60 (25.60-44.50)	100.10 (45.00-184.20)	/	/	Dominated (100%)
2-yearly in all ages	35.70 (26.30-46.80)	102.00 (45.40-188.00)	4.10 (2.60-6.00)	2.00 (0.60-5.40)	2060 (707-6407)
Yearly in 45+ years	36.70 (27.60-47.20)	91.90 (42.70-169.60)	/	/	Dominated (100%)
Yearly in 30+ years	45.00 (33.10-59.00)	102.80 (45.50-188.10)	/	/	Dominated (99%)
Yearly in all ages	47.50 (34.30-62.80)	104.00 (45.90-193.20)	11.60 (7.30-16.70)	2.10 (0.60-6.10)	5428 (1688-18 427)

DALY – disability-adjusted life year, ICER – incremental cost-effectiveness ratio

*Estimates of incremental cost, incremental DALYs averted, and incremental cost-effectiveness ratios are only calculated for non-dominated strategies. Strategies were considered to be dominated if they were dominated in over 50% of simulations, percentages are given in brackets. Strategies with median ICER below the cost-effectiveness threshold of US$404 were considered to be cost-effective.

**Figure 4:**
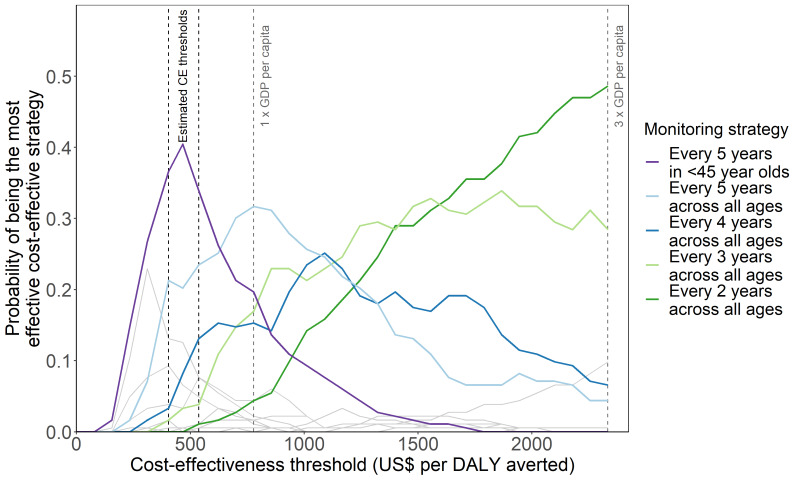
Cost-effectiveness acceptability curves for all monitoring strategies. Grey lines represent the monitoring strategies that never have the highest probability of being the most effective cost-effective strategy at any cost-effectiveness threshold within the evaluated range.

**Figure 4** shows the most effective cost-effective monitoring strategy depends on the assumed cost-effectiveness threshold. At a cost-effectiveness threshold of 1 times the GDP per capita, 5-yearly monitoring for all ages had the highest probability of being the most cost-effective strategy, compared to more frequent monitoring frequencies across all ages at cost-effectiveness thresholds higher than US$778 (**Figure 4**). Annual monitoring only had the highest probability of being the most effective cost-effective strategy at thresholds greater than US$5250 per DALY averted.

### Sensitivity analysis

Sensitivity analyses showed that projections of the impact and cost-effectiveness ratio of the treatment programme without monitoring, and of 5-yearly monitoring in <45-year-olds compared to no monitoring, were most sensitive to the progression rate from HBeAg-negative infection to HBeAg-negative chronic hepatitis B, representing progression to treatment eligibility among the largest subgroup of chronic HBV carriers (Table S3.1 in the **Online Supplementary Document**). This parameter also had the largest proportionate reduction in its uncertainty range after calibration, and the posterior median estimate, at 0.40% per year, was substantially lower than the prior value of 1.60% (Figure S2.11 in the **Online Supplementary Document**). Other influential parameters were those relating to progression to, decompensation or mortality from cirrhosis. Cost estimates at all stages of care, but particularly of treatment (including TDF and annual monitoring), also had among the greatest effect on the cost-effectiveness of the screening and treatment programme overall. Nevertheless, 5-yearly monitoring in 15-45-year-olds remained the most effective cost-effective strategy under most individual variations in the costs (Section 3C in the **Online Supplementary Document**).

## DISCUSSION

Our mathematical modelling study suggests that the currently recommended annual monitoring of initially treatment-ineligible HBV carriers is unlikely to be cost-effective in a population-based treatment programme in The Gambia. Assuming a cost-effectiveness threshold of US$404 per DALY averted, corresponding to 0.50 times the Gambian GDP per capita, we found that the most effective cost-effective strategy in this setting is monitoring every 5 years among 15-45-year olds. This would still substantially reduce the hepatitis B burden, averting an estimated 3258 HBV-related deaths and 88 916 DALYs between 2020 and 2100 compared to the base-case scenario of no screening and treatment.

To our knowledge this is the first study to investigate the population-level impact and cost-effectiveness of different monitoring frequencies for HBV treatment. A study on the cost-effectiveness of regular lifelong monitoring in treatment-ineligible HBV carriers in China found twice-yearly monitoring to be cost-effective compared to no monitoring [31], while an economic evaluation in a Gambian cohort also found a community-based screening and treatment programme with annual monitoring to be cost-effective compared to no treatment [9]. However, neither study compared the incremental effect and cost of twice-yearly or annual compared to less frequent or no monitoring, respectively. In contrast, our findings suggest that in resource-limited settings, less frequent monitoring could provide a better use of the available resources and minimise the burden of a large-scale treatment campaign on patients and the health system, compared to current international recommendations of annual monitoring [8,12].

The diminishing marginal returns with increasing monitoring frequencies contributed to our finding that applying annual monitoring in a population-based testing approach is unlikely to be cost-effective compared to less frequent monitoring even at higher cost-effectiveness thresholds of 3 times the GDP per capita. This was consistent across a wide range of model parameters and plausible ranges of treatment effect, coverage and costs. Nonetheless, although substantial uncertainty exists around the optimal monitoring strategy and ICERs are likely to vary across countries, our results also indicate that monitoring more frequently than every 5 years, including up to every 2 years, could be cost-effective in sub-Saharan African settings if higher cost-effectiveness thresholds are considered.

The modelled impact of different monitoring frequencies arose in part from the low progression rate from HBeAg-negative infection to HBeAg-negative chronic hepatitis B. Since there is no direct empirical data from Africa informing this parameter, it was estimated by calibrating the model to all relevant epidemiological data from West Africa. The calibration led to a modelled annual progression rate to treatment eligibility among untreated carriers of 0.60% (95% CrI = 0.30%-1.20%); similar to rates among European inactive carriers incidentally identified through blood donations [32], but much lower than the 2%-7% reported in hospital-based or Asian cohorts [33-37]. This highlights the need for more empirical data from African settings. Forthcoming results from follow-up of the PROLIFICA study, a population-based screen-and-treat programme started in The Gambia in 2011, should provide further empirical insights into disease progression among treatment-ineligible carriers [6,38]. The calibrated model also reproduced empirical observations of a younger average age of liver cancer patients in sub-Saharan Africa [39], which contributed to the finding that focusing the monitoring on younger as opposed to older age groups represents a more cost-effective strategy at lower estimates of the cost-effectiveness threshold.

Despite calibrating the model to all available published data, credible intervals around modelled outcomes were large, reflecting substantial uncertainty in natural history parameters of hepatitis B in sub-Saharan Africa. Estimation of absolute HBV-related mortality and treatment impact was particularly limited by a lack of empirical data on cirrhosis mortality, already identified as a data gap in previous studies [40].

The choice of treatment eligibility criteria could also have affected the impact of the initial assessment compared to monitoring, since our application of EASL guidelines assumed perfect sensitivity and specificity in identifying the respective disease states at a single assessment. The estimated proportion of treatment eligibility in chronic carriers of 14% (95% CrI = 9%-21%) was in line with ranges reported in African studies using the same criteria [7,41,42], but higher than the 4% (95% confidence interval (CI) = 3%-8%) in the community-based PROLIFICA study in The Gambia [6]. This difference could be due to adaptation of the EASL treatment criteria over time, different age distributions, or potential overestimation of treatment eligibility in women, as this was not stratified by sex in the model.

The long-term vaccination history in The Gambia raises considerations regarding the generalisability of our findings on the absolute population-level impact of screening and treatment to other sub-Saharan African countries, where the infant vaccine was often introduced much later and HBV prevalence in young adults likely remains higher [43]. Therefore, reductions in HBV-related deaths that can be achieved with one-time screening and treatment in other countries are likely to be more modest than those projected for The Gambia. The large population-level health gains in this study were also dependent on high levels of uptake of the intervention at all stages of care like those achieved in the PROLIFICA study [6]. In reality, this may be infeasible outside of a research context with active outreach, especially in countries with less diagnostic capacity [44]. Other practical considerations and barriers to implementation that were not addressed include the logistics involved with reaching rural populations, delivering clinical assessments, setting up reliable data linkage systems and minimising loss to follow-up with less frequent monitoring, which depend on local health care infrastructure and staff. Implementation could be facilitated through decentralisation of care and viral load testing, integration into existing health services, and novel simplified treatment criteria [4,5,11,45].

## CONCLUSIONS

Commitment for HBV elimination has increased globally and several African countries have planned or established national programmes for large-scale treatment [5]. Our case study of The Gambia supports improved access to HBV screening and treatment by showing that a simplified one-time programme with limited monitoring requirements is cost-effective and could significantly reduce HBV-related mortality in this setting, though further work is needed on the generalisability of this to other sub-Saharan African countries.

## Additional material


Online Supplementary Document
