# Supplementary material for: Cost-effectiveness of different monitoring strategies in a screening and treatment programme for hepatitis B in The Gambia
Source: J Glob Health. 2023 Jan 20;13:04004. doi: 10.7189/jogh.13.04004 (PMC9853089; doi:10.7189/jogh.13.04004)
Supplement: Online Supplementary Document [file jogh-13-04004-s001.pdf]

ONLINE SUPPLEMENTARY DOCUMENT

Cost-effectiveness of different monitoring strategies in a screening and treatment programme for hepatitis B in The Gambia

Nora Schmit[1*], Shevanthi Nayagam[1,2], Maud Lemoine[2], Gibril Ndow[2,3], Yusuke Shimakawa[4], Mark R. Thursz[2], Timothy B. Hallett[1].

*Corresponding author: n.schmit17@imperial.ac.uk

[1] MRC Centre for Global Infectious Disease Analysis, Department of Infectious Disease Epidemiology, Faculty of Medicine, Imperial College London, London, UK.

[2] Division of Digestive Diseases, Department of Metabolism, Digestion and Reproduction, Imperial College London, London, UK.

[3] Medical Research Council Unit The Gambia at London School of Hygiene and Tropical Medicine, Fajara, The Gambia.

[4] Unité d'Épidémiologie des Maladies Émergentes, Institut Pasteur, Paris, France.

Contents

1. Data sources 3

References for data sources 4

2. Supplementary methods 9

A. Data assembly 9

B. Model development.. 14

C. Model calibration 48

D. Additional assumptions in cost-effectiveness analysis 64

3. Supplementary results 66

A. Base case projections of the impact of infant vaccination.................................... 66

B. Cost-effectiveness of different monitoring strategies 67

C. Sensitivity analysis of cost-effectiveness results.. 68

D. Sensitivity analysis of the impact the treatment programme on reducing HBV incidence.. 73

4. References 74

1. Data sources

All data sources used for model parameterisation and calibration are summarised in **Table S1**, with references shown below.

Table S1. Overview of data sources for model priors and calibration.

Data category	Data	Locations	Sources
**Model inputs**			
**Transmission**	Mother-to-child transmission risk by maternal HBeAg status, relative infectiousness of HBeAg-positive vs. HBeAg-negative carriers	The Gambia, various sub-Saharan African countries	[1, 2]
**Disease progression in chronic HBV carriers**	Progression rate from HBeAg-negative infection to HBeAg-negative chronic hepatitis B, HBsAg loss rate	The Gambia, various West and Central African countries	[3, 4]
**Disease progression in liver disease patients**	Mortality rate from HCC and decompensated cirrhosis	Ivory Coast, Nigeria	[5, 6]
**Interventions**	Infant vaccine efficacy against chronic infection	The Gambia	[7-11]
**Calibration targets**			
**Seromarker prevalence**	HBsAg prevalence, anti-HBc prevalence, HBeAg prevalence in chronic carriers	The Gambia	[4, 7, 9, 12-29]
**Transmission**	Mother-to-child transmission risk, age-specific risk of chronic carriage, chronic infection incidence, force of infection, percentage of chronic infections attributable to mother-to-child transmission	The Gambia, Benin, Liberia, Nigeria, Senegal	[4, 7, 21, 26, 29-36]
**Disease progression in chronic HBV carriers**	HBsAg loss rate, HBeAg loss rate, HCC incidence rate, decompensated cirrhosis incidence rate, all-cause mortality rate	The Gambia, Senegal	[4, 36]
**Disease progression in liver disease patients**	Cumulative mortality/mortality rate, cumulative HCC incidence	The Gambia, Mali, Nigeria, various sub-Saharan African countries	[37-41]
**Cross-sectional characteristics of chronic HBV carriers**	Prevalence of different infection and disease states in chronic HBV carriers	The Gambia	[4, 16]
**Cross-sectional characteristics of liver disease patients**	Prevalence of HBeAg in cirrhosis and HCC patients, mean age and percentage of male sex among cirrhosis and HCC patients, percentage of deaths due to end-stage liver disease, prevalence of compensated and decompensated cirrhosis in HCC patients	The Gambia	[20, 22, 40, 42]

**Risk factors for liver disease in chronic HBV carriers**	Odds ratios for association of current HBeAg positivity and cirrhosis/HCC, odds ratio for association of male sex and significant liver fibrosis or cirrhosis	The Gambia	[4, 20]
**Modelled liver disease burden**	HCC incidence and mortality, cirrhosis mortality	The Gambia	[43-46]

Abbreviations: anti-HBc = hepatitis B core antibody, HBeAg = hepatitis B e antigen, HBsAg = hepatitis B surface antigen, HCC = hepatocellular carcinoma.

References for data sources

1. Keane E, Funk AL, Shimakawa Y. Systematic review with meta-analysis: the risk of mother-to-child transmission of hepatitis B virus infection in sub-Saharan Africa. Alimentary Pharmacology & Therapeutics **2016**; 44(10): 1005-17.

2. Shimakawa Y, Bottomley C, Njie R, Mendy M. The association between maternal hepatitis B e antigen status, as a proxy for perinatal transmission, and the risk of hepatitis B e antigenaemia in Gambian children. BMC Public Health **2014**; 14: 532.

3. Boglione L, Cusato J, Cariti G, Di Perri G, D'Avolio A. The E genotype of hepatitis B: clinical and virological characteristics, and response to interferon. J Infect **2014**; 69(1): 81-7.

4. Shimakawa Y, Lemoine M, Njai HF, Bottomley C, Ndow G, Goldin RD, Jatta A, Jeng-Barry A, Wegmuller R, Moore SE, Baldeh I, Taal M, D'Alessandro U, Whittle H, Njie R, Thursz M, Mendy M. Natural history of chronic HBV infection in West Africa: a longitudinal population-based study from The Gambia. Gut **2016**; 65(12): 2007-16.

5. Attia KA, Ackoundou-N'guessan K C, N'Dri-Yoman AT, Mahassadi AK, Messou E, Bathaix YF, Kissi YH. Child-Pugh-Turcott versus Meld score for predicting survival in a retrospective cohort of black African cirrhotic patients. World J Gastroenterol **2008**; 14(2): 286-91.

6. Olubuyide IO. The natural history of primary liver cell carcinoma: a study of 89 untreated adult Nigerians. Cent Afr J Med **1992**; 38(1): 25-30.

7. Fortuin M, Chotard J, Jack AD, Maine NP, Mendy M, Hall AJ, Inskip HM, George MO, Whittle HC. Efficacy of hepatitis B vaccine in the Gambian expanded programme on immunisation. The Lancet **1993**; 341(8853): 1129-31.

8. Mendy M, Peterson I, Hossin S, Peto T, Jobarteh ML, Jeng-Barry A, Sidibeh M, Jatta A, Moore SE, Hall AJ, Whittle H. Observational study of vaccine efficacy 24 years after the start of hepatitis B vaccination in two Gambian villages: no need for a booster dose. PLoS One **2013**; 8(3): e58029.

9. Peto TJ, Mendy ME, Lowe Y, Webb EL, Whittle HC, Hall AJ. Efficacy and effectiveness of infant vaccination against chronic hepatitis B in the Gambia Hepatitis Intervention Study (1986-90) and in the nationwide immunisation program. BMC Infect Dis **2014**; 14: 7.

10. van der Sande MA, Waight PA, Mendy M, Zaman S, Kaye S, Sam O, Kahn A, Jeffries D, Akum AA, Hall AJ, Bah E, McConkey SJ, Hainaut P, Whittle HC. Long-term protection against HBV chronic carriage of Gambian adolescents vaccinated in infancy and immune response in HBV booster trial in adolescence. PLoS One **2007**; 2(8): e753.

11. Viviani S, Jack A, Hall AJ, Maine N, Mendy M, Montesano R, Whittle HC. Hepatitis B vaccination in infancy in The Gambia: protection against carriage at 9 years of age. Vaccine **1999**; 17(23-24): 2946-50.

12. Bellamy R, Ruwende C, McAdam KP, Thursz M, Sumiya M, Summerfield J, Gilbert SC, Corrah T, Kwiatkowski D, Whittle HC, Hill AV. Mannose binding protein deficiency is not associated with malaria, hepatitis B carriage nor tuberculosis in Africans. Qjm **1998**; 91(1): 13-8.

13. Bittaye M, Idoko P, Ekele BA, Obed SA, Nyan O. Hepatitis B virus sero-prevalence amongst pregnant women in the Gambia. BMC Infect Dis **2019**; 19(1): 259.

14. Chotard J, Inskip HM, Hall AJ, Loik F, Mendy M, Whittle H, George MO, Lowe Y. The Gambia Hepatitis Intervention Study: follow-up of a cohort of children vaccinated against hepatitis B. J Infect Dis **1992**; 166(4): 764-8.

15. Kirk GD, Lesi OA, Mendy M, Akano AO, Sam O, Goedert JJ, Hainaut P, Hall AJ, Whittle H, Montesano R. The Gambia Liver Cancer Study: Infection with hepatitis B and C and the risk of hepatocellular carcinoma in West Africa. Hepatology **2004**; 39(1): 211-9.

16. Lemoine M, Shimakawa Y, Njie R, Taal M, Ndow G, Chemin I, Ghosh S, Njai HF, Jeng A, Sow A, Toure-Kane C, Mboup S, Suso P, Tamba S, Jatta A, Sarr L, Kambi A, Stanger W, Nayagam S, Howell J, Mpabanzi L, Nyan O, Corrah T, Whittle H, Taylor-Robinson SD, D'Alessandro U, Mendy M, Thursz MR, investigators P. Acceptability and feasibility of a screen-and-treat programme for hepatitis B virus infection in The Gambia: the Prevention of Liver Fibrosis and Cancer in Africa (PROLIFICA) study. Lancet Glob Health **2016**; 4(8): e559-67.

17. Vall Mayans M, Hall AJ, Inskip HM, Chotard J, Lindsay SW, Coromina E, Mendy M, Alonso PL, Whittle H. Risk factors for transmission of hepatitis B virus to Gambian children. Lancet **1990**; 336(8723): 1107-9.

18. Mendy ME, Fortuin M, Hall AJ, Jack AD, Whittle HC. Hepatitis B virus DNA in relation to duration of hepatitis B surface antigen carriage. Br J Biomed Sci **1999**; 56(1): 34-8.

19. Mendy ME, McConkey SJ, Sande van der MA, Crozier S, Kaye S, Jeffries D, Hall AJ, Whittle HC. Changes in viral load and HBsAg and HBeAg status with age in HBV chronic carriers in The Gambia. Virol J **2008**; 5: 49.

20. Mendy ME, Welzel T, Lesi OA, Hainaut P, Hall AJ, Kuniholm MH, McConkey S, Goedert JJ, Kaye S, Rowland-Jones S, Whittle H, Kirk GD. Hepatitis B viral load and risk for liver cirrhosis and hepatocellular carcinoma in The Gambia, West Africa. J Viral Hepat **2010**; 17(2): 115-22.

21. Ryder RW, Whittle HC, Wojiecowsky T, Moffat WM, Baker BA, Sarr E, Oldfield F. Screening for hepatitis B virus markers is not justified in West African transfusion centres. The Lancet **1984**; 2(8400): 449-52.

22. Ryder RW, Whittle HC, Sanneh AB, Ajdukiewicz AB, Tulloch S, Yvonnet B. Persistent hepatitis B virus infection and hepatoma in The Gambia, west Africa. A case-control study of 140 adults and their 603 family contacts. Am J Epidemiol **1992**; 136(9): 1122-31.

23. Thursz MR, Kwiatkowski D, Allsopp CE, Greenwood BM, Thomas HC, Hill AV. Association between an MHC class II allele and clearance of hepatitis B virus in the Gambia. N Engl J Med **1995**; 332(16): 1065-9.

24. van der Sande MA, Waight P, Mendy M, Rayco-Solon P, Hutt P, Fulford T, Doherty C, McConkey SJ, Jeffries D, Hall AJ, Whittle HC. Long-term protection against carriage of hepatitis B virus after infant vaccination. J Infect Dis **2006**; 193(11): 1528-35.

25. Whittle HC, Bradley AK, McLauchlan K, Ajdukiewicz AB, Howard CR, Zuckerman AJ, McGregor IA. Hepatitis B virus infection in two Gambian villages. The Lancet **1983**; 1(8335): 1203-6.

26. Whittle H, Inskip H, Bradley AK, McLaughlan K, Shenton F, Lamb W, Eccles J, Baker BA, Hall AJ. The pattern of childhood hepatitis B infection in two Gambian villages. J Infect Dis **1990**; 161(6): 1112-5.

27. Whittle HC, Inskip H, Hall AJ, Mendy M, Downes R, Hoare S. Vaccination against hepatitis B and protection against chronic viral carriage in The Gambia. The Lancet **1991**; 337(8744): 747-50.

28. Whittle HC, Maine N, Pilkington J, Mendy M, Fortuin M, Bunn J, Allison L, Howard C, Hall A. Long-term efficacy of continuing hepatitis B vaccination in infancy in two Gambian villages. The Lancet **1995**; 345(8957): 1089-92.

29. Wild CP, Fortuin M, Donato F, Whittle HC, Hall AJ, Wolf CR, Montesano R. Aflatoxin, liver enzymes, and hepatitis B virus infection in Gambian children. Cancer Epidemiology, Biomarkers and Prevention **1993**; 2(6): 555-61.

30. Ayoola EA, Ogunbode O, Odelola HA. Congenital transmission of hepatitis B antigen in Nigerians. Arch Virol **1981**; 67(1): 97-9.

31. Ayoola EA, Johnson AO. Hepatitis B vaccine in pregnancy: immunogenicity, safety and transfer of antibodies to infants. Int J Gynaecol Obstet **1987**; 25(4): 297-301.

32. Barin F, Perrin J, Chotard J, Denis F, N'Doye R, Diop Mar I, Chiron JP, Coursaget P, Goudeau A, Maupas P. Cross-sectional and longitudinal epidemiology of hepatitis B in Senegal. Prog Med Virol **1981**; 27: 148-62.

33. Bigot KA, Kodjoh N, Zohoun IS, Hountondji A, Latoundji S, Takpara L, De Souza J, Aboudou S, Alihonou E, Aguessy-Anhy B. Seroprevalence of HBsAg antigen of hepatitis B virus in pregnant women and their children. Medicine d'Afrique Noire **1992**; 39(7): 487-90.

34. Marinier E, Barrois V, Larouze B, London WT, Cofer A, Diakhate L, Blumberg BS. Lack of perinatal transmission of hepatitis B virus infection in Senegal, West Africa. J Pediatr **1985**; 106(5): 843-9.

35. Prince AM, White T, Pollock N, Riddle J, Brotman B, Richardson L. Epidemiology of hepatitis B infection in Liberian infants. Infect Immun **1981**; 32(2): 675-80.

36. Coursaget P, Yvonnet B, Chotard J, Vincelot P, Sarr M, Diouf C, Chiron JP, Diop-Mar I. Age- and sex-related study of hepatitis B virus chronic carrier state in infants from an endemic area (Senegal). J Med Virol **1987**; 22(1): 1-5.

37. Bah E, Sam O, Whittle H, Ramanakumar A, Sankaranarayanan R. Cancer survival in the Gambia, 1993−1997. In: Sankaranarayanan R, Swaminathan R, Lucas E. Cancer survival in Africa, Asia, the Caribbean and Central America (SurvCan). Lyon: International Agency for Research on Cancer, **2011**:97-100.

38. Diarra M, Konate A, Soukho A, Dicko M, Kalle A, Doumbia K, Sow H, Traore HA, Maiga MY. [Changing aspects of cirrhotic disease in a hepato-gastroenterology service in Mali]. Mali med **2010**; 25(1): 42-6.

39. Olubuyide IO. Natural history of liver cirrhosis in 116 Nigerians. East Afr Med J **1996**; 73(4): 233-5.

40. Shimakawa Y, Njai HF, Takahashi K, Berg L, Ndow G, Jeng-Barry A, Ceesay A, Tamba S, Opoku E, Taal M, Akbar SM, Arai M, D'Alessandro U, Taylor-Robinson SD, Njie R, Mishiro S, Thursz MR, Lemoine M. Hepatitis E virus infection and acute-on-chronic liver failure in West Africa: a case-control study from The Gambia. Aliment Pharmacol Ther **2016**; 43(3): 375-84.

41. Yang JD, Mohamed EA, Aziz AO, Shousha HI, Hashem MB, Nabeel MM, Abdelmaksoud AH, Elbaz TM, Afihene MY, Duduyemi BM, Ayawin JP, Gyedu A, Lohoues-Kouacou MJ, Ndam AW, Moustafa EF, Hassany SM, Moussa AM, Ugiagbe RA, Omuemu CE, Anthony R, Palmer D, Nyanga AF, Malu AO, Obekpa S, Abdo AE, Siddig AI, Mudawi HM, Okonkwo U, Kooffreh-Ada M, Awuku YA, Nartey YA, Abbew ET, Awuku NA, Otegbayo JA, Akande KO, Desalegn HM, Omonisi AE, Ajayi AO, Okeke EN, Duguru MJ, Davwar PM, Okorie MC, Mustapha S, Debes JD, Ocama P, Lesi OA, Odeghe E, Bello R, Onyekwere C, Ekere F, Igetei R, Mah'moud MA, Addissie B, Ali HM, Gores GJ, Topazian MD, Roberts LR. Characteristics, management, and outcomes of patients with hepatocellular carcinoma in Africa: a multicountry observational study from the Africa Liver Cancer Consortium. Lancet Gastroenterol Hepatol **2017**; 2(2): 103-11.

42. Umoh NJ, Lesi OA, Mendy M, Bah E, Akano A, Whittle H, Hainaut P, Kirk GD. Aetiological differences in demographical, clinical and pathological characteristics of hepatocellular carcinoma in The Gambia. Liver Int **2011**; 31(2): 215-21.

43. Ferlay J, Ervik M, Lam F, Colombet M, Mery L, Piñeros M, Znaor A, Soerjomataram I, Bray F. Global Cancer Observatory: Cancer Today. Available at: https://gco.iarc.fr/. Accessed 06/05/19.

44. Parkin DM, Whelan SL, Ferlay J, Teppo L, Thomas DB. Cancer incidence in five continents. Volume VIII. IARC Sci Publ **2002**;(155): 1-781.

45. Parkin DM, Muir CS, Whelan SL, Gao YT, Ferlay J, Powell J. Cancer incidence in five continents. Volume VI. IARC Sci Publ **1992**; (120).

46. GBD 2017 Cirrhosis Collaborators. The global, regional, and national burden of cirrhosis by cause in 195 countries and territories, 1990-2017: a systematic analysis for the Global Burden of Disease Study 2017. Lancet Gastroenterol Hepatol **2020**; 5(3): 245-66.

2. Supplementary methods

A. Data assembly

Data for model parameterisation and calibration was assembled using a 2-stage scoping review of the published literature. The scoping review aimed to identify all quantitative epidemiological and clinical data on hepatitis B virus (HBV) infection in sub-Saharan African populations, including transmission dynamics, progression of chronic HBV mono-infection, the burden and natural history of potentially HBV-associated liver disease, and the efficacy of HBV interventions. The first stage consisted of the creation of an evidence map, providing an overview of the extent and key themes in the hepatitis B epidemiology research landscape in sub-Saharan Africa. This was followed by focus on data extraction from one country to inform the development, parameterisation and calibration of a mathematical model of HBV transmission and natural history.

Search strategy

The literature search was conducted in the Medline database on 22nd February 2018 using the OvidSP interface. The search strategy, shown in **Table S2.1**, combines hepatitis B-related keywords and MeSH headings, Africa-related keywords and MeSH headings, and liver disease-related keywords and MeSH headings in sub-Saharan Africa, irrespective of hepatitis B, and was tested in a pilot search to ensure broad coverage and inclusion of previously identified relevant articles from published reviews and through expert opinion [1-3]. Additional studies suggested by clinical experts were also considered for inclusion. The references from Medline were exported and deduplicated in EndNote.

Table S2.1. Scoping review search strategy conducted in the Medline database using the OvidSP interface.

#1	exp Hepatitis B/
#2	(hepatitis b or hbv or hep B or (type b adj1 hepatitis) or hbsag or hbs-ag or hbs antigen* or "hepatitis virus* B" or hbvcoinfected or hbv-coinfected or hbv-co-infected or hbvinfect* or hbv-infect* or hbvpositive* or hbvrelated or hbvcarrier* or hiv?hbv or hbv?hiv).mp. [mp=title, abstract, original title, name of substance word, subject heading word, keyword heading word, protocol supplementary concept word, rare disease supplementary concept word, unique identifier, synonyms]
#3	1 or 2
#4	exp "AFRICA SOUTH OF THE SAHARA"/
#5	(africa* or SSA or angola* or benin* or botswana* or burkina faso* or burundi* or cabo verd* or cameroon* or cape verd* or central african republic* or chad* or comoros or comoro or comoroan or comores or comorean or (congo* not congo red) or cote divoir* or democratic republic of the congo* or djibouti* or equatorial guinea* or eritrea* or ethiopia* or gabon* or gambia* or ghan* or (guinea* not guinea pig* not guinea worm*) or guinea-bissau* or ivory coast or kenya* or lesotho* or liberia* or madagascar* or malawi* or mali or maurit* or mauritania* or mozambi* or namibia* or niger* or nigeria* or rwanda* or (sao tome and principe*) or senegal* or seychelle* or sierra leone* or somali* or south africa* or south sudan* or (sudan* not sudan blue) or swazi* or tanzania* or togo* or uganda* or zambia* or zimbabwe*).mp. [mp=title, abstract, original title, name of substance word, subject heading word, keyword heading word, protocol supplementary concept word, rare disease supplementary concept word, unique identifier, synonyms]
#6	4 or 5
#7	3 and 6
#8	Liver Cirrhosis/ or Liver Cirrhosis, Biliary/ or Liver Diseases/ or exp Hepatic Insufficiency/ or HEPATITIS/ or Hepatitis, Chronic/ or Liver Neoplasms/an, bl, cl, co, di, ec, ep, et, hi, mi, mo, pa, pc, sn or Adenoma, Liver Cell/an, bl, cl, co, di, ec, ep, et, hi, mi, mo, pa, pc, sn or Carcinoma, Hepatocellular/an, bl, cl, co, di, ec, ep, et, hi, mi, mo, pa, pc, sn
#9	(cirrhosis or cirrhotic* or liver fibrosis or liver cancer* or hepatocellular carcinoma* or liver cell carcinoma* or hepatic carcinoma* or hepatic cell carcinoma* or hepatocellular cancer or liver disease* or liver failure* or liver inflammation* or liver necroinflammation*).mp. [mp=title, abstract, original title, name of substance word, subject heading word, keyword heading word, protocol supplementary concept word, rare disease supplementary concept word, unique identifier, synonyms]
#10	8 or 9
#11	6 and 10
#12	7 or 11
#13	12 and "Case Reports" [Publication Type]
#14	12 and "Letter" [Publication Type]
#15	12 not 13 not 14

Study selection and inclusion criteria

Initial study selection for the evidence map was based on screening of titles and abstracts by one reviewer. To be included, abstracts had to present primary data and be broadly relevant to at least one of the aspects of the research aim. Articles were therefore excluded because of being a different publication type (e.g. literature reviews, case reports, letters, etc.), if their content was not related to HBV or potentially HBV-associated liver disease, covered basic research (i.e. experimental studies on biological processes), was conducted in non-human animals, or was not conducted in a sub-Saharan African population. Articles for which no abstract could be found were also excluded, as this was usually the case for older publications that were not available online. Additionally, although the evidence map is based on primary

data, other original research articles (systematic reviews, modelling studies and economic evaluations) were reviewed separately. No language or time restrictions were applied.

Geographical scope. The study setting of interest were sub-Saharan African populations, including natural history data from African migrants abroad. Studies set in migrants were included if their infection was deemed representative of those in sub-Saharan Africa, e.g. if they were likely to have acquired infection in sub-Saharan Africa (born in Africa for example) or reported to be infected with genotype E or A. Studies in populations referred to only as "African American" were excluded.

Study populations. Inclusion or exclusion of different study populations differed by the outcome of interest. Studies reporting natural history-related outcomes were included if they were set in a population infected with HBV. Studies of liver disease patients of mixed or unknown aetiologies were also included because a previous literature review identified no prospective studies on disease progression in chronic HBV infection in Africa [4], but studies of liver disease attributed entirely to a cause other than HBV (e.g. hepatitis C) were excluded. Studies reporting data related to transmission or infection incidence were included unless they were set in specific risk groups for HBV infection, e.g. healthcare workers or disease patients at risk of iatrogenic transmission.

Outcomes. HBsAg seroprevalence studies, with or without assessment of risk factors for HBV exposure or infection, were not systematically included because of multiple existing systematic reviews and meta-analyses on this [5]. For the same reason, cross-sectional studies exclusively reporting co-infection prevalence (e.g. most commonly with HIV, hepatitis C or hepatitis delta virus) were also excluded [6, 7]. in addition to studies on anti-HBc IgM prevalence (indicating acute infection). All other outcomes relevant to the research question were included.

Data extraction for the model

Of 5972 individual studies found in the search, 759 studies on epidemiology or natural history met the initial inclusion criteria for the evidence map as well as for potential use in the model in the title-and-abstract screening (**Figure S2.1**). Analysis of the evidence map, including full-text screening and extraction of data from a subset of the longitudinal studies, allowed to geographically and topically focus the second stage of the scoping review. Having identified The Gambia as the country with among the largest number of studies and the highest-quality data, including a longitudinal study on disease progression in chronic HBV carriers with long-term follow-up [8], cross-sectional data from The Gambia and longitudinal data on disease progression from West Africa were extracted from 53 articles for use in a mathematical model of HBV transmission and natural history. Systematic reviews spanning multiple countries or

regions identified in the evidence map additionally provided data sources of mother-to-child transmission risk [2] and modelled country-specific incidence rates of hepatocellular carcinoma (HCC) [9, 10] and cirrhosis mortality [11, 12].

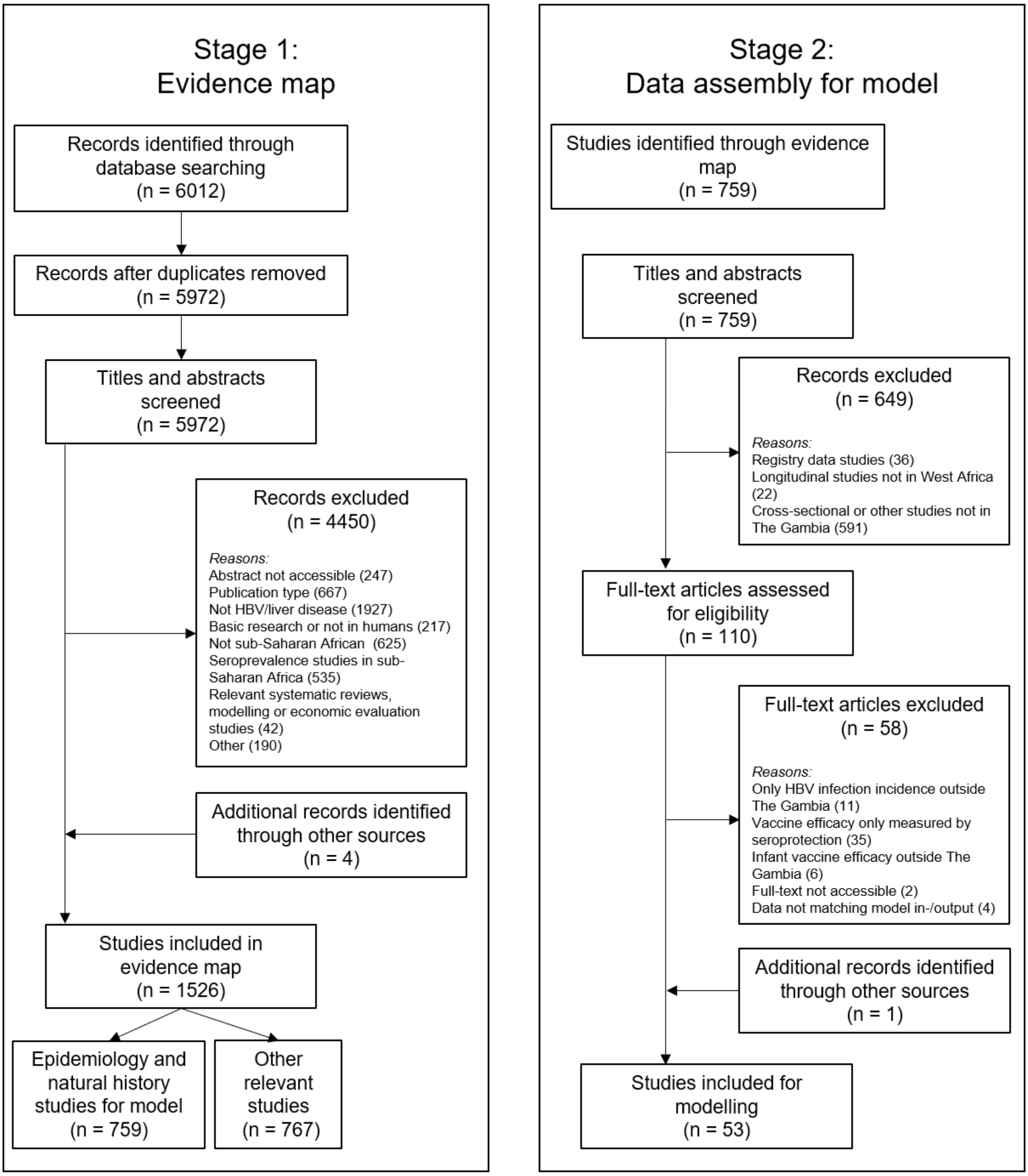

Figure S2.1. Flowchart of the two-stage scoping review. Of 53 studies meeting inclusion criteria for use in the model, 12 studies were removed in data processing.

Data from the included studies were extracted stratified by age, sex and time where available. The mean or median age of study participants was extracted where reported, or alternatively the mid-point of the age range or median based on the age distribution. Additional information

on study design, setting and other relevant methods were also extracted specific to the type of data. If the included study did not specify the time period of data collection, it was assumed based on recruitment and maximum follow-up time or to be 2 years before the publication date. In studies of liver disease, all cases of primary liver cell carcinoma were assumed to represent HCC. Where possible, cirrhosis patients were classified as compensated or decompensated, based on appropriate definitions in the paper or on the reported prevalence of different clinical symptoms or scores.

Due to the large quantity of different types of data and outcomes, quality was not explicitly scored for each study. However, key considerations relevant to the different outcomes were taken into account and some studies with methodological flaws were excluded following extraction (e.g. [13]). Seven identified studies on the impact of HBV treatment were either used to inform the treatment model structure [14, 15], or excluded because they involved outdated treatment regimens. All data that could not be meaningfully applied in the model structure, such as measurement of viral load or alanine aminotransferase (ALT) in isolation or on occult infection, were also excluded. Due to the overlap in papers of the long-term HBV studies conducted in The Gambia, it was not always possible to ascertain or ensure independence of the included datapoints. However, where this was of concern, the included datapoints were chosen based on containing more information (e.g. age- or sex-strata), being more recent or being based on the largest sample size.

95% confidence intervals (CI) were calculated for all extracted datapoints according to standard statistical formula where these were not reported in the original study, except for survival curves and for rates where the necessary information was not available. For proportions, the binomial 95% CI was calculated using the Wilson method, as many of the datapoints were based on small sample sizes. For rates, the 95% CI were calculated using the normal approximation of the Poisson distribution [16].

B. Model development

Model structure: overview and key features

Evidence from the scoping review was used to develop a dynamic deterministic compartmental model of HBV transmission and natural history adapted to the West African epidemic in *R* statistical software [17]. The model was adapted from an existing structure [3] and fully structured by age and sex to represent the long-lasting duration and age- and sex-dependent processes in development of chronic HBV infection and liver disease. The model was formulated using partial differential equations with respect to time and age, which were solved using the *deSolve* package.

The overarching transmission structure of the model is composed of compartments for susceptible, chronically infected and immune/recovered populations, with chronic infection further split into different clinical stages. The Susceptible-Infected-Recovered structure can be mapped to serological markers as shown in **Table S2.2**. Acute infection was not incorporated because it is short-lived and assumed to contribute little to disease burden in sub-Saharan Africa [18, 19]. The Recovered/Immune compartment comprises recovery from acute infection (as opposed to progression to chronic infection), clearance of the hepatitis B surface antigen (HBsAg) serological marker in chronic infection (serological recovery), or, in the post-vaccination time period, vaccine-induced immunity. Immunity from natural infection or vaccination is assumed to be lifelong.

Table S2.2. Seromarkers of hepatitis B virus (HBV) exposure and infection.

Compartment	Serology of individuals in the compartment	Clinical profile
**Susceptible**	Negative for HBsAg and anti-HBc and anti-HBs.	Never exposed to HBV.
**Chronic infection compartments**	Positive for HBsAg and anti-HBc, negative for anti-HBs. Some compartments are positive for HBeAg.	Chronically infected following exposure to HBV.
**Recovered/Immune (before vaccine introduction)**	Positive for anti-HBc and anti-HBs, negative for HBsAg.	Ever exposed to HBV. May reflect recovery from acute infection without development of chronic infection or HBsAg loss/serological recovery after chronic infection.
**Recovered/Immune (after vaccine introduction)**	Positive for anti-HBs, negative for HBsAg. May be positive for anti-HBc (following exposure, but not after vaccination).	Ever exposed to HBV or ever vaccinated against HBV.

Anti-HBc = hepatitis B core antibody, anti-HBs = hepatitis B surface antibody, HBeAg = hepatitis B e antigen, HBsAg = hepatitis B surface antigen.

Chronic HBV infection in the absence of treatment is divided into compartments for HBeAg-positive infection (*Ie*), HBeAg-positive chronic hepatitis B (CHB) (*De*), HBeAg-negative infection (*In*), HBeAg-negative CHB (*Dn*), Compensated cirrhosis (*C*), Decompensated cirrhosis (*D*) and Hepatocellular carcinoma (*H*) (**Table S1.3**). Abbreviations of the clinical stages distinguish infection (*I*) and disease (*D*) stages which are positive (*e*) or negative (*n*) for the hepatitis B e antigen (HBeAg). Equations for transition rates and justification of the assumptions in the natural history model of chronic HBV infection are detailed in the *Natural history of chronic HBV infection* section. Partial differential equations and parameter names and values are detailed in the *Model equations* section and in **Table S1.4**-**Table S1.5**. Transition rates in the model can vary by age (*a*), time (*t*) or sex (index *g*). All compartments experience the same age-, sex- and time-specific background mortality rate $\mu_g(a, t)$ and net migration rate $n_g(a, t)$.

Births occur into the Susceptible or the HBeAg-positive infection compartment, the latter representing mother-to-child transmission. Susceptible individuals are infected at the force of infection $\lambda(a, t)$, of which an age-dependent proportion $p(a)$ develop chronic carriage following acute infection, and a proportion $1 - p(a)$ of infected people recover from acute infection and become immune to reinfection. The force of infection varies by age to account for horizontal transmission in sub-Saharan Africa historically occurring mainly among young children [18], but potential heterogeneity in the risk of infection by population group or sub-national location was not modelled. Infectiousness depends on a carrier's HBeAg serostatus [3, 20].

Sequelae and mortality from chronic HBV infection

Among carriers with chronic HBV infection, CHB compartments contain carriers with underlying liver fibrosis which can progress to compensated cirrhosis, marking the onset of the liver disease pathway to decompensation and HCC. While HCC is often a consequence of liver scarring and inflammation, it can also occur as a result of viral DNA integration into liver cells in chronic HBV infection. Therefore, all chronic infection compartments can also progress to HCC at different rates without development of cirrhosis. HBV-related mortality occurs from the compensated cirrhosis, decompensated cirrhosis and hepatocellular carcinoma compartments. From a clinical perspective, symptoms often only occur once patients reach the decompensated cirrhosis or HCC stage. HBV-related mortality from acute infection or other sequelae that may lead to death, such as renal disease, were not accounted for as these are rare compared to liver disease mortality [21, 22].

HBV interventions in The Gambia

In The Gambia, 3-dose HBV infant vaccination was introduced into the national routine vaccination schedule within the Expanded Programme on Immunizations (EPI) in 1990 [23]. In the model, susceptible children are vaccinated from the year of vaccine introduction onwards, with vaccination assumed to occur at historical coverage levels reported by the World Health Organization (WHO) [24].

We assumed infant vaccination to be the only currently implemented HBV intervention in The Gambia, and that historical or current coverage of timely birth dose vaccination and antiviral treatment is negligible. Though the EPI schedule in The Gambia includes an official birth dose vaccination policy, the model structure makes the assumption that the birth dose vaccine is only effective at preventing mother-to-child transmission (MTCT) if administered within 24 hours of birth. This simplified assumption was based on indirect evidence for an increasing risk of infection with child's age at first dose between 1-4 days [25], and further observations on the need for timely administration of hepatitis B immune globulin (HBIG) [26], as there is no direct evidence from clinical trials on the efficacy of later administration of the birth dose vaccine. At the time of model development, country-reported estimates of birth dose coverage were high, but did not distinguish between a "timely" (within 24 hours of birth) and delayed administration [27]. In the most recent Gambian study on the topic, the coverage of timely delivery of the birth dose was reported to be very low between 2004 and 2014, with only 1.1% of newborns in the Farafenni region receiving the HBV vaccine at birth [28]. Similarly, in 2016, only 7% of infants born in the sampled healthcare facilities received the birth dose vaccine within 24 hours [29]. In the model, the efficacy of early vaccination (e.g. delayed birth dose vaccination) in preventing horizontal infections is captured in the application of the infant vaccine, which in itself produced model fits consistent with the low HBsAg prevalence observed in young children. New data stratified by timing of administration has since shown that scale-up of timely birth dose vaccination is already underway [27], estimating a 35% coverage in The Gambia in 2019, but this is unlikely to affect simulations of a treatment programme in adults in 2020.

Non-HBV-related treatment for early-stage liver disease, such as liver transplantation, was not included in the model as access to this is extremely limited in sub-Saharan Africa [30]. A small number of HBV carriers in The Gambia are currently taking antiviral therapy against HBV, such as study participants in the PROLIFICA screen-and-treat programme [15] and some receiving treatment for HIV co-infection [31]. However, in general access to HBV testing and treatment is still very limited, with estimates of less than 1% of HBV carriers being diagnosed and less than 1% of treatment-eligible carriers receiving treatment in The Gambia in 2016 [32].

For this reason, large-scale diagnosis and treatment for HBV infection were modelled only as hypothetical future interventions.

Modelling screening and treatment

Carriers in the HBeAg-positive infection, HBeAg-positive and -negative CHB and the compensated and the decompensated cirrhosis stages would be eligible for HBV-specific treatment according to European liver association guidelines [22], to reduce the progression of liver disease and development of HCC. For these carriers, the option of lifelong antiviral therapy with tenofovir disoproxil fumarate (TDF) was added to the model.

To investigate the impact of a hypothetical mass testing and treatment intervention, the 7 chronic infection compartments as well as the Recovered compartment are stratified by HBsAg screening status (undiagnosed vs. diagnosed), with diagnosed states denoted by an S prefix (e.g. *SIe* for diagnosed HBeAg-positive infection). Diagnosed compartments comprise chronic HBV carriers with known HBsAg status but not (yet) initiated on treatment because they did not meet the criteria for treatment initiation at initial clinical assessment. Other than potential progression to treated states, these diagnosed carrier compartments experience the same transitions as undiagnosed states. As people can only enter the diagnosed state following the intervention, no new infections occur within these compartments. Transition into the *SR* compartment does not occur through the screening intervention but represents those individuals who have lost HBsAg following their diagnosis in the screening interventions.

Carriers in the subset of disease states eligible for antiviral therapy are initiated on treatment as a result of the screening intervention by moving into the corresponding treated compartments. The treatment model consists of compartments for Treated HBeAg-positive infection (*TIe*), Treated CHB (*TCHB*), Treated compensated cirrhosis (*TC*), Treated decompensated cirrhosis (*TD*), Hepatocellular carcinoma following treatment (*TH*) and Recovery following treatment (*TR*). The mechanism of implementing the screening intervention and the treatment model structure and parameterisation are further described in the *Simulated interventions: antiviral treatment* section.

Model equations

Partial differential equations

Table S2.3. List of model compartments and their abbreviations.

Compartment label	Definition
**Natural history compartments**	
S	Susceptible
Ie	HBeAg-positive infection
De	HBeAg-positive chronic hepatitis B (CHB)
In	HBeAg-negative infection
Dn	HBeAg-negative chronic hepatitis B (CHB)
C	Compensated cirrhosis
D	Decompensated cirrhosis
H	Hepatocellular carcinoma (HCC)
R	Recovered/immune
**Diagnosed (untreated) compartments**	
SIe	Screened HBeAg-positive infection
SDe	Screened HBeAg-positive chronic hepatitis B (CHB)
SIn	Screened HBeAg-negative infection
SDn	Screened HBeAg-negative chronic hepatitis B (CHB)
SC	Screened Compensated cirrhosis
SD	Screened Decompensated cirrhosis
SH	Screened Hepatocellular carcinoma (HCC)
SR	Recovered/immune after screening
**Treated compartments**	
TIe	Treated HBeAg-positive infection
TCHB	Treated chronic hepatitis B (CHB)
TC	Treated Compensated cirrhosis
TD	Treated Decompensated cirrhosis
TH	Hepatocellular carcinoma (HCC) after treatment initiation
TR	Recovered/immune after treatment initiation

The partial differential equations describing the rate of change in each model compartment with respect to time *t* and age *a* are given below. Index *g* represents two discrete sex strata (*m* = male and *f* = female). In all equations, $\partial a = \partial t =$ 0.5 years. The abbreviations for different compartments used in the equations are summarised in **Table S2.3**. All calibrated and fixed parameters and their values are listed in **Table S2.4** and **Table S2.5**, respectively.

To solve the partial differential equations using the *deSolve* package in R, the age variable was discretised into 0.5 year groups from 0 to 100 to obtain a set of ordinary differential equations that can be solved with the ode.1D function using the method of lines. The aging process in the model (like all transitions in the model) therefore follows an exponential distribution, whereby all individuals within a 0.5 year age group have the same chance of aging into the next age group. The differential equations were solved numerically using the *lsoda* integration algorithm.

Partial differential equations for the natural history compartments:

$$\frac{\partial S(a,t)}{\partial t}+\frac{\partial S(a,t)}{\partial a}=-\lambda(a,t)\,S(a,t)-v(a,t)\,S(a,t)-\mu_g(a,t)\,S(a,t)+n_g(a,t)\,S(a,t)$$

$$\frac{\partial Ie(a,t)}{\partial t}+\frac{\partial Ie(a,t)}{\partial a}$$
$$=p(a)\lambda(a,t)\,S(a,t)-f^{Ie\to De}(a)\,Ie(a,t)-f_g^{Ie\to H}(a)\,Ie(a,t)-\mu_g(a,t)\,Ie(a,t)$$
$$+\,n_g(a,t)\,Ie(a,t)$$

$$\frac{\partial De(a,t)}{\partial t}+\frac{\partial De(a,t)}{\partial a}$$
$$=f^{Ie\to De}(a)\,Ie(a,t)-f^{De\to In}(a)\,De(a,t)-r^{De\to Dn}\,De(a,t)$$
$$-\,f_i^{De\to C}(a)\,De(a,t)-f_i^{De\to H}(a)\,De(a,t)-\mu_g(a,t)\,De(a,t)+n_g(a,t)\,De(a,t)$$

$$\frac{\partial In(a,t)}{\partial t}+\frac{\partial In(a,t)}{\partial a}$$
$$=f^{De\to In}(a)\,De(a,t)-r^{In\to Dn}\,In(a,t)-\,f^{In\to R}(a)\,In(a,t)-f_g^{In\to H}(a)\,In(a,t)$$
$$-\,\mu_g(a,t)\,In(a,t)+n_g(a,t)\,In(a,t)$$

$$\frac{\partial Dn(a,t)}{\partial t}+\frac{\partial Dn(a,t)}{\partial a}$$
$$=r^{De\to Dn}\,De(a,t)+r^{In\to Dn}\,In(a,t)-f_g^{Dn\to C}\,Dn(a,t)-f_g^{Dn\to H}(a)\,Dn(a,t)$$
$$-\,\mu_g(a,t)\,Dn(a,t)+n_g(a,t)\,Dn(a,t)$$

$$\frac{\partial C(a,t)}{\partial t}+\frac{\partial C(a,t)}{\partial a}$$
$$=f_g^{Dn\to C}\,Dn(a,t)+f_g^{De\to C}(a)\,De(a,t)-r^{C\to D}C(a,t)-f_g^{C\to H}(a)\,C(a,t)$$
$$-\,\mu^C\,C(a,t)-\mu_g(a,t)\,C(a,t)+n_g(a,t)\,C(a,t)$$

$$\frac{\partial D(a,t)}{\partial t}+\frac{\partial D(a,t)}{\partial a}$$
$$=r^{C\to D}\,C(a,t)-r^{D\to H}\,D(a,t)-\mu^D\,D(a,t)-\mu_g(a,t)\,D(a,t)+n_g(a,t)\,D(a,t)$$

$$\frac{\partial H(a,t)}{\partial t}+\frac{\partial H(a,t)}{\partial a}$$
$$=f_g^{Ie\to H}(a)\,Ie(a,t)+f_g^{De\to H}(a)\,De(a,t)+f_g^{In\to H}(a)\,In(a,t)$$
$$+\,f_g^{Dn\to H}(a)\,Dn\,(a,t)+f_g^{C\to H}(a)\,C(a,t)+\,r^{D\to H}\,D(a,t)-\mu^H\,H(a,t)$$
$$-\,\mu_g(a,t)\,H(a,t)+n_g(a,t)\,H(a,t)$$

$$\frac{\partial R(a,t)}{\partial t}+\frac{\partial R(a,t)}{\partial a}$$
$$= \left(1-p(a)\right)\lambda(a,t)\,S(a,t)+f^{In\to R}(a)\,In(a,t)+\ v(a,t)\,S(a,t)$$
$$-\mu_g(a,t)\,R(a,t)+n_g(a,t)\,R(a,t)$$

Partial differential equations for the diagnosed (untreated) compartments:

$$\frac{\partial SIe(a,t)}{\partial t}+\frac{\partial SIe(a,t)}{\partial a}$$
$$= -f^{Ie\to De}(a)\,SIe(a,t)-f_g^{Ie\to H}(a)\,SIe(a,t)-r^{SIe\to T}(a)\,SIe(a,t)$$
$$-\mu_g(a,t)\,SIe(a,t)+n_g(a,t)\,SIe(a,t)$$

$$\frac{\partial SDe(a,t)}{\partial t}+\frac{\partial SDe(a,t)}{\partial a}$$
$$= f^{Ie\to De}(a)\,SIe(a,t)-f^{De\to In}(a)\,SDe(a,t)-r^{De\to Dn}\,SDe(a,t)$$
$$-f_i^{De\to C}(a)\,SDe(a,t)-f_i^{De\to H}(a)\,SDe(a,t)-r^{T}(a)SDe(a,t)$$
$$-\mu_g(a,t)\,SDe(a,t)+n_g(a,t)\,SDe(a,t)$$

$$\frac{\partial SIn(a,t)}{\partial t}+\frac{\partial SIn(a,t)}{\partial a}$$
$$= f^{De\to In}(a)\,SDe(a,t)-r^{In\to Dn}\,SIn(a,t)-\ f^{In\to R}(a)\,SIn(a,t)$$
$$-f_g^{In\to H}(a)\,SIn(a,t)-\mu_g(a,t)\,SIn(a,t)+n_g(a,t)\,SIn(a,t)$$

$$\frac{\partial SDn(a,t)}{\partial t}+\frac{\partial SDn(a,t)}{\partial a}$$
$$= r^{De\to Dn}\,SDe(a,t)+r^{In\to Dn}\,SIn(a,t)-f_g^{Dn\to C}\,SDn(a,t)$$
$$-f_g^{Dn\to H}(a)\,SDn(a,t)-r^{T}(a)\,SDn(a,t)-\mu_g(a,t)\,SDn(a,t)$$
$$+n_g(a,t)\,SDn(a,t)$$

$$\frac{\partial SC(a,t)}{\partial t}+\frac{\partial SC(a,t)}{\partial a}$$
$$= f_g^{Dn\to C}\,SDn(a,t)+f_g^{De\to C}(a)\,SDe(a,t)-r^{C\to D}SC(a,t)-f_g^{C\to H}(a)\,SC(a,t)$$
$$-r^{T}(a)\,SC(a,t)-\mu^{C}\,SC(a,t)-\mu_g(a,t)\,SC(a,t)+n_g(a,t)\,SC(a,t)$$

$$\frac{\partial SD(a,t)}{\partial t}+\frac{\partial SD(a,t)}{\partial a}$$
$$= r^{C\to D}\,SC(a,t)-r^{D\to H}\,SD(a,t)-r^{T}(a)\,SD(a,t)-\mu^{D}\,SD(a,t)$$
$$-\mu_g(a,t)\,SD(a,t)+n_g(a,t)\,SD(a,t)$$

$$\frac{\partial SH(a,t)}{\partial t}+\frac{\partial SH(a,t)}{\partial a}$$
$$= f_g^{Ie\rightarrow H}(a)\ SIe(a,t) + f_g^{De\rightarrow H}(a)\ SDe(a,t) + f_g^{In\rightarrow H}(a)\ SIn(a,t)$$
$$+ f_g^{Dn\rightarrow H}(a)\ SDn\ (a,t) + f_g^{C\rightarrow H}(a)\ SC(a,t) +\ r^{D\rightarrow H}\ SD(a,t) - \mu^{H}\ SH(a,t)$$
$$- \mu_g(a,t)\ SH(a,t) + n_g(a,t)\ SH(a,t)$$

$$\frac{\partial SR(a,t)}{\partial t}+\frac{\partial SR(a,t)}{\partial a} = f^{In\rightarrow R}(a)\ SIn(a,t) - \mu_g(a,t)\ SR(a,t) + n_g(a,t)\ SR(a,t)$$

Partial differential equations for the treated carrier compartments:

$$\frac{\partial TIe(a,t)}{\partial t}+\frac{\partial TIe(a,t)}{\partial a}$$
$$= r^{SIe\rightarrow T}(a) SIe(a,t) - m^{TIe\rightarrow TH} \times f_g^{Ie\rightarrow H}(a)\ TIe(a,t) - \mu_g(a,t)\ TIe(a,t)$$
$$+ n_g(a,t)\ TIe(a,t)$$

$$\frac{\partial TCHB(a,t)}{\partial t}+\frac{\partial TCHB(a,t)}{\partial a}$$
$$= r^{T}(a)\ SDe(a,t) +\ r^{T}(a)\ SDn(a,t) -\ f^{In\rightarrow R}(a)\ TCHB(a,t)$$
$$- m^{TCHB\rightarrow TH} \times f_g^{Dn\rightarrow H}(a)\ TCHB(a,t) - \mu_g(a,t)\ TCHB(a,t)$$
$$+ n_g(a,t)\ TCHB(a,t)$$

$$\frac{\partial TC(a,t)}{\partial t}+\frac{\partial TC(a,t)}{\partial a}$$
$$= r^{T}(a)\ SC(a,t) - m^{TC\rightarrow TH} \times f_g^{C\rightarrow H}(a)\ TC(a,t) - \mu_g(a,t)\ TC(a,t)$$
$$+ n_g(a,t)\ TC(a,t)$$

$$\frac{\partial TD(a,t)}{\partial t}+\frac{\partial TD(a,t)}{\partial a}$$
$$= r^{T}(a)\ SD(a,t) - m^{TD\rightarrow TH} \times r^{D\rightarrow H}\ TD(a,t) - \mu^{TD}\ TD(a,t) - \mu_g(a,t)\ TD(a,t)$$
$$+ n_g(a,t)\ TD(a,t)$$

$$\frac{\partial TH(a,t)}{\partial t} + \frac{\partial TH(a,t)}{\partial a}$$
$$= m^{TCHB \to TH} \times f_g^{Dn \to H}(a)\, TCHB(a,t) + m^{TC \to TH} \times f_g^{C \to H}(a)\, TC(a,t)$$
$$+ \ m^{TD \to TH} \times r^{D \to H}\, TD(a,t) - \mu^H\, TH(a,t) - \mu_g(a,t)\, TH(a,t)$$
$$+ n_g(a,t)\, TH(a,t)$$

$$\frac{\partial TR(a,t)}{\partial t} + \frac{\partial TR(a,t)}{\partial a} = f^{In \to R}(a)\, TCHB(a,t) - \mu_g(a,t)\, TR(a,t) + n_g(a,t)\, TR(a,t)$$

Equations for the mass screening and treatment programme

Removals from undiagnosed compartments (due to treatment and diagnosis without treatment):

$$X_{eligible}(a+da,t+dt) = X_{eligible}(a,t) - (p_{screen} \times p_{assess} \times p_{treat})\, X_{eligible}(a,t)$$

Where $X_{eligible} \in \{De, Dn, C, D\}$

$$X_{ineligible}(a+da,t+dt) = X_{ineligible}(a,t) - (p_{screen} \times p_{assess})\, X_{ineligible}(a,t)$$

Where $X_{ineligible} \in \{In, H\}$

$$Ie(a+da,t+dt) = \begin{cases} Ie(a,t) - (p_{screen} \times p_{assess})\, Ie(a,t) & a \leq 30\ years \\ Ie(a,t) - (p_{screen} \times p_{assess} \times p_{treat}) Ie(a,t) & a > 30\ years \end{cases}$$

Movement to diagnosed compartments:

$$SIe(a+da,t+dt) = \begin{cases} SIe(a,t) + (p_{screen} \times p_{assess}) Ie(a,t) & a \leq 30\ years \\ SIe(a,t) & a > 30\ years \end{cases}$$

$$SIn(a+da,t+dt) = SIn(a,t) + (p_{screen} \times p_{assess}) In(a,t)$$

$$SH(a+da,t+dt) = SH(a,t) + (p_{screen} \times p_{assess}) H(a,t)$$

Movement to treated compartments:

$$TIe(a+da,t+dt) = \begin{cases} TIe(a,t) & a \leq 30\ years \\ TIe(a,t) + (p_{screen} \times p_{assess} \times p_{treat}) Ie(a,t) & a > 30\ years \end{cases}$$

$$TCHB(a+da,t+dt) = TCHB(a,t) + (p_{screen} \times p_{assess} \times p_{treat})\big(De(a,t) + Dn(a,t)\big)$$

$$TC(a+da,t+dt) = TC(a,t) + (p_{screen} \times p_{assess} \times p_{treat}) C(a,t)$$

$$TD(a+da,t+dt) = TD(a,t) + (p_{screen} \times p_{assess} \times p_{treat}) D(a,t)$$

(All equations above apply:) At time $t = T_{screen}$ and age $a \in A_{screen}$

$da = dt =$ 0.5 years

Model parameters

List of calibrated model parameters with prior distributions

Table S2.4. Description of model parameters varied in the calibration, their prior distribution and sources for allocation of priors. Mean and approximate range of prior distributions are given for ease of interpretation. Rates are in units of per person-year.

Parameter	Description	Prior	Justification and sources
$\boldsymbol{\beta_1}$	Horizontal transmission coefficient for transmission among children aged 0.5-5 years	Uniform(0.03,0.7)	Assumption
$\boldsymbol{\beta_2}$	Horizontal transmission coefficient for transmission among children aged 5-15 years and between children 0.5-5 years old and 5-15 years	Uniform(0, β_1)	Assumption
$\boldsymbol{\beta_3}$	Horizontal transmission coefficient for transmission among adults aged 15+ years and between adults and 5-15-year-old children	Uniform(0, β_1)	Assumption
$\boldsymbol{\alpha}$	Relative infectiousness of HBeAg-positive compared to HBeAg-negative carriers in horizontal transmission	Uniform(1.5,10)	HBeAg is a proxy for viral load and associated with higher infectivity. Cannot be directly measured but informed by various proxy values from the literature: Keane, 2016; Shimakawa, 2014; Gerlich, 2014, Salkic, 2007 [2, 33-35]
$\boldsymbol{v_n}$	Probability of mother-to-child transmission from HBeAg-negative mother at birth	Beta(1.5,13.5) [mean 0.1, range 0-0.5]	Prior incorporates knowledge from Asia due to low data quality in sub-Saharan Africa: Keane, 2016 [2]
$\boldsymbol{v_e}$	Probability of mother-to-child transmission from HBeAg-positive mother at birth	Uniform(v_n,0.9)	Prior incorporates knowledge from Asia due to low data quality in sub-Saharan Africa: Keane, 2016 [2]
$\boldsymbol{p_0}$	Probability of developing chronic carriage following acute infection at age 0 (perinatal transmission)	Beta(10.49,1.3) [mean 0.89, range 0.5-1]	Edmunds, 1993 [1]
$\boldsymbol{c_r}$	Coefficient r of exponential decay function for age-specific risk of becoming a chronic carrier after acute infection	Normal(0.65,0.1) [mean 0.65, range 0.25-1]	Edmunds, 1993 [1] and range determined using empirical approach

Parameter	Description	Prior	Justification and sources
c_s	Coefficient s of exponential decay function for age-specific risk of becoming a chronic carrier after acute infection	Normal(0.46,0.1) [mean 0.46, range 0.05-0.9]	Edmunds, 1993 [1] and range determined using empirical approach
$r^{Ie \to De}$	Annual rate of progression from HBeAg-positive infection to HBeAg-positive CHB in 0-year olds	Gamma(3.63,26.27) [mean 0.1, range 0.01-0.6]	Based on value from Gambia cost-effectiveness model in Nayagam, 2016 [36] and biological plausibility
$r^{De \to In}$	Annual rate of progression from HBeAg-positive CHB to HBeAg-negative infection in 0-year olds	Uniform(0,1)	Assumption based on prevalence data suggesting that average duration of *De* phase is shorter than *Ie* phase: Shimakawa, 2016 [8]
c_{eag}	Rate parameter of exponential growth function for age-specific progression through the HBeAg-positive compartments	Uniform(0,0.01)	Shimakawa, 2016 [8]
$r^{De \to C}$	Annual rate of progression from HBeAg-positive CHB to compensated cirrhosis in women aged ≥ t_c years	Uniform(0.005,0.05)	Assumption based on data from both sexes: Nayagam, 2016; Lin, 2007 [3, 37]
t_c	Minimum age for progression from HBeAg-positive CHB to compensated cirrhosis	Discrete uniform(0,15)	Shimakawa, 2016 [8], expert opinion
c_{sag}	Slope parameter of linear function for age-specific rate of HBsAg loss	Normal(0.0004106, 0.00005) [mean 0.00041, range 0.0002-0.0006]	Based on linear model fitted to age-specific rates of HBsAg loss: Shimakawa, 2016 [8]
$r^{Dn \to C}$	Annual rate of progression from HBeAg-negative CHB to compensated cirrhosis in women	Gamma(1.23, 22.33) [mean 0.0105, range 0-0.5]	Assumption based on data from men/both sexes: Nayagam, 2016; Hsu, 2002 [36, 38]
m_{cirr}	Rate ratio for progression from HBeAg-positive CHB and HBeAg-negative CHB to compensated cirrhosis for men compared to women	Normal(3.5,4) truncated at 1 [mean 5, range 1-20]	REVEAL cohort: Chen, 2011; Sun, 2017 [39, 40]
m_{hcc}	Rate ratio for progression to HCC (from all carrier compartments except for decompensated cirrhosis) for men compared to women	Normal(3.5,4) truncated at 1 [mean 5, range 1-20]	Chen, 2011; Sun, 2017; Raffetti, 2016 [39-41]
c_{hcc}	Coefficient of quadratic shift function for age- specific progression from HBeAg-negative infection to HCC in women	Uniform(0.0001-0.0003)	Assumption based on data from both sexes: Raffetti, 2016 [41]

Parameter	Description	Prior	Justification and sources
t_h	Minimum age for progression to HCC (from all carrier compartments except for decompensated cirrhosis)	Discrete uniform(0,15)	Kirk, 2004 [42], expert opinion
$m^{Ie \to H}$	Rate ratio for progression to HCC from HBeAg-positive infection compared to HBeAg-negative infection	Normal(6,3) truncated at 1 [mean 6, range 1-19]	Rate ratio for increased viral load from REVEAL cohort: Chen, 2006 [43]
$m^{C \to H}$	Rate ratio for progression to HCC from compensated cirrhosis compared to HBeAg-negative infection	Uniform($m^{Ie \to H}$,100)	Empirical assumption based on Raffetti, 2016 [41]
$m^{Dn \to H}$	Rate ratio for progression to HCC from HBeAg-negative CHB compared to HBeAg-negative infection	Uniform($m^{Ie \to H}$, $m^{C \to H}$)	Expert opinion and Chen, 2006 [43]
$m^{De \to H}$	Rate ratio for progression to HCC from HBeAg-positive CHB compared to HBeAg-negative infection	Uniform($m^{Dn \to H}$, $m^{C \to H}$)	Expert opinion and Chen, 2006 [43]
$r^{De \to Dn}$	Annual rate of progression from HBeAg-positive CHB to HBeAg-negative CHB	Gamma(1.22, 44.20) [mean 0.005, range 0-0.2]	Based on value from Gambia cost-effectiveness model in Nayagam, 2016 [36] and EASL [22]
$r^{In \to Dn}$	Annual rate of progression from HBeAg-negative infection to HBeAg-negative CHB	Gamma(3.12, 141.30) [mean 0.016, range $9*10^{-6}$-0.15]	Nayagam, 2016; Boglione, 2014; Chu, 2009 [3, 44, 45]
$r^{C \to D}$	Annual rate of decompensation of cirrhosis	Gamma(17.94,423.61) [mean 0.04, range 0.01-0.09]	Nayagam, 2016; D'Amico, 2006 [36, 46]
$r^{D \to H}$	Annual rate of progression from decompensated cirrhosis to HCC	Gamma(3.08, 29.76) [mean 0.07, range 0.003-0.6]	Mittal, 2017; Hui, 2002; Thiele, 2014 [47-49]
μ^C	Annual HBV-related mortality rate from compensated cirrhosis	Gamma(4.25, 124.91) [mean 0.026, range 0-0.1]	Lin, 2005; D'Amico, 2006 [4, 46]
μ^D	Annual HBV-related mortality rate from decompensated cirrhosis	Gamma(2.18, 1.18) [mean 1, range 0-10]	Nayagam, 2016; D'Amico, 2006; Attia, 2008 [36, 46, 50]
μ^H	Annual HBV-related mortality rate from HCC	Gamma(2.18, 1.18) [mean 1, range 0-10]	Nayagam, 2016; Olubuyide, 1992 [36, 51]
v_{eff}	Vaccine efficacy against chronic infection	Beta(7.07, 0.37) [mean 0.95, range 0.3-1]	Peto, 2014; Fortuin, 1993; Viviani, 1999; Van der Sande, 2007; Mendy, 2013; World Health Organization, 2017 [52-57]

CHB = chronic hepatitis B, HCC = hepatocellular carcinoma.

List of fixed model parameters

Table S2.5. Description of fixed model parameters, their values and source. Rates are in units of per person-year.

Parameter	Description	Value	Justification and sources
**Parameters relating to demography**			
$\boldsymbol{\mu_g(a,t)}$	Annual background mortality rate	Varies by age, sex and time	UN World Population Prospects [58]
$\boldsymbol{f(a,t)}$	Annual fertility rate of women	Varies by age and time	
$\boldsymbol{SP_g}$	Sex-specific proportion of births	0.493 for women, 0.507 for men	
$\boldsymbol{n_g(a,t)}$	Annual net migration rate	Varies by age, sex and time	
$\boldsymbol{v_{cov}(t)}$	Infant vaccination (3-dose) coverage	Varies over time	World Health Organization [24]
**Parameters relating to treatment effect**			
$\boldsymbol{\alpha_T}$	Relative infectiousness of treated carriers compared to HBeAg-negative untreated carriers in horizontal transmission	1	Assumption based on viral suppression as a result of antiviral therapy
$\boldsymbol{m_{nT}}$	Relative risk for the probability of mother-to-child transmission from treated mother at birth compared to HBeAg-negative mothers without intervention	1	Assumption based on viral suppression as a result of antiviral therapy
$\boldsymbol{m^{TIe \to TH}}$	Hazard ratio for progression to HCC from Treated HBeAg-positive infection compartment compared to untreated HBeAg-positive infection	0.19	Chang, 2017 [59]
$\boldsymbol{m^{TCHB \to TH}}$	Hazard ratio for progression to HCC from Treated CHB compartment compared to untreated HBeAg-negative CHB	0.27	Nguyen, 2019 [60]
$\boldsymbol{m^{TC \to TH}}$	Hazard ratio for progression to HCC from Treated compensated cirrhosis compartment compared to untreated compensated cirrhosis	0.23	
$\boldsymbol{m^{TD \to TH}}$	Hazard ratio for progression to HCC from Treated decompensated cirrhosis compartment compared to untreated decompensated cirrhosis	0.23	
$\boldsymbol{\mu^{TD}}$	Annual HBV-related mortality from treated decompensated cirrhosis	0.18	Desalegn, 2019 [61]
**Parameters relating to implementation of screening and treatment**			
$\boldsymbol{T_{screen}}$	Year(s) of screening intervention	Varied in analysis	/
$\boldsymbol{A_{screen}}$	Age range covered by screening intervention	Varied in analysis	/
$\boldsymbol{p_{screen}}$	Screening coverage in the Gambian population in the targeted age range	Varied in analysis	/
$\boldsymbol{p_{assess}}$	Probability of diagnosed HBV carrier undergoing full clinical assessment for treatment eligibility	Varied in analysis	/
$\boldsymbol{p_{treat}}$	Probability of identified treatment-eligible carrier initiating therapy	Varied in analysis	/

Parameter	Description	Value	Justification and sources
p_{monit}	Probability of initially treatment-ineligible carriers completing monitoring assessment at each follow-up	Varied in analysis	/
$t_{monit}(a)$	Average interval between monitoring assessments (years)	Varied in analysis	/

CHB = chronic hepatitis B, HCC = hepatocellular carcinoma.

Demography

As chronic HBV infection is a long-lasting infection and many of the key events in the natural history are age-specific, the transmission model was developed to reproduce the population age structure and demographic processes of The Gambia. According to standard demographic theory, the population dynamics were modelled using time-, age- and sex-specific mortality, fertility and net migration rates. These were parameterised using country-specific estimates and projections from the United Nations World Population Prospects (UN WPP) 2017 for the 1950-2100 period [58] (**Table S2.6**). Every model compartment was assumed to experience the same background mortality and net migration. As cause-specific deaths due to HBV represent only a small proportion of all deaths in a population, the all-cause mortality rates from the UN WPP were not adjusted for HBV-related deaths. Calculation of births in the model is further detailed in the section on mother-to-child transmission.

The model was simulated from the year 1850, assuming the same demographic rates as from 1950-2050, and an endemic equilibrium was achieved after 100 years. The initial numbers in each compartment were based on UN WPP data of the 1950 population size by age in The Gambia, multiplied by estimates for the distribution across epidemiological compartments. In 1950, having fully replaced the initial population, the total population size was rescaled to correspond to the 1950 population size estimate from the UN WPP while maintaining the correct distribution across age and epidemiological compartments.

The demographic processes in the model against UN WPP estimates of the population size over time and by age are illustrated in **Figure S2.2** and **Figure S2.3**.

Table S2.6. Demographic parameters and data sources from UN World Population Prospects 2017. The medium variant of projected rates from 2020 to 2100 was used, where applicable.

Parameter	Description	Source
$\boldsymbol{\mu_g(a,t)}$	Age-, sex- and time-specific mortality rate	Extracted central death rate m(x,n) from abridged life tables datasets.
$\boldsymbol{f(a,t)}$	Age- and time-specific fertility rate	Extracted from age-specific fertility rates dataset.
$\boldsymbol{SP_g}$	Sex-specific proportion of births	Calculated from sex ratio at birth dataset.
$\boldsymbol{n_g(a,t)}$	Age-, sex- and time-specific net migration rates	Calculated from age-specific survival ratio S(x,n) in abridged life tables datasets and the age-specific population size in 5-year time periods using the forward survival method.

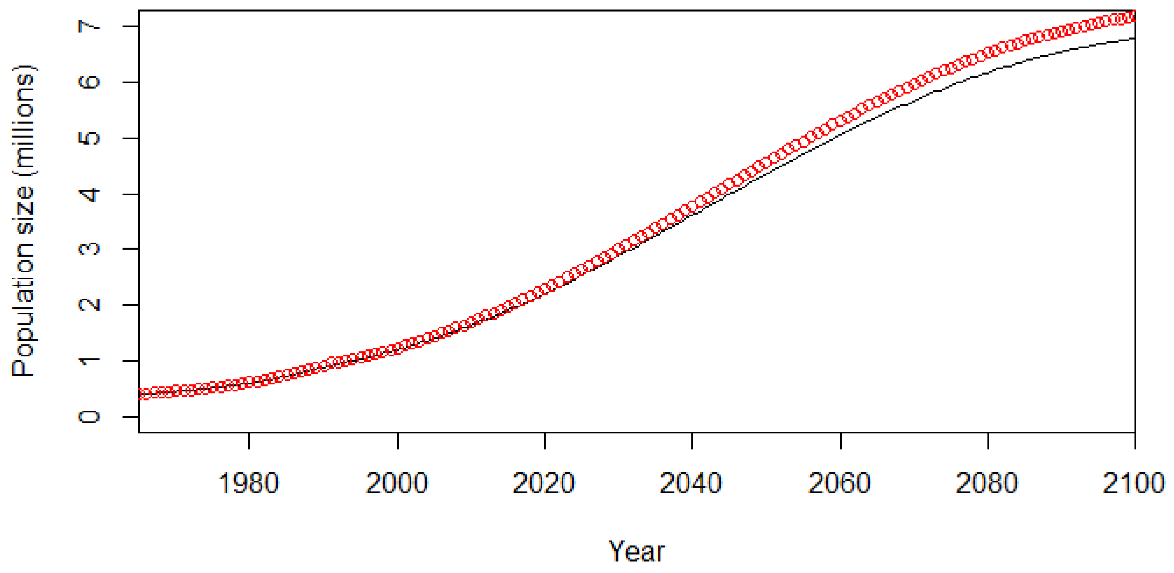

Figure S2.2. Total population size of The Gambia over time. The black line represents the modelled population size and the red dots show the data and medium variant forward projections from UN World Population Prospects.

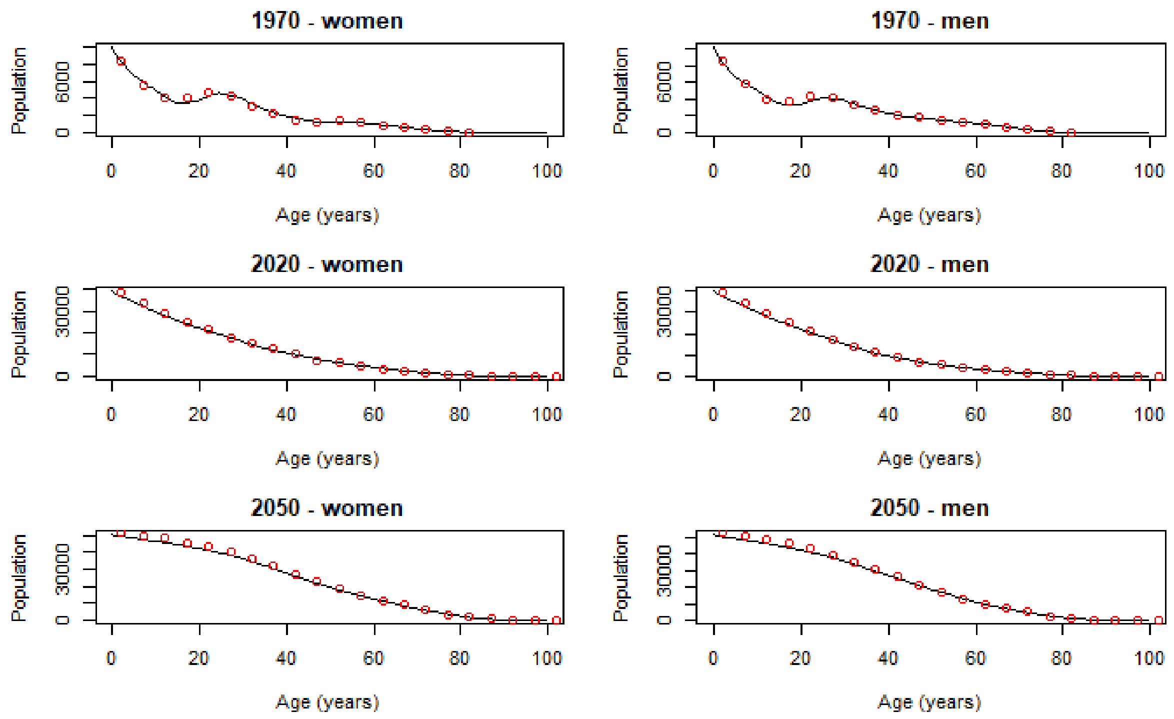

Figure S2.3. Age distribution of the Gambian female and male population at different timepoints. The black line represents the modelled population structure and the red dots show the data and medium variant forward projections from UN World Population Prospects.

Transmission

Two transmission routes from chronically infected to susceptible people are represented: horizontal transmission across the whole population with age-dependent mixing, and vertical transmission from mother to child, with infectivity of a carrier depending on their HBeAg serostatus. The transmission structure and prior ranges for the transmission parameters reflect pre-vaccination HBV epidemiology in sub-Saharan Africa, where the main source of new chronic infections was horizontal transmission in young children [3, 20, 62]. Following infection, progression to the chronic carrier states additionally depends on the age-specific risk of chronic carriage *p(a)*, which is highest in the youngest age groups [1].

Force of infection due to horizontal transmission

The rate of acquiring (acute) infection through horizontal transmission depends on age and follows a broad age structure, adapted from Nayagam *et al.* 2016 [3] and Edmunds *et al.* 1996 [20]. The force of infection $\lambda(a,t)$ was assumed to be constant within the four discrete age groups 0-0.5 years, 0.5-5 years, 5-15 years and 15-100 years. Differential mixing patterns among and between these age groups are represented using a Who acquires infection from whom (WAIFW) matrix (**Figure S2.4**). In this structure, the three β transmission coefficients specify the distinct rates of transmission between infectious carriers in age group *s* and susceptibles in age group *j*. These transmission coefficients can be understood to represent the per-capita effective contact rate per unit time, which depends on the frequency of contact within and between different age groups, as well as the probability of HBV transmission given this contact. For this study, a simplified version of the WAIFW matrix presented in the first published model of HBV transmission dynamics in The Gambia [20] was adopted since no new or conflicting evidence on HBV infection incidence or patterns of anti-HBc prevalence was identified in the scoping review.

Age group (years)		s			
		1	2	3	4
j	1	0	0	0	0
	2	0	β_1	β_2	0
	3	0	β_2	β_2	β_3
	4	0	0	β_3	β_3

Figure S2.4. The structure of the WAIFW matrix, β_{js}, for age-dependent contact. The age groups numbered 1 to 4 correspond to 0-0.5, 0.5-5, 5-15 and 15-100 years, respectively.

Since transmission is stratified into 4 discrete age groups indexed j = 1,…,4, we use notation $\lambda_j(t)$, which is the force of infection for infection among individuals in age group *j*.

$$\lambda_j(t) = \sum_{s=1}^{4} \beta_{js} \times \frac{HBeAg\text{-}negative\ carriers_s + \alpha\ \times HBeAg\text{-}positive\ carriers_s +\ \alpha_T\ \times Treated\ carriers_s}{N_s}$$

Where β_{js} corresponds to the symbols in **Figure S2.4**, N_s is the total population in age group s, α is the relative infectiousness of untreated HBeAg-positive compared to untreated HBeAg-negative carriers, α_T is the relative infectiousness of treated carriers compared to untreated HBeAg-negative carriers, and:

$$HBeAg\text{-}negative\ carriers_s = In_s(t) + Dn_s(t) + C_s(t) + D_s(t) + H_s(t) + SIn_s(t) + SDn_s(t) + SC_s(t) + SD_s(t) + SH_s(t)$$

$$HBeAg\text{-}positive\ carriers_s = Ie_s(t) + De_s(t) + SIe_s(t) + SDe_s(t)$$

$$Treated\ carriers_s = TIe_s(t) + TCHB_s(t) + TC_s(t) + TD_s(t) + TH_s(t)$$

The full compartment names can be found in **Table S2.3**. Based on knowledge of HBV transmission dynamics, the force of infection in the model depends on the proportion of infectious individuals and is therefore not influenced by the growth of the Gambian population over time [20].

Key assumptions in the transmission structure are that:

- infants younger than 6 months do not participate in horizontal transmission (they can only be infected at birth through MTCT, as described below).
- there is no horizontal transmission between adults (aged 15+ years) and young children (<5 years).
- susceptible young children (age 0.5-5 years) are horizontally infected from other children their age at a rate β_1 and from older children (age 5-15 years) at a rate β_2.
- susceptible older children (age 5-15 years) are horizontally infected from other children their age and younger children at a rate β_2, and from adults (age 15+ years) at a rate β_3.
- susceptible adults (age 15+ years) are horizontally infected from older children (age 5-15 years) and other adults at a rate β_3.

Priors for the β parameters were chosen so that $\beta_1 > \beta_2$ and $\beta_1 > \beta_3$.

For implementation of different prevention interventions, the transmission structure was designed to impose a clear distinction between horizontal and vertical transmission routes by allowing parent-to-child transmission through MTCT only. Conversely, the effective contact

rate among adults and between adults and older children reflects a range of possible transmission routes such as transmission within the household, sexual and iatrogenic transmission. These were not explicitly represented, as they were assumed to contribute comparatively little to the sub-Saharan transmission dynamics of chronic infection. Heterogeneity in behaviour e.g. in sexual interactions was therefore not included.

Births and mother-to-child transmission

Mother-to-child transmission is assumed to occur exactly at birth from HBsAg-positive mothers to some of their children, so babies are born either into the susceptible (compartment *S*) or the first chronic carrier stage (compartment *Ie*), dependent on reported risks of mother-to-child transmission by maternal HBeAg serostatus and the risk of becoming a chronic carrier following infection in newborns.

The boundary conditions for the differential equations describe the number of male and female births at each timestep, which were calculated by multiplying the age-specific fertility rates with the number of women of reproductive age (age 15-49 years) in each compartment, and applying the sex ratio at birth.

$$S_g(0,t) = SP_g \times \left[\int_{15}^{49} N_{g=f}(a,t) \times f(a,t)\, da - B_{inf}(t)\right]$$

$$Ie_g(0,t) = SP_g \times B_{inf}(t)$$

In this equation, $N_{g=f}(a,t)$ is the total female population of age *a* at time *t*, SP_g is the proportion of newborns of sex *g*, and $B_{inf}(t)$ is the number of chronically infected newborns at time *t*. This is calculated as:

$$\begin{aligned} B_{inf}(t) = p(0) \times \int_{15}^{49} [&\upsilon_e \times HBeAg\text{-}positive\ carrier\ mothers(a,t) \\ &+ \upsilon_n \times HBeAg\text{-}negative\ carrier\ mothers(a,t) \\ &+ \upsilon_T \times Treated\ carrier\ mothers(a,t)] \times f(a,t)\, da \end{aligned}$$

Where $p(0)$ is the risk of becoming a chronic carrier at age 0, υ is the probability of mother-to-child transmission from a HBsAg-positive mother depending on maternal HBeAg or treatment status (υ_e for HBeAg-positive mother, υ_n for HBeAg-negative mother and υ_T for treated mother), and:

$$\left.\begin{aligned}
&HBeAg\text{-}positive\ carrier\ mothers(a,t)\\
&\qquad = Ie_g(a,t) + De_g(a,t) + SIe_g(a,t) + SDe_g(a,t)\\
&HBeAg\text{-}negative\ carrier\ mothers(a,t)\\
&\qquad = In_g(a,t) + Dn_g(a,t) + C_g(a,t) + D_g(a,t) + H_g(a,t)\\
&\qquad + SIn_g(a,t) + SDn_g(a,t) + SC_g(a,t) + SD_g(a,t) + SH_g(a,t)\\
&Treated\ carrier\ mothers(a,t) = TIe_g(a,t) + TCHB_g(a,t) + TCHB_g(a,t)
\end{aligned}\right\} \quad g = f$$

The probability of mother-to-child transmission from a HBsAg-positive mother receiving antiviral therapy is calculated as follows:

$$v_T = m_{nT} v_n$$

Where m_{nT} is the relative risk for the probability of MTCT for pregnant women on treatment compared to HBeAg-negative mothers without intervention.

Note treatment here refers to antiviral therapy for prevention of individual disease progression and not peripartum antiviral prophylaxis for prevention of MTCT. Assumptions in this are further detailed in the *Simulated interventions: antiviral treatment* section.

Natural history of chronic HBV infection

The full model structure is shown in **Figure S2.5**. Chronic infection was stratified into 7 compartments corresponding to the clinical stages of chronic HBV infection according to the most recent guidelines from the European Association for the Study of the Liver (EASL) published in 2017 [22]. Classification into these stages is based on a combination of clinical measurements (HBeAg, viral load, ALT and extent of liver damage), while progressive liver disease is represented in the compensated cirrhosis, decompensated cirrhosis and HCC compartments.

Stratification based on the EASL clinical model was used in a previous global HBV modelling study [3] and was also considered the most suitable mechanistic approach for the purpose of this study, because:

- Results from the scoping review and more recent literature showed that the clinical risk factors for liver disease underlying the compartments have been confirmed in sub-Saharan African populations (e.g. HBeAg, high viral load, elevated ALT) [8, 63].
- Adoption of the EASL infection stages in the model structure allowed to easily apply the EASL HBV treatment criteria, which are currently the most commonly used reference criteria in sub-Saharan African studies [64].
- In the absence of an African-specific clinical model of HBV infection, the highest-quality data on disease progression to date is based on the EASL classification [8].

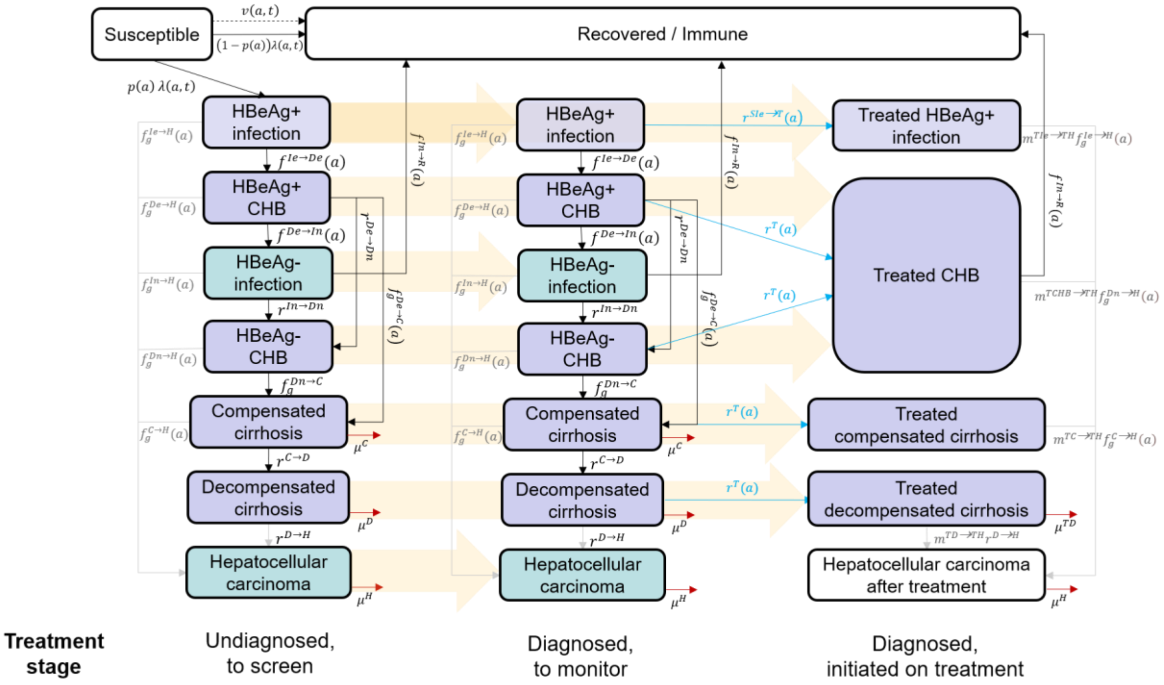

Figure S2.5. Diagram of full model structure. Note all compartments are further subdivided by age (*a*) and by sex (*g*). Births into the susceptible and undiagnosed HBeAg-positive infection compartments, and background mortality and migration rates from all compartments, are not shown. Red arrows indicate hepatitis B-related mortality from cirrhosis and hepatocellular carcinoma. Progression to hepatocellular carcinoma is expressed as grey arrows. Treatment-eligible compartments are coloured purple and treatment-ineligible compartments are coloured green. Yellow arrows show transitions to diagnosed or treated compartments as a result of the screening and treatment intervention, and blue arrows show transitions to treated compartments as a result of monitoring. CHB = chronic hepatitis B, HBeAg+ = hepatitis B e antigen positive, HBeAg- = hepatitis B e antigen negative. All parameter names and definitions are summarised in **Table S2.4** and **Table S2.5**.

To reduce model complexity within the scope of the research questions, several simplifying assumptions were introduced in the structure. Firstly, the infection and disease states represented by different compartments are not all distinct or mutually exclusive in reality. For example, a large proportion of HCC patients also have cirrhosis, whereas the model only distinguishes between non-malignant compensated or decompensated cirrhosis and HCC irrespective of cirrhotic status, which groups patients according to clinical management decisions. Secondly, the chronology in disease progression is simplified in the model, as reversion to previous stages (e.g. HBeAg seroreversion) and fluctuations in disease states (e.g. repeated ALT flares in CHB patients) can also occur. Thirdly, application of treatment criteria in the model does not reflect the fact that not all HBV carriers can be definitely assigned to one of the infection states at any one time, and that classification into these will usually require measuring the different clinical markers at several timepoints [8].

Age- and sex-specific natural history transitions

Age- or sex-dependent transitions between model compartments in the model are detailed below. Similar assumptions on disease progression rates have been applied in previous mathematical models [3, 20], and the mechanism behind many of these, including identification of age and sex as risk factors for disease progression, are well-established [65]. However, based on evidence from the scoping review, the age- and sex-dependence of some progression rates were adapted to the sub-Saharan African context. Equations use the following nomenclature: *r* represents a fixed rate (described by a single value), *f* represents a function (a rate depending on another factor, usually age *a* or sex *g*), *m* represents a multiplier (usually a rate ratio) and *c* represents a coefficient in function.

Age-dependent risk of becoming a chronic carrier after acute infection

A key determinant of different epidemiological patterns of HBV across the world is the age-specific risk of progressing to chronic carriage following acute infection [66], which is highest in the youngest age groups. As described in Edmunds *et al.* 1993 and Edmunds *et al*. 1996 [1, 20], the risk of developing chronic carriage was represented using an exponential decay function of age at infection for those aged 6 months and over, but was assumed to be higher for infants infected at less than 6 months of age through vertical transmission. This is represented by the following equation, where $p(a)$ is the age-specific probability of developing chronic infection following acute HBV infection, p_0 is the probability of developing chronic carriage following acute infection at birth, and c_r and c_s are coefficients of the exponential decay function estimated in the calibration procedure.

$$p(a) = \begin{cases} p_0 & a < 0.5\ years \\ \exp\left(-c_r \times a^{c_s}\right) & a \geq 0.5\ years \end{cases}$$

Age-dependent progressions through the HBeAg-positive compartments

The age-specific progression rates from the HBeAg-positive infection phase to HBeAg-positive CHB, $f^{Ie \to De}(a)$, and from HBeAg-positive CHB to HBeAg-negative infection $f^{De \to In}(a)$, were represented using an exponential growth function of age, as follows:

$$f^{Ie \to De}(a) = r^{Ie \to De} \times \exp\left(c_{eag} a\right)$$

$$f^{De \to In}(a) = r^{De \to In} \times \exp\left(c_{eag} a\right)$$

According to this formulation, $r^{Ie \to De}$ describes the progression rate from HBeAg-positive infection to HBeAg-positive CHB in 0-year olds, $r^{De \to In}$ describes the progression rate from HBeAg-positive CHB to HBeAg-negative infection in 0-year olds, and c_{eag} is the rate parameter of the function driving the variation with age.

This function was introduced to reflect the observations in a Gambian cohort of the crude rate of HBeAg loss increasing with age [8], and is consistent with previous evidence that the duration of the HBeAg-positive infection phase seems to be shorter in chronic infections acquired in adulthood compared to childhood [67, 68]. Nevertheless, the option of progression through the HBeAg-positive compartments remaining constant over age is also inferred in the calibration through the c_{eag} parameter, whose prior distribution includes 0.

Age-dependent rate of HBsAg loss

The age-specific rate of HBsAg seroclearance, $f^{In\rightarrow R}(a)$, corresponding to the transition from the HBeAg-negative infection to the Recovered/Immune compartment, was described as a linear function of increase with age, as informed by the c_{sag} slope coefficient:

$$f^{In\rightarrow R}(a) = c_{sag}a$$

This was based on analysis of age-specific HBsAg loss rates data from Shimakawa *et al.* [8], which showed a significant linear association between current age and the rate of HBsAg loss after adjusting for sex, calendar year and birthplace.

Age- and sex-dependent progression to compensated cirrhosis

A general feature of chronic HBV infection is HBeAg positivity early in the infection, which is usually lost as the infection progresses, giving rise to the age-specific pattern of HBeAg prevalence. However, if HBeAg persists, it was found to be a risk factor for progression to liver disease in older carrier cohorts [8]. In the model, the progression rate from HBeAg-positive CHB to compensated cirrhosis, $f_g^{De\rightarrow C}(a)$, was assumed to be constant with age above the age threshold, t_c, below which no development of cirrhosis occurs:

$$f_g^{De\rightarrow C}(a) = \begin{cases} 0 & a < t_c \; and \; g \in \{m, f\} \\ r^{De\rightarrow C} & a \geq t_c \; and \; g = f \\ r^{De\rightarrow C} \times m_{cirr} & a \geq t_c \; and \; g = m \end{cases}$$

This allows for the distinction of the effect of HBeAg positivity on disease progression based on duration of infection.

The higher risk of cirrhosis in men was represented through calibration of m_{cirr}, the rate ratio for progression from CHB to compensated cirrhosis in men compared to women. This parameter also governs the sex-dependent progression from HBeAg-negative CHB to compensated cirrhosis, $f_g^{Dn\rightarrow C}(a)$, by multiplication with the corresponding progression rate in women, $r^{Dn\rightarrow C}$:

$$f_g^{Dn \to C}(a) = \begin{cases} r^{Dn \to C} & g = f \\ r^{Dn \to C} \times m_{cirr} & g = m \end{cases}$$

Age- and sex-dependent progression to HCC

Progression to HCC in the model occurs at different rates from all chronic carrier compartments. An age threshold t_h, below which no progression to HCC occurs, was introduced to regulate age-specific progression to HCC as this outcome is rare in children. Progression to HCC strongly increases with age [49], therefore these progression rates from all chronic carrier compartments except for decompensated cirrhosis were described using the same shifted quadratic function:

$$f_g^{X \to H}(a) = \begin{cases} 0 & a < t_h \\ m^{X \to H} \times (c_{hcc}\,(a - t_h))^2 & a \geq t_h \text{ and } g = f \\ m^{X \to H} \times (c_{hcc}\,(a - t_h))^2 \times m_{hcc} & a \geq t_h \text{ and } g = m \end{cases}$$

$$X \in \{I_e, D_e, I_n, D_n, C\}$$

The baseline age-specific HCC incidence rate in women in the HBeAg-negative infection phase, c_{hcc}, was calibrated and rate ratios were applied to this for progression to HCC from the other compartments ($m^{X \to H}$ parameters) and from men in the respective compartments (m_{hcc} parameter).

HBV viral load has been established as an important risk factor for HCC in the Taiwanese REVEAL cohort, and underlying liver disease is known to increase the rate of progressing to HCC in chronic carriers [43]. For this reason, the rate of developing HCC was assumed to be lowest in HBeAg-negative infection, followed by HBeAg-positive infection, HBeAg-negative CHB, HBeAg-positive CHB and compensated cirrhosis. The rate of progression to HCC from decompensated cirrhosis is assumed to be constant with age.

Assumptions on the effect of sex in natural history

The increased risk of developing liver disease, including HCC, in men compared to women, was reflected in several progression rates in the model [3, 43]. As shown above, a multiplier for men was applied to the rates of progression to compensated cirrhosis from HBeAg-positive and HBeAg-negative CHB (m_{cirr}) and to HCC from all chronic carrier compartments except for decompensated cirrhosis (m_{hcc}). The rate ratio was assumed to be potentially different for progression to compensated cirrhosis and for progression to HCC, due to the distinction between the liver disease pathway and the viral DNA integration pathway to HCC and the hypotheses for the reasons underlying the sex differences [30].

Assumptions on other risk factors for disease progression relevant to the sub-Saharan African context

Several environmental, genetic or lifestyle risk factors for increased risk of disease among chronic HBV carriers have been described or hypothesised, for example coinfections with HIV, hepatitis delta or hepatitis C virus, aflatoxin exposure and HBV genotype [6, 8, 69, 70]. However, since the purpose of this study was to represent the general chronic HBV carrier population in The Gambia, as a case study of chronic carriers in sub-Saharan Africa more generally, these risk groups were not explicitly represented in the model structure. Instead, progression rates calibrated based largely on West African data implicitly include the prevalence and distribution of these risk factors in the study population at the time of data collection. This focus on West African data may affect generalisability of the results to other African countries, as distribution of some risk factors varies across the region.

Implemented interventions: infant vaccination

Routine infant vaccination was modelled as conferring all-or-nothing lifelong immunity from chronic HBV infection by removing infants from the susceptible compartment at a rate $v(a,t)$.

The age- and time-dependent annual vaccination rate $v(a,t)$ is defined as:

$$v(a,t) = \begin{cases} 0 & t < 1990 \; and \; a \; \geq 1 \; years \\ \dfrac{-\ln\left(1 - v_{eff} \times v_{cov}(t)\right)}{0.5} & t \; \geq 1990 \;\; and \; a < 1 \; years \end{cases}$$

Where v_{eff} is the vaccine efficacy against chronic infection and $v_{cov}(t)$ is the time-specific 3-dose infant vaccination coverage, which equals 0 before the year of vaccine introduction (1990). The rate of vaccination was parameterised using yearly historical 3-dose vaccine coverage data from the WHO [24], with missing estimates being interpolated over time, and the calibrated efficacy of infant vaccination against chronic infection, with prior ranges based on empirical data from the literature.

The hepatitis B vaccine is incorporated in routine vaccination schedules as 3 doses at 2, 3 and 4 months within the Expanded Programme on immunizations [23]. In the model, routine vaccination occurs by applying the annual vaccination rate to children in the first year of life, whereby they are transferred to the Immune compartment and receive the full benefits of vaccination. This assumes that vaccination with less than 3 doses confers no immunity. A logarithmic adjustment was applied to convert the effective vaccine coverage into a transition rate [71], accounting for the observation that the vaccination schedule is usually completed within the first 6 months of life in The Gambia [28]. Given high estimates of vaccine efficacy,

the proportion of 1-year old children immune to HBV as a result of vaccination in the model was confirmed to be almost as high as the observed WHO coverage data at 1 year of age.

Simulated interventions: antiviral treatment

Treatment regimens and eligibility

The treatment regimen implemented in the model consists of first-line oral antiviral nucleos(t)ide analogues, in particular tenofovir disoproxil fumarate. Both entecavir and TDF are widely used worldwide due to their high efficacy, favourable safety profile, and ease of administration and patient management compared to older treatment regimens like lamivudine and interferon [72]. Entecavir has been shown to have very similar efficacy to TDF [73] and both drugs are off patent [74]. Nevertheless, as TDF is already used in most of Africa in HIV treatment regimens [75], this was considered to be the most likely HBV antiviral therapy to be scalable in the sub-Saharan African context.

The recommended treatment duration with TDF is indefinite in nearly all patients, therefore lifelong antiviral therapy was assumed in the model. Due to the excellent safety profile of TDF and high barrier to resistance [22], potential side effects of treatment or treatment failure as a result of drug resistance were not accounted for.

According to EASL 2017 guidelines, carriers in the HBeAg-positive CHB, HBeAg-negative CHB, compensated and decompensated cirrhosis states, as well as carriers over the age of 30 years in the HBeAg-positive infection phase, were eligible for treatment [22]. Carriers in the HBeAg-negative infection phase usually have a very good prognosis and minimal liver damage, and are therefore not considered in need of antiviral treatment. EASL treatment criteria were chosen over those from WHO because they are currently the most common reference criteria used in published African treatment studies, and are in agreement with the clinical guidelines developed by other international liver associations [15, 64, 76]. Evidence from studies of treatment eligibility in sub-Saharan African populations also suggests that the WHO guidelines, developed specifically for use in resource-limited settings, fail to identify a substantial proportion of patients in need of treatment from a clinical perspective, especially those who would benefit most from a timely initiation to prevent advanced disease [76].

Development of the treatment model structure

The treatment model structure for treatment-naïve HBV carriers was developed and parameterised based on the most recent international evidence on disease progression on antiviral therapy, as only little data was available from a sub-Saharan African setting at the time of development.

Long-term treatment with nucleos(t)ide analogues is highly effective in suppressing HBV viral load, normalising biochemical response, and halting or reversing the progression of liver damage and cirrhosis, but does not prevent the development of HCC [72]. Therefore, disease progression on treatment was captured within 6 compartments, corresponding to 4 different treated disease stages and 2 possible long-term outcomes on treatment (**Figure S2.5**). Movement into the treated compartments represents effective viral suppression, which has been widely reported to occur in almost all patients within a short time on treatment [72, 77], including in sub-Saharan African populations [15, 75, 78]. For simplicity, we therefore assumed viral suppression occurs in all patients initiating treatment within one timestep of 6 months in the model. This means that treatment has an immediate effect on liver disease progression in all patients upon initiation.

Assumptions on outcomes of treated carriers

Outcomes on treatment in the model vary by the disease state at treatment initiation. Carriers aged over 30 years in the HBeAg-positive infection compartment transition into the Treated HBeAg-positive infection compartment, and carriers in the (non-cirrhotic) CHB compartments, irrespective of HBeAg status, transition to the Treated CHB compartment. An implicit assumption of the latter is that HBeAg loss occurs among HBeAg-positive CHB carriers once they initiate therapy, though in reality this occurs in only a subset of patients [77, 79]. Carriers with compensated and decompensated cirrhosis move to the corresponding treated compensated and treated decompensated cirrhosis compartments.

In addition to histological improvement and preventing the progression to cirrhosis, studies have shown that antiviral therapy can lead to regression of cirrhosis in the majority of patients [80]. However, the implications of this for future disease progression to HCC are uncertain. In the model, we therefore assumed no disease regression to other compartments on treatment, though reduced HCC risk experienced by the treated persons may in part be due to histological improvement [60]. Instead, antiviral therapy was assumed to be 100% effective at halting progression to cirrhosis if it is initiated before the onset of cirrhosis, as well as no further decompensation of cirrhosis on treatment [81]. We also assumed a reduction in cirrhosis mortality rates on treatment to reflect the slowed progression of liver disease and an improvement in complications in decompensated cirrhosis patients [22].

Due to viral integration in the host liver cell genome and persistence of covalently closed circular DNA, a reduced risk of progression to HCC remains even while on treatment, which is higher in patients with underlying cirrhosis at treatment initiation [82-84]. The age- and sex-dependency in progression rates to HCC also appears to be maintained on treatment [60, 83]. In the treatment model, the calibrated untreated progression rates from all compartments to

HCC were reduced according to the hazard ratios from the international literature, thereby assuming that the effect of treatment is the same in sub-Saharan African carriers in a given infection or disease state as estimated in studies in other populations, and also allowing to maintain the increased risk of HCC in cirrhotic compared to non-cirrhotic carriers. Though little evidence on varying effects of first-line treatment regimens by genotype or ethnicity is available to date, a comparison of Asian and non-Asian (predominantly Caucasians with genotypes A or D) participants in TDF trials found similar levels of viral suppression, ALT normalisation and histological improvements achieved in these two groups [85].

Based on a retrospective cohort study in North American and Taiwanese patients [60], we assumed a fixed reduction of progression to HCC with treatment of 73% for CHB and 77% for cirrhotic compartments. This study was chosen based on treatment regimen, long-term follow-up, and incidence rates being in line with those reported in other large studies [84]. However, estimates of hazard ratios vary across studies to some degree, particularly regarding differential treatment effect by cirrhotic status [86], which was therefore further explored in sensitivity analyses.

As the treated HCC compartment represents development of HCC following treatment and other therapeutic options for management of HCC such as liver transplants are not widely available in sub-Saharan Africa, we assumed no change in the mortality rate from HCC with treatment.

Treatment of carriers in the HBeAg-positive chronic infection phase if aged over 30 years is a new conditional recommendation in the 2017 version of the EASL guidelines and accordingly used in the current treatment of HBV carriers in ongoing studies [87]. However, evidence on disease progression following treatment in these patients is lacking. Although a Korean study suggested a similar reduction in progression to HCC in treated HBeAg-positive infection as we assumed for CHB (adjusted hazard ratio of 0.19, 95% CI 0.05-0.69) [59], treatment need in these patients remains controversial [88, 89]. On the population level, this was thought not to have a substantial effect on projections of treatment impact due to the small number of HBV carriers remaining in this phase over the age of 30 years in sub-Saharan Africa [8].

Serological recovery through HBsAg seroclearance is considered the ideal end point of antiviral therapy, but represents a rare outcome. In a recent systematic review and meta-analysis, rates of HBsAg seroclearance were estimated to be similar on treatment as in untreated carriers, and not associated with genotype [90]. As a result, the same age-specific rate of HBsAg loss as in untreated carriers was applied to the treated CHB compartment, which was also consistent with new data from a treated cohort in Ethiopia [61].

Assumptions on treatment adherence and duration

We modelled all patients initiating treatment to remain on the recommended life-long antiviral therapy course, expect for treated CHB carriers experiencing HBsAg loss. Imperfect adherence or cessation of therapy was not explicitly accounted for in the model. Though lifelong perfect adherence to treatment is unlikely to be realistic in practice, evidence on disease progression after stopping of nucleos(t)ide analogue therapy is inconclusive and potential options for discontinuation are an active area of ongoing research. Discontinuation is considered safe and recommended in the small proportions of patients achieving HBsAg loss, and can be considered as an individualised option in HBeAg-positive non-cirrhotic patients after HBeAg seroconversion and consolidation therapy [22, 91]. In recent years, sustained virological suppression has been observed in HBeAg-negative patients without cirrhosis following discontinuation after long-term treatment, although these patients still require close post-treatment monitoring to detect relapse [92, 93]. Interestingly, these results also suggest that stopping of nucleos(t)ide analogue therapy may increase the chance of HBsAg seroclearance. Nevertheless, in many other patients, treatment discontinuation appears to lead to ALT elevations within a short time [94], as well as increased disease progression such as to liver decompensation [95].

Conversely, current clinical trials have only followed treated patients for a maximum of around 10 years, so longer-term effects of treatment are also not known. Recent evidence suggests that the rate of progression to HCC declines with a longer treatment course in cirrhotic patients [84], so any potential longer-term benefits of lifelong therapy may not be reflected in the current data and thereby the parameter values in the model. Similarly, though imperfect adherence to treatment was not modelled explicitly, it may be implicitly represented in the parameters governing treatment efficacy, as the progression rates to HCC on treatment represent the average progression of a cohort treated and followed-up for a study period of 8 years. The treatment initiation uptake parameter can also either be interpreted as individuals not initiating treatment or not adhering to treatment enough for a beneficial effect on disease progression to occur.

Assumptions on infectivity of treated carriers

Reductions in infectivity of HBV carriers due to treatment are difficult to measure. Since nucleos(t)ide analogue therapy effectively suppresses HBV replication to undetectable levels, comparable to those in over half of treatment-ineligible inactive carriers in the HBeAg-negative chronic infection compartment [15], we assumed all treated carriers irrespective of HBeAg status at treatment initiation to be as infectious as HBeAg-negative untreated carriers in the model. We assumed this relative infectiousness for both horizontal transmission (governed by

the α_T parameter) and for treatment among pregnant women and their risk of MTCT (m_{nT} parameter). In the modelled scenarios, the latter applies to pregnant women receiving long-term antiviral therapy for prevention of individual disease progression and does not represent the MTCT risk for women receiving peripartum antiviral prophylaxis for the purpose of preventing MTCT.

Indirect evidence from a meta-analysis of prevention of MTCT with antiviral treatment during pregnancy appears to confirm that treatment has a significant impact on reducing infectivity, at least in the highly viraemic mothers who would be eligible for peripartum antiviral prophylaxis [96]. Though peripartum nucleos(t)ide analogue therapy is offered in conjunction with birth dose vaccination and HBIG in all studies, rates of failure to prevent MTCT were estimated at around 23% if only the birth dose vaccine and HBIG were given, but significantly lower at 6-11% if pregnant women additionally received peripartum antiviral prophylaxis. This is also consistent with their estimate of a 78% reduction in maternal viral load with peripartum antiviral prophylaxis [96]. Nevertheless, given the large uncertainty in how this might translate to infectivity in a treated population, a sensitivity analysis assuming no possibility of transmission from treated HBV carriers was also conducted.

Modelling of the mass screening and treatment intervention and the treatment cascade

A simplified cascade of care for clinical management of HBV was implemented in the model. In the modelled intervention, two potential routes to antiviral therapy are represented according to international guidelines: a) the initial mass screening intervention using HBsAg testing and clinical assessment to identify chronic HBV carriers in need of treatment in the population, and b) regular clinical monitoring of those carriers engaged in care who were found to be not eligible for treatment at the initial assessment.

The treatment cascade is described by parameters for the screening coverage in the targeted population, p_{screen}, the proportion of diagnosed HBV carriers undergoing full clinical assessment for treatment eligibility, p_{assess}, the proportion of HBV carriers identified as treatment-eligible who initiate antiviral therapy, p_{treat}, and the proportion of HBV carriers identified as treatment-ineligible at initial assessment who complete the monitoring assessment at each follow-up, p_{monit} (**Figure S2.6**).

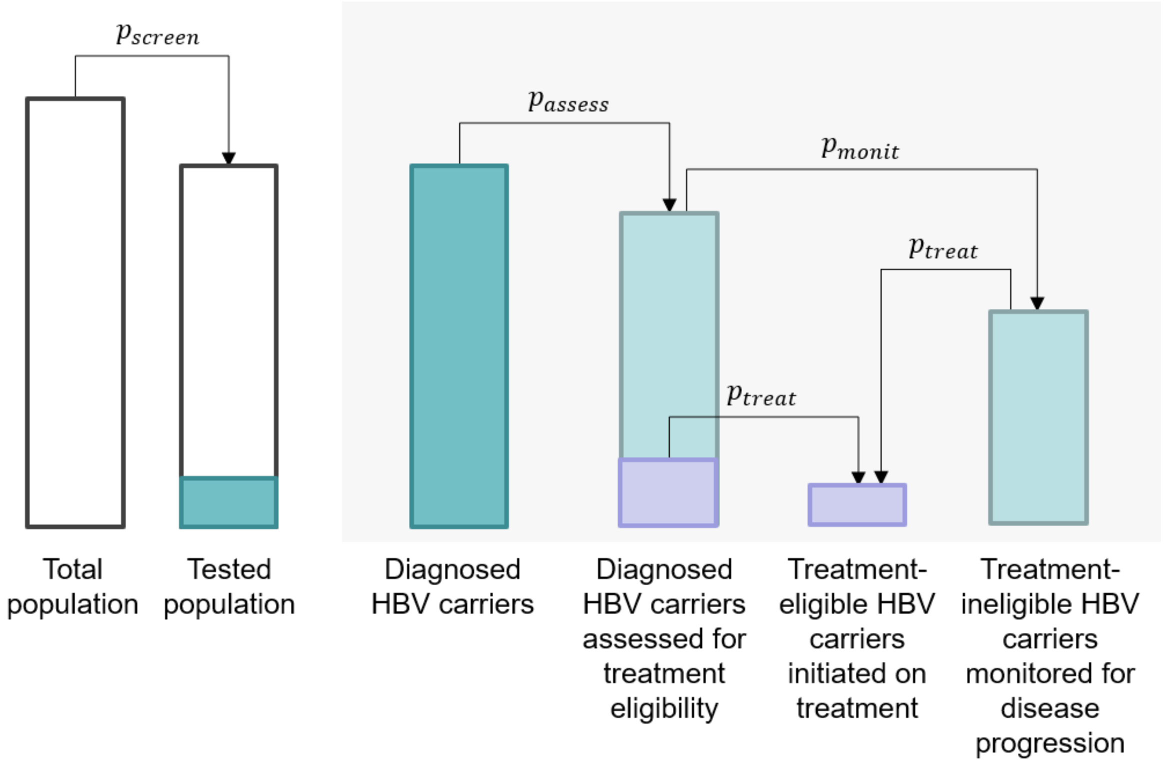

Figure S2.6. Diagram of the simplified treatment cascade for hepatitis B. The cascade is represented using model parameters for screening coverage (p_{screen}), clinical assessment uptake (p_{assess}), treatment uptake (p_{treat}) and monitoring uptake (p_{monit}). Diagnosed HBV carriers are a subset of the total tested population.

Mass screening and treatment programme

Hypothetical screening and treatment in the general population was modelled as a mass intervention programme which instantaneously moves a given proportion of the targeted population into the Diagnosed (untreated) and Treated compartments. Specifically, upon HBsAg testing, a proportion of undiagnosed treatment-eligible HBV carriers are moved to the corresponding treated compartment, and a proportion of undiagnosed treatment-ineligible carriers are moved to the corresponding diagnosed compartment (section *Equations for the mass screening and treatment programme*). This approach assumes that screening, clinical assessment and treatment initiation of the targeted population occurs within a short time interval of 6 months. In the code, it was implemented by triggering an "event" in the *deSolve* package that instantaneously changes the values of the respective state variables. Screening coverage is applied randomly to the targeted population, such as the general population falling into a given age range.

Monitoring of HBV carriers ineligible for treatment at initial assessment

European and other international guidelines recommend regular follow-up of at least once a year of HBV carriers not meeting treatment criteria at first assessment [22, 65, 97]. Monitoring

for disease progression to treatment eligibility among the diagnosed initially treatment-ineligible carriers is modelled as a continuous process, so that monitoring in treatment-eligible compartments leads to treatment initiation into the corresponding treated compartment at a constant rate $r^T(a)$:

$$r^T(a) = \frac{1}{t_{monit}(a)} \times p_{monit} \times p_{treat}$$

In this equation, $t_{monit}(a)$ is the average time interval between monitoring assessments, which is varied in scenario-based analyses and can vary by age. p_{monit} is the probability of monitoring uptake and p_{treat} is the probability of identified treatment-eligible carriers initiating therapy. The treatment rate among those being monitoring, r^T, does not depend on sex and is equally applied to the diagnosed CHB, cirrhosis and decompensated cirrhosis compartments.

For diagnosed carriers in the HBeAg-positive infection state, the rate of treatment among those being monitored, $r^{SIe \to T}(a)$, is only applied to over-30 year olds according to treatment criteria [22].

$$r^{SIe \to T}(a) = \begin{cases} 0 & a \le 30\,years \\ \frac{1}{t_{monit}(a)} \times p_{monit} \times p_{treat} & a > 30\,years \end{cases}$$

Since monitoring occurs at a constant rate in the model, an average monitoring interval ($t_{monit}(a)$) of e.g. 5 years translates to 20% of the population in the given compartment being monitored and initiated on treatment each year, and everyone having been monitored once after 5 years, twice after 10 years, etc. The treatment rate is reduced if not all carriers attend the monitoring assessments or initiate treatment where necessary (p_{monit} or $p_{treat} < 1$).

Further assumptions about the treatment cascade

The equations in previous sections only describe movements between compartments. However, to calculate the total population to screen, assess for treatment eligibility and initiate on treatment as a model outcome, the respective coverage parameters were applied to the whole population, all diagnosed chronic carriers and all identified treatment-eligible carriers, respectively. Similarly, monitoring events were counted among all diagnosed compartments.

All parameters describing the cascade of care were assumed not to vary based on disease status, age or sex. All tests and clinical assessments were assumed to have perfect sensitivity and specificity in diagnosing chronic HBV infection and identifying the clinical stage of disease governing treatment eligibility.

Discussion of influence of assumptions in treatment model on results

Conclusions about cost-effectiveness could be affected by the model assumption of perfect lifelong adherence to antiviral therapy. The feasibility of this is a key consideration for the projections of high treatment impact in young carriers, as the effect of including 15-30 year olds in a screening programme now compared to screening the same cohort at a later time was not compared. Globally, adherence to antiviral therapy for HBV was estimated at 75% [98], but evidence on the impact of treatment discontinuation on disease outcomes was considered too inconclusive to allow exploration in the model at present [91].

Other structural assumptions in the treatment model are likely to affect conclusions about the treatment programme, for example that the benefit of treatment does not vary by age independent of disease state or over time, that treatment leads to immediate viral suppression and that treated carriers experience a constant rate of developing HCC over their lifetime.

Given the large benefits associated with the initial assessment alone, lack of retention of treatment-ineligible carriers in monitoring did not affect the cost-effectiveness of a treatment programme without monitoring as it also saves resources for assessing those individuals lost to follow-up over time (see *Sensitivity analysis of cost-effectiveness results*). However, drop-out of carriers to monitor over time was not modelled explicitly, but was represented as an average 80% attendance at each monitoring visit. If in reality the drop-out rate was high early on, the model may overestimate identification of disease progression by monitoring and thereby its impact. We also did not explore the option of potentially differential drop-out rates with longer versus shorter monitoring intervals, which could make more frequent monitoring frequencies more cost-effective. However, it is noteworthy that given the modelled age distribution of chronic carriers, a monitoring strategy of every 5 years in 15-45 year olds would involve an average of less than two follow-up visits per person in total.

C. Model calibration

Approach

The model was calibrated in a Bayesian framework to synthesise epidemiological evidence from many different sources, and to allow quantification and propagation of uncertainty in input parameters. Results from the scoping review informed the choice of priors, calibration targets and which parameters to vary in the calibration.

A rejection-sampling Approximate Bayesian computation (ABC) algorithm was used for the calibration [99]. 1 million Latin Hypercube samples were drawn from the defined prior parameter space. The model outputs simulated from these parameter sets were compared with the empirical epidemiological data using a distance function, and the sample of parameter sets with a distance falling below a defined tolerance level were accepted.

Prior distributions

All parameters of the model representing the current epidemiology in The Gambia (without treatment) were varied in the calibration procedure across a range of prior probabilities (**Table S2.4**). This choice was made because most of the available sub-Saharan African data informed modelled outcomes that are functions of several parameters, instead of allowing to update individual parameters directly.

A total of 21 datapoints from 11 studies were identified for parameterisation, only four of which were longitudinal measurements matching natural history transitions or HBV-related mortality in the model (not shown). For most parameters, for which no data from sub-Saharan Africa was identified, priors were informed by evidence from other geographical areas and expert opinion, but were specified to be less informative by increasing the spread, both to account for the general uncertainty in the measurements as well as their applicability in sub-Saharan African populations. The most informative prior distributions were specified for well-established parameters such as infant vaccine efficacy and the rate ratios for progression to cirrhosis and HCC in men compared to women (**Table S2.4**). The centre and range of prior distributions was derived from the data sources reviewed for a previous modelling study [36] and more recent systematic reviews. Where available, multiple sources were considered to determine averages and ranges of the prior distributions.

Prior distributions for the individual inputs in composite parameters (usually of the age- and sex- dependent transition rates) that are not observed directly were derived by adjusting empirical measurements from studies set in specific populations using the progression functions described above.

ABC algorithm

The steps in rejection-sampling ABC [100], with the aim of minimising the error between simulated and observed data, are:

1. Sample a set of parameters θ^* from their prior distribution $p(\theta)$.
2. Generate the simulated data s^* from the model.
3. Compare the simulated output, s^*, with the observed data, s_{obs}, using a distance function d and tolerance level ϵ.
4. Accept the parameter set θ^* if $d(s^*, s_{obs}) \leq \epsilon$, otherwise reject.
5. Return to step 1.

Thereby, the rejection algorithm rejects all but the *N* parameter values that generate model outputs closest to the calibration targets, which form a sample from an approximation of the posterior distribution [101]. The algorithm was stopped after having sampled the pre-specified 1 million prior parameter sets.

To compute the observed outcomes, which depend on clinical and demographic population characteristics, populations similar to the respective study populations were simulated, and measured outcomes that were not explicitly represented in the model structure were approximated. Full details of how empirical observations were linked to pathways in the model are detailed in **Table S2.7**.

The distance between individual observed datapoints and corresponding model outputs was summarised in a single summary error value across all calibration targets, $d(s, s_{obs})$. The weighted sum of relative squares (SSE) was chosen as the union metric and calculated as:

$$d(s, s_{obs}) = \sum_{i=1}^{m} w_i \left(\frac{s_{obs,i} - s_i}{\frac{(s_{obs,i} + s_i)}{2}} \right)^2$$

where *i* refers to the individual calibration datapoints, with s_i being the simulated value and $s_{obs,i}$ the empirical value, *m* is the total number of calibration targets, and w_i is the assigned datapoint-specific quality-based weight.

This distance metric was chosen to reflect key properties of the dataset in question, namely the varying scales of the different datapoints, and the existence of potential (unknown) contradictions and outliers. It allowed to prioritise achieving a good overall fit over capturing all patterns in the data equally well [102], and allowed to make use of all the available data of varying quality. The evidence on HBV natural history processes embedded in the model structure, in combination with quality-based weights on the calibration targets, determined the

prioritisation of some datapoints over others. Weights were assigned based on quality scores reflecting subjective confidence in the different data sources, as further detailed in the *Data sources used to inform model parameters through calibration* section.

The choice of tolerance level aims to achieve a balance between accuracy of the posterior approximation and a computationally feasible acceptance rate [103]. For the dataset in question, where the decision of how close the simulated data should be to the observed data was not straightforward, we used a two-step approach to determine an appropriate tolerance level. Firstly, a broad target range was applied to the final simulations of HBsAg prevalence to ensure a realistic endemicity level among all accepted simulations. Parameter sets were sorted by their SSE and a cut-off applied to discard all simulations with a HBsAg prevalence outside the 1.3-41% range in over-20-year-olds, based on the minimum and maximum confidence interval bounds in the calibration dataset. Secondly, for the remaining 9,808 lowest-SSE parameter sets, we adopted the approach described in a previous study whereby the final tolerance level is chosen so as to maximise the precision in approximate posterior estimates [104]. K-means clustering was applied to the previously accepted parameter sets to find the tolerance level giving the smallest interquartile range across all posterior parameter values. This led to the final selection of the 183 lowest-SSE parameter sets, corresponding to a tolerance level of ϵ=76.25 and an acceptance rate of around 0.02%.

Propagation of uncertainty

Forward projections were made using the 183 accepted parameter sets to propagate the uncertainty in calibrated parameter values to the outcomes of interest in the analysis. Uncertainty in model outputs was quantified by reporting the median and 95% equal-tailed credible interval (CrI) (2.5^{th} and 97.5^{th} percentiles) of projections. Note that the uncertainty around projections only represents the uncertainty in epidemiological parameters relating to transmission, vaccination and untreated natural history. Uncertainty bounds on modelled outcomes do not account for the uncertainty in demographic projections or the effect of treatment on disease progression.

Data sources used to inform model parameters through calibration

The model was calibrated to 345 epidemiological datapoints from 38 papers, shown in Table S1. This included primary data and modelled estimates derived from systematic reviews and modelling studies, identified in the first stage of the scoping review. The primary data used as calibration targets broadly fall into the following categories: seromarker prevalence, transmission, disease progression in chronic HBV carriers, disease progression in liver disease patients, risk factors for liver disease in chronic HBV carriers, cross-sectional

characteristics of chronic HBV carriers, and cross-sectional characteristics of liver disease patients. Assumptions made in inclusion, processing and modelling of the different types of extracted calibration data are detailed in **Table S2.7**.

The weighting scheme was adapted empirically over several rounds of calibration. Previous attempts at a more neutral approach giving equal or similar weights across datapoints led to overrepresentation of late-stage disease among calibration targets. This is not representative of disease progression among chronic carriers on average, as many hospital-based studies reflect patient characteristics at presentation rather than at onset. This led to acknowledgement of the low to moderate quality of the majority of the datapoints in subsequent approaches by numerically differentiating calibration targets more strongly based on data quality, and by assigning a low weight to most targets. The default weight was 0.1, which was increased to 1 only for data from good-quality studies representative of the general population. An additional purpose of the weighting scheme was to reconcile conflicts in data on the same outcome from multiple sources. In these cases, the calibration targets were differentiated by up-weighting only the highest-quality studies. For seromarker prevalence studies, quality was assessed based on a combination of sample size, representativeness of the study population, geographic scope and quality of information on the age group under study. Additionally, only individual calibration targets on cross-sectional characteristics of chronic HBV carriers, the age-specific risk of developing chronic infection, and MTCT risk were upweighted. In general, low weights were assigned based on methodological issues in the original study (most commonly very small sample sizes or bias in ascertainment of liver disease, as informed by expert opinion), or difficulty in approximating the outcome in the model. Priority was given to datapoints describing the general population of chronic HBV carriers.

Table S2.7. Assumptions made in the use of calibration data.

Type of data	Details and assumptions	Use in model
**Seromarker prevalence**	The model was calibrated to three population-based measures of relevant seromarkers in The Gambia: HBsAg prevalence, representing current chronic infection, anti-HBc prevalence, representing exposure to HBV or the proportion of the population who has ever been infected (either current infection or serological recovery), and HBeAg prevalence in chronic HBV carriers, representing increased infectivity. Only anti-HBc prevalence data from the pre-vaccination period was included as (anti-HBc-negative) vaccine-induced immunity was not distinguished from (anti-HBc-positive) infection-induced immunity in the model structure.<br><br>HBsAg seroprevalence data was not searched for systematically, but was extracted from the studies included for other reasons and complemented by data from studies in a WHO systematic review [5, 105]. HBsAg prevalence was included if it was deemed representative of national HBsAg prevalence in The Gambian population; prevalence in study participants sampled based on their vaccination status in the Gambia Hepatitis Intervention Study or the Keneba-Manduar vaccination pilot was excluded. Vaccine effects on prevalence observed in the Keneba-Manduar trial was extrapolated to the national level by adjusting the time since vaccine introduction (from 1984 to 1990).	Seromarker prevalence was calibrated by stratifying by age and sex where relevant. The model output was calculated in the age group matching the average age of the respective study population, using the following equations:<br><br>$\text{HBsAg prevalence in the general population} = \frac{\text{Chronic carriers}}{\text{Total population in the model}}$<br><br>$\text{Anti}-\text{HBc prevalence in the general population} = \frac{\text{Ever infected individuals}}{\text{Total population in the model}}$<br><br>$\text{HBeAg prevalence in chronic HBV carriers} = \frac{\text{Chronic carriers in HBeAg}-\text{positive compartments}}{\text{Chronic carriers}}$<br><br>Where:<br>Chronic carriers = compartments *Ie*, *De*, *In*, *Dn*, *C*, *D* and *H*<br>Ever infected individuals = compartments *Ie*, *De*, *In*, *Dn*, *C*, *D*, *H* and *R*<br>Chronic carriers in HBeAg-positive compartments = compartments *Ie* and *De*
**Transmission: HBV infection incidence**	Data on the incidence of HBV infection in the population was only included from studies set in The Gambia, as this depends on background endemicity levels. Infection incidence in children by maternal HBsAg status was only included if stratified according to age younger or older than 1 year, to allow differentiation between MTCT and horizontal infection. In young age groups, progression from seromarker-negative status to anti-HBs positivity was interpreted to represent acquisition of acute infection followed by serological recovery.	Rates of infection with HBV in the population were calculated as described for disease progression rates (see below).
**Transmission: Mother-to-child**	Estimates of the risk of vertical transmission with unknown maternal HBeAg status were included as calibration targets, if the ascertainment of	Data of overall MTCT risk were calibrated by calculating the proportion of all babies born to HBsAg-positive

Type of data	Details and assumptions	Use in model
**transmission risk in HBsAg-positive women**	MTCT met the definition of HBV infection detected through HBsAg or HBV DNA within the first 3-12 months of life of an infant born to an HBsAg-positive mother. As in the systematic review informing the priors on MTCT risk by maternal HBeAg status [2], it was assumed that all HBV infections in under 1 year olds born to HBsAg-positive mothers are due to MTCT, and all new infections occurring after the first year of life representing horizontal transmission events.	mothers who are born as a chronic HBV carrier (born into the HBeAg-positive infection compartment) in the year of data collection of the given study. The overall MTCT risk was averaged between births from HBeAg-positive and HBeAg-negative women of childbearing age.
**Transmission: Age-specific risk of becoming a chronic carrier following acute HBV infection**	Data sources on the age-specific risk of developing chronic infection were derived from Edmunds *et al*. 1993 [1], as no additional studies on this were identified in the scoping review. However, as the original study included data from different world regions, the function of chronic carriage risk was fitted only to the West African datapoints of the proportion of children having progressed to chronic carriage following acute infection at various ages at infection.<br><br>In extracting the data, the methodology described in Edmunds *et al*. 1993 was applied. The age at infection in a study, if not reported, was assumed to be the mean or mid-point of the sample's age group. Development of chronic carriage was defined as a persistence of HBsAg seropositivity over at least 6 months, or progression from HBsAg or anti-HBc seropositivity to HBsAg seropositivity at least 6 months later.	Fitting age-specific risk of chronic infection function
**Disease progression in chronic HBV carriers and liver disease patients**	Extracted longitudinal data informing the natural history of chronic HBV infection or liver disease in West Africa included disease progression risks, rates and Kaplan-Meier survival curves, with long-term follow-up of a chronic HBV carrier cohort in The Gambia representing the most recent and highest-quality African evidence on this to date [8]. Progression rates from Mendy *et al.* 2008 [106] were excluded as these relate to the same participants as the subsequent follow-up analysis.<br>In studies where longitudinal outcomes were presented as the risk of the outcome over an average follow-up period or the mean time to the outcome, they were converted into rates per person-year using the following standard statistical equations, under the assumption that the rate of the event is constant over time [16]:<br>$Rate = -\frac{\ln(1 - risk\ up\ to\ time\ t)}{t}$<br>$Rate = \frac{1}{Average\ time\ to\ outcome}$	To capture the age-, sex- and time-dependent processes in the natural history of chronic HBV infection or isolate a subset of transitions in the model, calibration to some datapoints required simulation of specific cohorts of chronic carriers with known baseline age and infection status to replicate the characteristics of the original study population and thereby the calibration targets. Progression rates were calculated as the cumulative number of events of interest over the follow-up duration in the model, divided by the person-years spent in the compartments at risk. As in the corresponding cohort studies, compartments at risk were those containing individuals at risk of potentially developing the outcome of interest in the model (directly or indirectly). Person-years at risk were calculated as the sum of individuals in the respective compartments at each timepoint over the follow-up

Type of data	Details and assumptions	Use in model
	The cumulative probability of the outcomes of interest over time was extracted from Kaplan Meier survival curves and used directly as calibration targets. A survival curve for cirrhotic patients was additionally calculated from the deaths data in Diarra *et al.* 2010 using the life-table method [107].	period, multiplied by the timestep *dt*. Rates were stratified by age and sex where the data suggested relevant differences.<br>The cumulative probability of mortality (or other event) extracted from survival curves was calculated as the total incident deaths or events at a given timestep since entry into the cohort, divided by the population at risk at entry.
**Risk factors for liver disease in chronic HBV carriers**	All measures of association for clinical or demographic characteristics with development of liver disease in chronic carriers were extracted from the literature. Other identified odds ratios that could not be approximated as processes in the model were not included. Much evidence on risk factors for liver disease came from the Gambia Liver Cancer Study [42, 69, 108], of which only the paper with the most rigorous statistical analysis and the largest sample size was included [109].	Odds ratios were calculated in a given age group, sex and at a given timestep according to the standard epidemiological definition, by deriving the number of exposed and unexposed cases and controls.
**Cross-sectional characteristics of chronic HBV carriers and liver disease patients**	The majority of datapoints on characteristics of chronic HBV carriers or liver disease patients described the prevalence of different disease states. The extracted estimates highlight that it is often not possible to assign chronic carriers to a specific phase of chronic infection with a single cross-sectional measurement of different variables; for example, in the Gambian cohort at baseline almost 20% of study participants were unclassified [8]. These unclassified carriers were excluded in the calibration targets by removing them from the denominator. Additionally, the data on distribution of disease states does not correspond directly to the criteria for treatment eligibility, which required a more stringent ascertainment including a liver disease assessment.	Characteristics of chronic HBV carriers and liver disease patients were calculated as the cross-sectional proportion of individuals in a set of compartments, in a given age group, sex and timepoint.
**External model estimates of disease burden: HBV-related HCC incidence and mortality**	Age- and sex-specific estimates of country-specific liver cancer incidence and mortality in The Gambia were available from the GLOBOCAN database for the years 1988, 1998 and 2018. All liver cancer cases were assumed to represent HCC, as these are the most common [110]. HCC incidence estimated by GLOBOCAN at the International Agency for Research on Cancer is based on data from the National Cancer Registry of The Gambia [10], a population-based registry established in 1986 to collect data allowing to estimate the impact of HBV vaccination on liver cancer in the Gambia Hepatitis Intervention Study [111].<br>As estimates from GLOBOCAN represent population-wide HCC cases and deaths of any aetiology, these calibration targets were multiplied by	The modelled age- and sex-specific incidence of HCC and mortality from cirrhosis in different years was calibrated according to the definition of the annual incidence rate in GLOBOCAN, using the total population size at the given year mid-point as the denominator. The numerator for the population-based cirrhosis mortality and HCC incidence rates were the number of additional HBV-related deaths from the compensated and decompensated cirrhosis compartments and the number of incident HCC cases from all chronic carrier compartments in the given year,

Type of data	Details and assumptions	Use in model
	the population attributable fraction of HCC attributable to chronic HBV infection. In the scoping review, no longitudinal studies allowing to estimate the PAF were identified. Case-control studies were included if HCC or cirrhosis patients were recruited as cases, controls were individuals without chronic liver disease, and the exposure was a positive HBsAg test. Two Gambian studies, including the Gambia Liver Cancer Study, were identified providing data for this. Odds ratios from these studies were used to estimate the PAF using the standard epidemiological formula [112]: $$Population\ attributable\ fraction = P_{e(cases)} \frac{(OR-1)}{OR} \times 100$$ Where $P_{e(cases)}$ is the prevalence of HBsAg among HCC patients.<br><br>Based on this data, 57% of HCC was estimated to be attributable to HBsAg [42, 113]. The PAF for HBV-related HCC was assumed to be constant by age, sex and over time. Though some of the studies identified in the scoping review suggested a lower HBsAg prevalence and odds ratio in older HCC patients [42, 113], the sample size of the latter study was small and it was unclear how this would translate to the age-specific HBV-related HCC incidence rates. Previous meta-analyses on the PAF of liver cancer due to HBV also did not present differences by age [114, 115].	respectively. These rates were calculated across the specified age range
**External model estimates of disease burden: HBV-related cirrhosis mortality**	Age- and sex-specific estimates of cirrhosis mortality for the years 1990 and 2017 came from the Global Burden of Disease study. Global estimates of HBV-related cirrhosis mortality from GBD are based on data from vital registration and verbal autopsies, though the data sources for most sub-Saharan African countries were sparse [116]. No empirical data was available from The Gambia specifically and the data quality rating in the study was low, implying that these estimates are strongly determined by model covariates and regional patterns more generally. Cirrhosis mortality attributable to HBV was adopted from GBD directly, as their estimate of 49% in West Africa was almost identical to the Gambian data identified in the scoping review [69].	

Anti-HBc = hepatitis B core antibodies, anti-HBs = hepatitis B surface antibodies, GBD = Global Burden of Disease study, GLOBOCAN = Global Cancer Observatory, HBeAg = hepatitis B e antigen, HBsAg = hepatitis B surface antigen, HBV = hepatitis B virus, HCC = hepatocellular carcinoma, MTCT = mother-to-child transmission, OR = odds ratio, PAF = population attributable fraction.

Model fits

This section shows the model fits for most of the calibration targets described above. Model projections were consistent with the majority of empirical data used in the calibration. Notable features include the reduction in chronic infection prevalence in the age groups covered by the routine vaccination programme and other transmission patterns (**Figure S2.7**), early loss of HBeAg with age and the majority of adult chronic carriers having HBeAg-negative infection (**Figure S2.8**), the mortality experience of HCC patients (**Figure S2.9**), and age and sex patterns in hepatocellular carcinoma incidence (**Figure S2.10**), with a relatively young average age at onset of cirrhosis and HCC (**Table S2.8**). HBV-related cirrhosis mortality rates were less in agreement with the modelled estimates from the Global Burden of Disease Study (GBD) used as calibration target (**Figure S2.10**), with the model overestimating median cirrhosis mortality rates in women and at younger ages, and **Table S2.8** indicates discrepancies in cirrhosis outcomes between different data sources combined within the modelling framework.

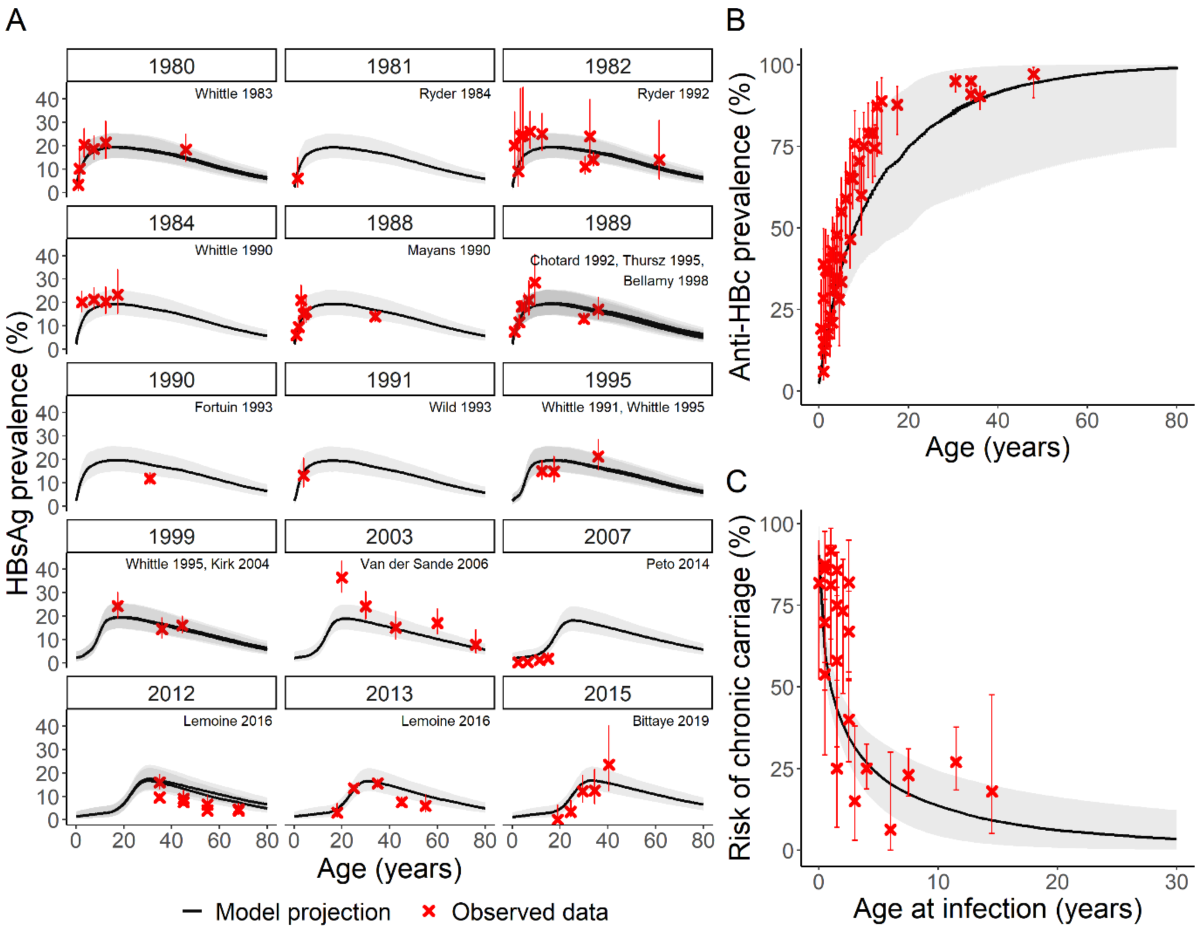

Figure S2.7. Calibrated and observed transmission patterns in The Gambia by age and over time. The median (black line) and 95% credible interval (grey) of model projections with the accepted parameter sets are shown. Red crosses and error bars represent the observed data

with 95% confidence intervals. Panel (A) shows HBsAg seroprevalence indicating current chronic infection at different timepoints, panel (B) shows pre-vaccination anti-HBc seroprevalence data (1980-1989) representing the proportion of the population that has ever been infected (including acute infections, chronic infections and recovery), and panel (C) shows the risk of chronic carriage determining which proportion of acute infections become chronic instead of recovering depending on the age at infection. Data in all panels were derived from a range of sources, but first author and year of publication are only shown in (A).

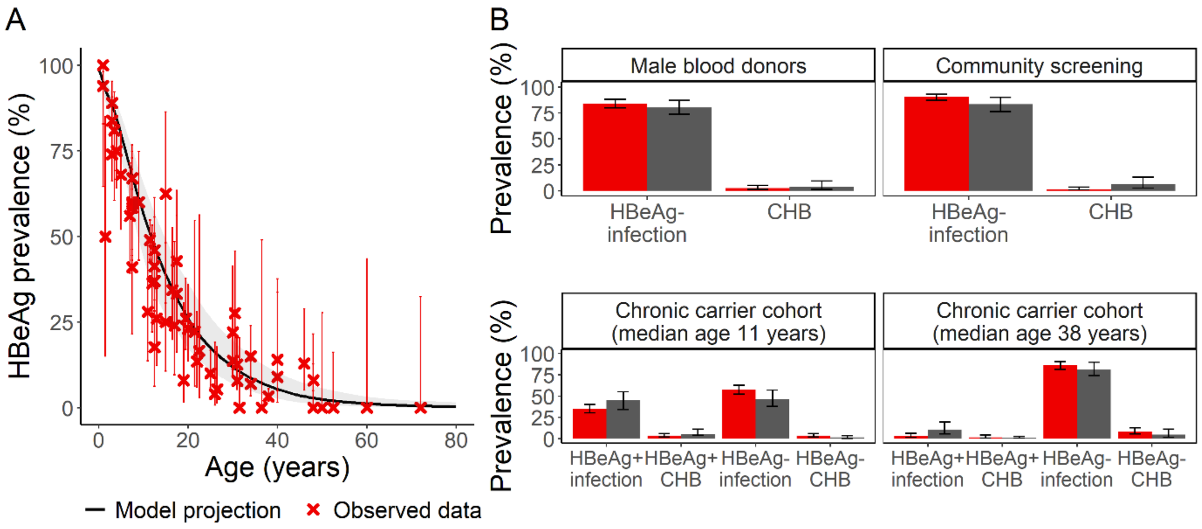

Figure S2.8. Model fit to the age-specific HBeAg prevalence in chronic carriers (A) and the distribution of disease states among chronic carriers (B). In (A), the observed age-specific HBeAg prevalence did not vary noticeably over time or by sex. In (B), modelled estimates are shown in grey and the data in red. Disease states are HBeAg-positive or -negative infection or chronic hepatitis B (CHB). The blood donor and community screening populations were represented in the model in terms of their reported sex and age distribution. HBeAg prevalence data was derived from various publications and data in (B) was derived from Lemoine *et al.* (2016) and Shimakawa *et al.* (2016) [8, 15].

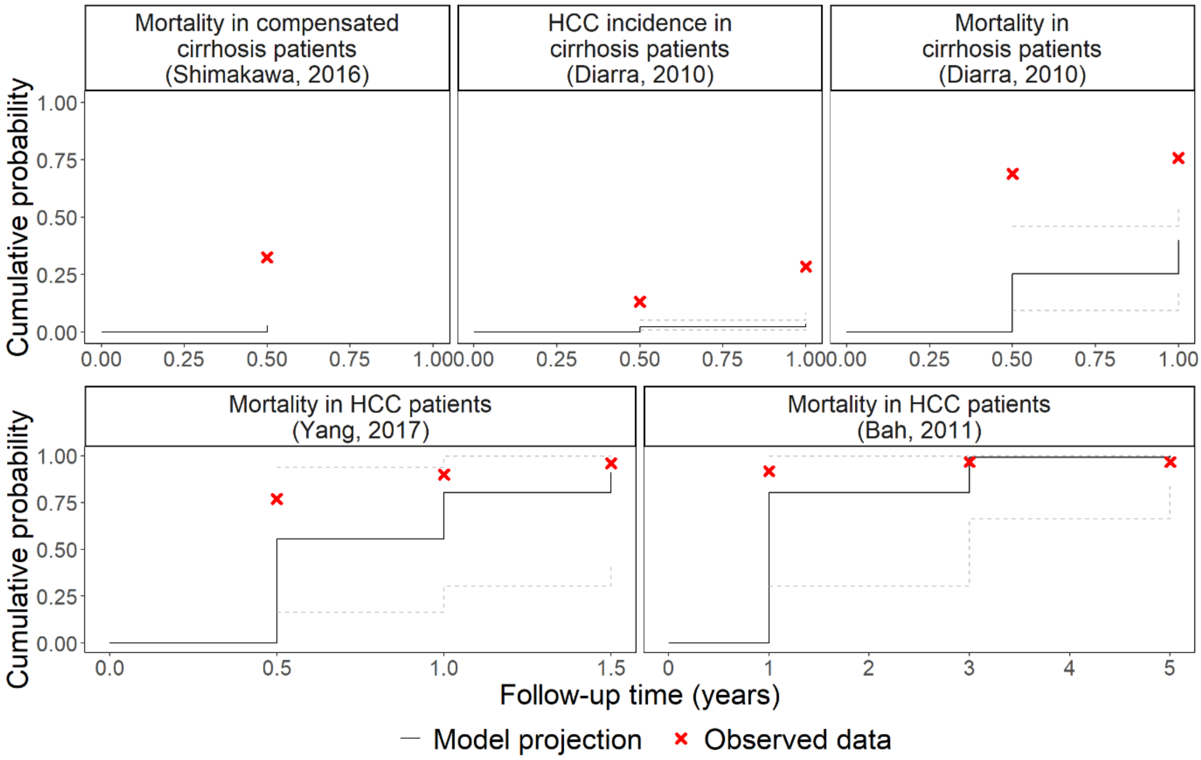

Figure S2.9. Model fit to the cumulative probability of death or HCC incidence over time in various liver disease cohorts. Solid lines show the median, and grey error bars the 95% credible interval of model projections with the accepted parameter sets.

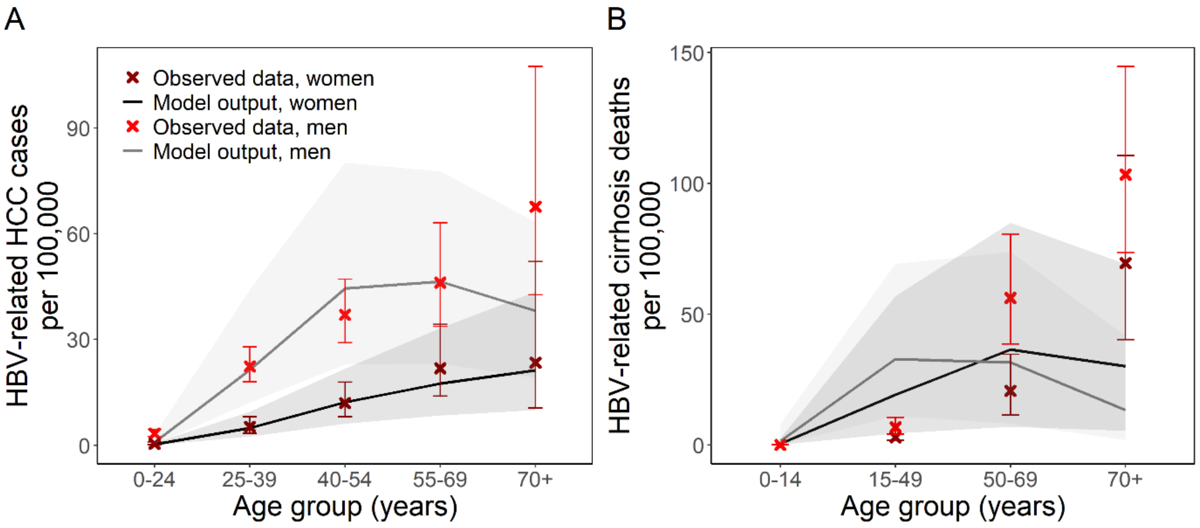

Figure S2.10. Model fit to the age- and sex-specific HBV-related hepatocellular carcinoma (HCC) incidence (A) and cirrhosis mortality rates (B) in The Gambia in 2018 and 2017, respectively. Country-specific liver cancer rates from GLOBOCAN [10], based on data from the national cancer registry, were multiplied by the population attributable fraction to obtain rates for HCC attributable to HBV. Modelled estimates of rates of HBV-related cirrhosis mortality came from the Global Burden of Disease Study [116]. The model was additionally

calibrated to data of HCC incidence in 1988 and 1998, and to cirrhosis mortality in 1990 from the same sources (not shown).

Table S2.8. Model fit to additional calibration targets. Crosses in the source column indicate that the data came from a West African country other than The Gambia. Asterisks show the datapoints that were upweighted in the calibration. Full references can be found in Table S1.

Outcome	First author & year of publication	Year	Study population	Age (years)	Observed estimate (95% confidence interval)	Calibrated estimate (95% credible interval)
**Transmission**						
Average force of infection in children in two Gambian villages (per 100 person-years)	Whittle, 1990	1980	General population	Range 0.5-4	17	9 (6-17)
Chronic infection incidence in susceptible children in The Gambia (per 100 person-years)	Ryder, 1984	1981	Hospital patients	Mean 1.9 (range 0.1-6)	7	3 (2-6)
Percentage of chronic infections attributable to MTCT (pre-vaccination) (%)	Shimakawa, 2016	1986	Chronic HBV carriers	All ages	16 (9-23)*	15 (6-31)
MTCT risk from HBsAg-positive mothers in West Africa, no vaccination (%)	Ayoola, 1981[x]	1979	HBsAg-positive mother-infant pairs	8 months	33 (12-64)	13 (5-28)
	Ayoola, 1987[x]	1985		3-12 months	15 (5-36)	
	Barin, 1981[x]	1979		Mean 6 months (range 3-15)	12 (4-29)	
	Kodjoh, 1992[x]	1989		2-11 months	16 (8-31)*	
**Disease progression in chronic HBV carriers**						
Incidence rate of HBeAg loss (per 100 person-years)	Shimakawa, 2016	1985	Chronic HBV carriers, male	Median 10.8 (IQR 4.6-21.8)	7.0 (5.7-8.6)*	9.1 (6.6-13.1)
			Chronic HBV carriers, female		8.2 (6.3-10.7)*	9.8 (7.2-13.7)
Incidence rate of HCC (per 100 person-years)			Chronic HBV carriers, male	Range 0-20	0*	0.11 (0.05-0.24)
			Chronic HBV carriers, male	Range 20-30	0.18 (0.08-0.41)*	0.24 (0.12-0.50)
			Chronic HBV carriers, female	Range 0-20	0*	0.02 (0.01-0.05)
			Chronic HBV carriers, female	Range 20-30	0*	0.05 (0.03-0.12)
Incidence rate of non-malignant end-stage liver disease (per 100 person-years)			Chronic HBV carriers	Median 10.8 (IQR 4.6-21.8)	0.02 (0.005-0.07)*	0.06 (0.02-0.13)
All-cause mortality rate (per 100 person-years)			Chronic HBV carriers, male		0.48 (0.32-0.71)*	1.02 (0.78-1.48)
			Chronic HBV carriers, female		0.32 (0.20-0.51)*	0.71 (0.60-1.02)
Rate of HBsAg loss in chronic HBV carrier children (per 100 person-years)	Coursaget, 1987[x]	1978	Chronic HBV carriers	Range 0-2 years	0	0.28 (0.21-0.35)
**Disease progression in liver disease patients**						
All-cause mortality rate in liver disease patients of mixed aetiology (per 100 person-years)	Olubuyide, 1996[x]	1983	Cirrhosis patients including with HCC	Mean 38 (range 12-82)	33.3	49.9 (27.1-62.5)
**Risk factors for liver disease in chronic HBV carriers**						

Outcome	First author & year of publication	Year	Study population	Age (years)	Observed estimate (95% confidence interval)	Calibrated estimate (95% credible interval)
Odds ratio for association of current HBeAg positivity and cirrhosis	Mendy, 2010	1999	HBsAg-positive hospital patients, male	15-84	6.2 (1.1-34.1)	2.2 (0.4-13.1)
Odds ratio for association of current HBeAg positivity and HCC					3.2 (0.7-15.3)	2.1 (0.9-4.6)
Odds ratio for association of male sex and significant liver fibrosis or cirrhosis	Shimakawa, 2016	2013	Chronic HBV carriers	8-96	5 (1.7-10.0)	1.7 (0.9-4.7)
**Cross-sectional characteristics of chronic HBV carriers**						
Prevalence of CC or DCC in chronic HBV carriers (%)	Lemoine, 2016	2012-2013	Blood donors – HBsAg-positive, male	27-35	7 (5-11)	6 (2-12)
Prevalence of HCC in chronic HBV carriers (%)					0 (0-1)	0.1 (0.03-0.7)
Prevalence of CC or DCC in chronic HBV carriers (%)			General population – HBsAg-positive	33-47	3 (2-5)*	4 (2-10)
Prevalence of HCC in chronic HBV carriers (%)					0 (0-1)*	0.1 (0.02-0.5)
Prevalence of HCC in chronic HBV carriers (%)	Shimakawa, 2016	1986	Chronic HBV carriers	4.5-22	0 (0-1)*	0.01 (0.00-0.05)
Prevalence of CC or DCC in chronic HBV carriers (%)		2013		8-95	0.4 (0.1-2.1)*	4 (1-9)
Age-specific prevalence of significant liver fibrosis or cirrhosis in chronic HBV carriers (%)		2013		8-29	7 (2-19)*	9 (6-15)
				30-39	6 (3-12)*	10 (6-17)
				40-49	6 (2-16)*	11 (6-20)
				50-95	4 (1-11)*	13 (6-24)
**Cross-sectional characteristics of liver disease patients**						
Percentage of deaths due to end-stage liver disease in a compensated cirrhosis cohort of mixed aetiology (%)	Shimakawa, 2016	2014	Compensated cirrhosis patients without HCC	27-53	63 (39-82)	27 (10-56)
Prevalence of HBeAg in HBsAg-positive HCC patients (%)	Mendy, 2010	1997-2001	HCC patients – HBsAg-positive	17-34	23 (13-38)	52 (30-71)
				35-44	11 (4-25)	24 (9-44)
				45-54	24 (13-41)	12 (3-28)
				55-64	10 (2-40)	6 (1-18)
				65-87	10 (2-40)	2 (0.38-11)
Prevalence of HBeAg in HBsAg-positive cirrhosis patients (%)			Cirrhosis patients – HBsAg-positive	17-34	32 (16-53)	30 (9-77)
				35-44	27 (10-56)	10 (2-43)
				45-54	20 (7-45)	4 (1-25)
				55-64	40 (12-77)	2 (0.35-15)
Prevalence of HBeAg in HBsAg-positive HCC patients (%)	Ryder, 1992	1982	HCC patients – HBsAg-positive	15-49	31 (17-49)	39 (20-60)
				50-72	6 (2-19)	6 (1-19)
Prevalence of CC in HCC patients (%)	Umoh, 2011	1999	HCC patients	15-67	32 (23-44)	37 (12-64)

Outcome	First author & year of publication	Year	Study population	Age (years)	Observed estimate (95% confidence interval)	Calibrated estimate (95% credible interval)
Prevalence of DCC in HCC patients (%)				15-67	30 (20-41)	12 (1-59)
Mean age of cirrhosis patients at presentation (years)	Mendy, 2010	1999	HBsAg-positive cirrhosis patients	Mean 39 (SD 12)	39 (36-42)	30 (24-35)
Mean age of HCC patients at presentation (years)			HBsAg-positive HCC patients	Mean 42 (SD 13)	42 (40-44)	40 (34-45)
Percentage of male sex among cirrhosis patients (%)			HBsAg-positive cirrhosis patients	Mean 39 (SD 12)	77 (64-86)	63 (47-82)
Percentage of male sex among HCC patients (%)			HBsAg-positive HCC patients	Mean 42 (SD 13)	84 (77-89)	79 (66-88)

CC = compensated cirrhosis, DCC = decompensated cirrhosis, HCC = hepatocellular carcinoma, IQR = interquartile range, MTCT = mother-to-child transmission, SD = standard deviation.

Prior and posterior model parameter distributions

Figure S2.11 shows the prior and posterior density distributions of model parameters varied in the calibration. For most parameters, the median and credible interval of posterior estimates were similar to the prior distributions, suggesting that prior evidence agreed with much of the epidemiological data used in the calibration and that calibration targets did not provide sufficient additional information to reduce the uncertainty range of these parameters. However, the calibration data allowed to update beliefs about several parameters, notably the progression rate from HBeAg-negative infection to HBeAg-negative CHB, the rate from HBeAg-positive infection to HBeAg-positive CHB, the age-dependent transmission coefficients (β_1, β_2, β_3), the coefficient for progression to HCC in women, and the progression rate from HBeAg-positive CHB to HBeAg-negative infection. The parameters remaining most uncertain after calibration, in terms of having the largest posterior interquartile range relative to the median, were the minimum age for HCC, the progression rate from HBeAg-positive to HBeAg-negative CHB, the progression rate from HBeAg-negative CHB to compensated cirrhosis, and the coefficient for progression through the HBeAg-positive compartments.

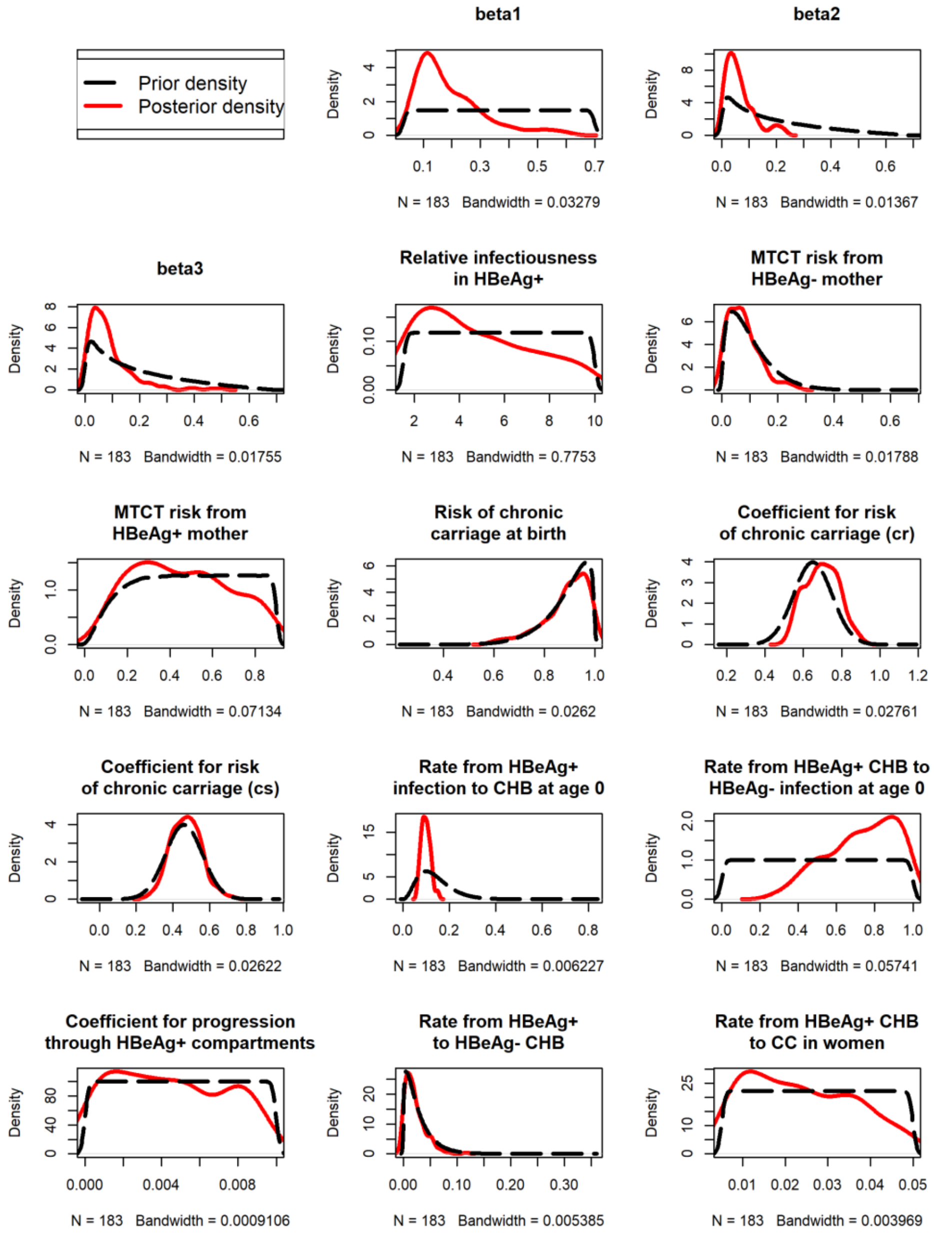
Prior density
Posterior density
beta1
N = 183 Bandwidth = 0.03279
beta2
N = 183 Bandwidth = 0.01367
beta3
N = 183 Bandwidth = 0.01755
Relative infectiousness in HBeAg+
N = 183 Bandwidth = 0.7753
MTCT risk from HBeAg- mother
N = 183 Bandwidth = 0.01788
MTCT risk from HBeAg+ mother
N = 183 Bandwidth = 0.07134
Risk of chronic carriage at birth
N = 183 Bandwidth = 0.0262
Coefficient for risk of chronic carriage (cr)
N = 183 Bandwidth = 0.02761
Coefficient for risk of chronic carriage (cs)
N = 183 Bandwidth = 0.02622
Rate from HBeAg+ infection to CHB at age 0
N = 183 Bandwidth = 0.006227
Rate from HBeAg+ CHB to HBeAg- infection at age 0
N = 183 Bandwidth = 0.05741
Coefficient for progression through HBeAg+ compartments
N = 183 Bandwidth = 0.0009106
Rate from HBeAg+ to HBeAg- CHB
N = 183 Bandwidth = 0.005385
Rate from HBeAg+ CHB to CC in women
N = 183 Bandwidth = 0.003969
Density

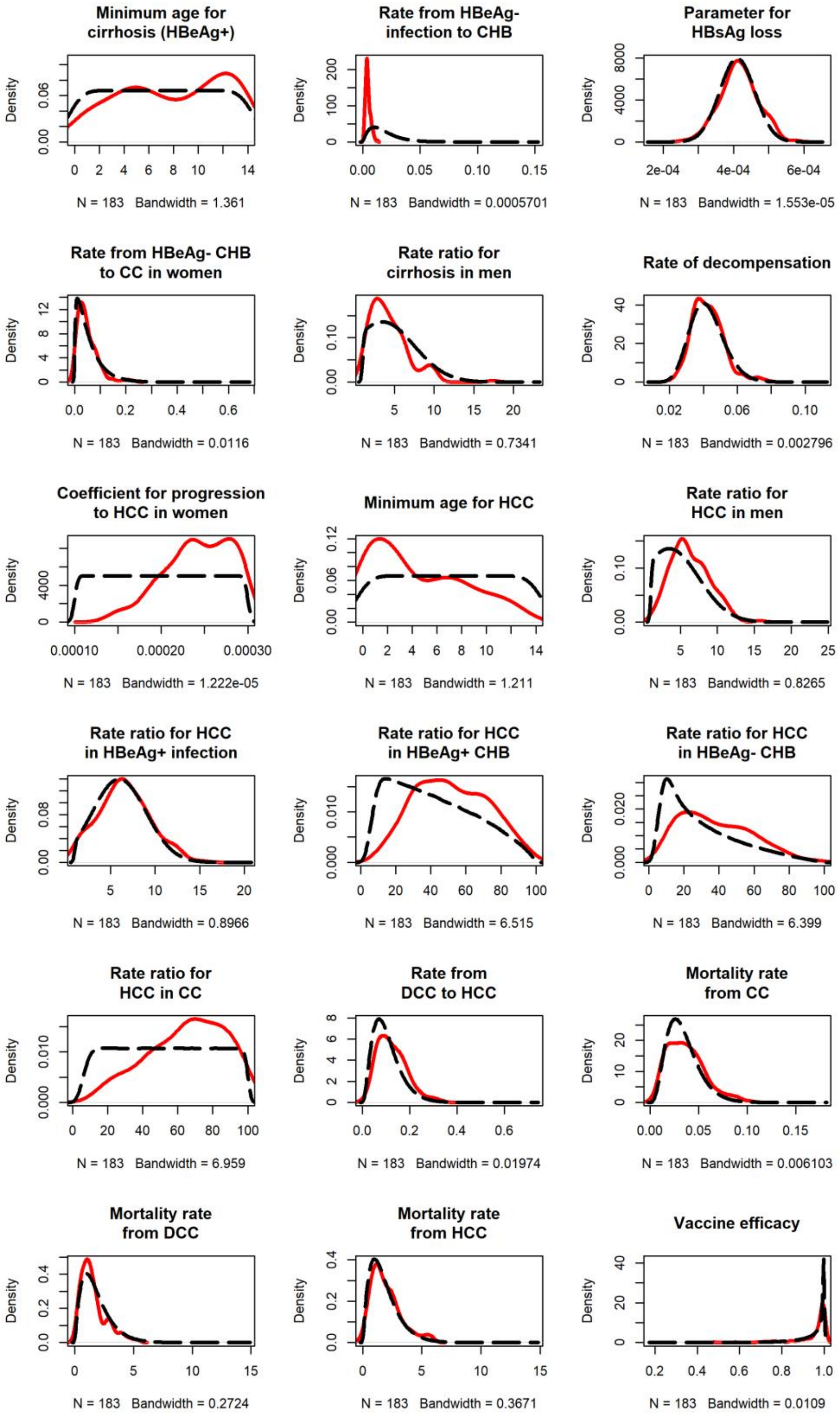

Figure S2.11. Prior and approximated posterior distributions for fitted model parameters. Details on parameters and their meaning can be found in **Table S2.4**. CC = compensated cirrhosis, CHB = chronic hepatitis B, DCC = decompensated cirrhosis, HBeAg- = negative for hepatitis B e antigen, HBeAg+ = positive for hepatitis B e antigen, HCC = hepatocellular carcinoma, MTCT = mother-to-child transmission.

D. Additional assumptions in cost-effectiveness analysis

Calculation of model outcomes

Disability-adjusted life years (DALYs) represent the years of life lost due to premature HBV-related death or disability associated with person-time spent with decompensated cirrhosis and HCC. Total years of life lost due to HBV were calculated by multiplying the number of HBV-related deaths at a given age and in a given year by the corresponding estimate of remaining life expectancy from the UN World Population Prospects [58]. To calculate the years lived with disability, disability weights of 0.178 and 0.54 for decompensated cirrhosis and HCC, respectively, were derived from the Global Burden of Disease Study 2017 and assumed to be the same irrespective of treatment status [117]. Model outcomes were calculated over the lifetime in the diagnosed cohort as well as in the overall Gambian population. The long-term time horizon allowed to account for the remaining life expectancy of all treated carriers as well as potential downstream effects on the prevention of new HBV infections.

Costing

All costs were estimated from a healthcare provider perspective in 2020 US dollars (US$). Cost estimates from previous years and in different currencies were converted into US$ and adjusted for inflation using the Gambia gross domestic product (GDP) deflator rate [118]. Unit costs of these different components were applied to the resources utilised over time in the model, allowing to derive the total costs incurred in different scenarios. The cost of tenofovir and the rapid diagnostic test was derived from a previous global study [119]. Per-person programme costs of the screening intervention were assumed to be the same as in a community-based screening programme in The Gambia [36]. Laboratory cost data on the different diagnostic tests involved in clinical management of chronic HBV infection were collected locally, including the associated cost of human resources, upfront purchase and maintenance of diagnostic devices. Viral load testing is available at relatively low cost in The Gambia using an in-house PCR, but close to the GeneXpert costs estimated in a previous global study [119].

Our analysis was conducted from a health provider perspective, which does not account for labour productivity losses associated with premature deaths from HBV-related liver disease or the costs of end-of-life care provided by family members [36]. Additionally, potential non-linearities between costs of diagnosis and treatment and their coverage levels were not taken into account for this study because estimating these economies of scale in the absence of implementation data is challenging, though reductions in unit costs would be likely if the

intervention is scaled up to the high levels modelled in this study [120]. These assumptions could have led to an underestimation of the cost-effectiveness of the modelled strategies.

We also assumed that provision of the treatment programme would not save costs associated with management of cirrhosis or HCC because of the limited access to medical care for end-stage liver disease in the public sector in most of sub-Saharan Africa [30]. A previous cost-effectiveness analysis in The Gambia assumed that screening and treatment would avert some costs due to this, but varying hospitalisation costs for decompensated cirrhosis and HCC in sensitivity analyses had a negligible effect on the incremental cost-effectiveness ratio (ICER) of the screening and treatment programme in that study [36]. In our scoping review, we identified only few studies with data on the hospital costs of clinical management for cirrhosis and HCC in African countries, and the frequency of hospital admissions due to this in The Gambia could not reliably be determined. Comparison of the number of hospitalised patients identified in the country-wide Gambia Liver Cancer Study and a more recent study with estimates of HBV-related liver cancer incidence nevertheless gives an indication that this has been low historically [42, 121]. It is therefore possible that provision of a screening and treatment programme could lead to increased identification, hospital admission and medical care for end-stage liver disease as well, especially since treatment does not fully prevent the development of HCC. Based on this limited data, we assumed that either costs incurred or saved through this would have relatively little effect on cost-effectiveness ratios in this study within uncertainty bounds, but this requires further data and consideration in future studies.

3. Supplementary results

A. Base case projections of the impact of infant vaccination

Figure S3.1 shows the annual incidence of chronic HBV infections and HBV-related deaths under the base case scenario of infant vaccination and a hypothetical scenario in which no HBV interventions were introduced historically.

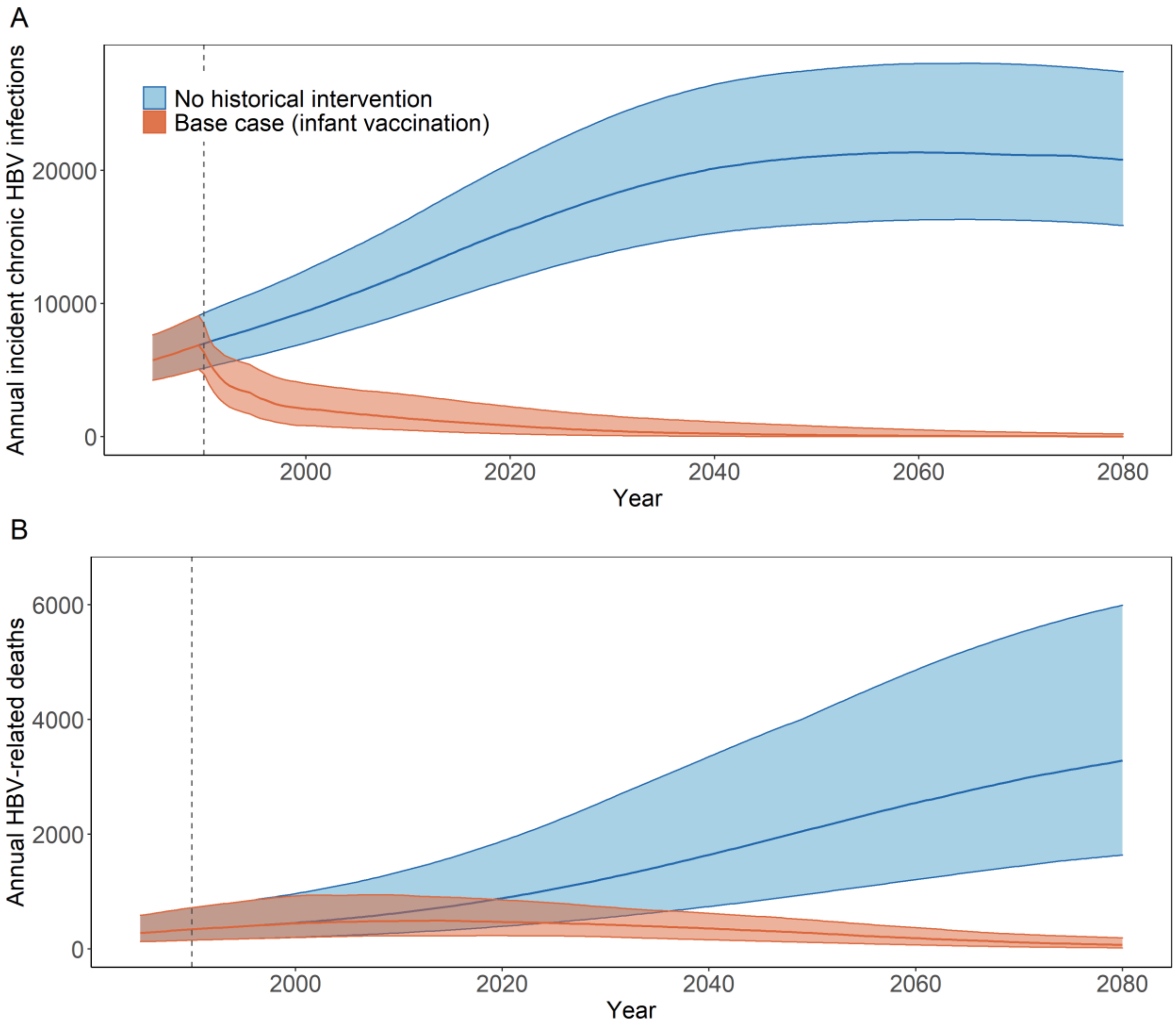

Figure S3.1. Impact of infant vaccination on the incidence of new chronic HBV infections (A) and HBV-related deaths over time. The dashed vertical line represents the year of introduction of the vaccine (1990) in the base case scenario.

B. Cost-effectiveness of different monitoring strategies

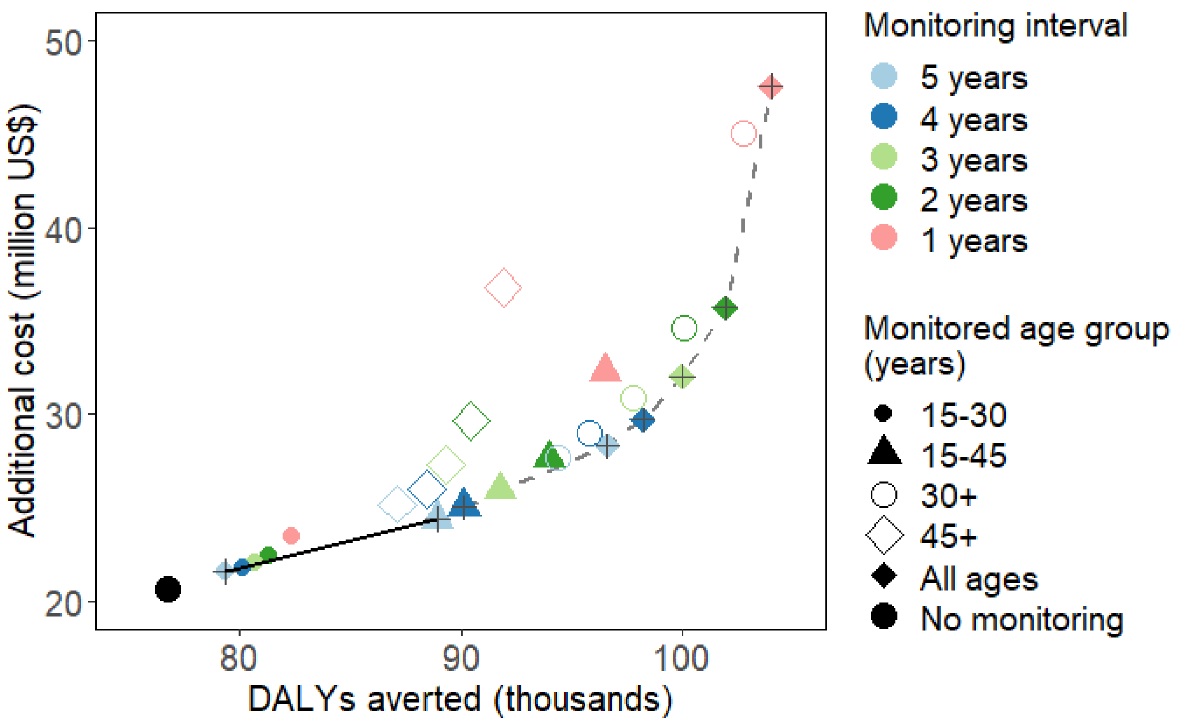

Figure S3.2. Cost-effectiveness plane for different treatment and monitoring strategies. Median costs and disability-adjusted life years (DALYs) averted between 2020 and 2100 are shown. Lines connect non-dominated strategies, with the solid line indicating a slope representing an incremental cost-effectiveness ratio (ICER) below the cost-effectiveness threshold of US$404 per DALY averted and dashed lines indicating ICERs over the threshold. Axes start at the cost and impact of the screening and treatment programme without monitoring.

C. Sensitivity analysis of cost-effectiveness results

Table S3.1. Sensitivity of model outcomes to all model parameters varied in the calibration. Partial rank correlation coefficients (PRCC) and p-values are shown for the impact and cost-effectiveness ratio of screening and treatment without monitoring compared to no treatment, and for 5-yearly monitoring in under 45-year-olds compared to screening and treatment without monitoring. Parameters with the 10 highest PRCC for each outcome are coloured, and PRCC ≥ ± 0.5 are highlighted in blue.

**Model parameter**	**Screening and treatment programme without monitoring**				**Screening and treatment programme with 5-yearly in <45 year olds**			
	Incremental DALYs averted		Incremental cost-effectiveness ratio		Incremental DALYs averted		Incremental cost-effectiveness ratio	
	PRCC	*p*	PRCC	*p*	PRCC	*p*	PRCC	*p*
**Rate from HBeAg- infection to HBeAg- CHB**	0.73	<0.001	-0.75	<0.001	0.74	<0.001	-0.91	<0.001
**Rate from HBeAg- CHB to CC in women**	0.33	<0.001	-0.68	<0.001	0.68	<0.001	-0.88	<0.001
**Rate from HBeAg+ CHB to CC in women**	0.53	<0.001	-0.60	<0.001	0.32	<0.001	-0.55	<0.001
**Rate from HBeAg+ to HBeAg- CHB**	0.44	<0.001	-0.51	<0.001	-0.08	0.26	-0.31	<0.001
**MTCT risk from HBeAg+ mother**	0.43	<0.001	-0.38	<0.001	0.62	<0.001	-0.30	<0.001
**Rate from HBeAg+ CHB to HBeAg- infection at age 0**	-0.41	<0.001	0.36	<0.001	-0.12	0.11	0.20	0.006
**Coefficient for risk of chronic carriage (cr)**	-0.38	<0.001	0.30	<0.001	-0.31	<0.001	0.16	0.03
**Rate ratio for cirrhosis in men**	0.15	0.04	-0.29	<0.001	0.26	<0.001	-0.60	<0.001
**beta1**	0.27	<0.001	-0.27	<0.001	0.21	0.005	-0.16	0.03
**Risk of chronic carriage at birth**	0.25	<0.001	-0.26	<0.001	0.26	<0.001	-0.10	0.18
**Relative infectiousness in HBeAg+**	0.28	<0.001	-0.26	<0.001	0.24	<0.001	-0.13	0.07
**beta2**	0.26	<0.001	-0.23	0.002	0.22	0.003	-0.10	0.20
**Minimum age for HCC**	0.29	<0.001	-0.23	0.002	0.18	0.01	0.01	0.93
**MTCT risk from HBeAg- mother**	0.27	<0.001	-0.21	0.004	0.50	<0.001	-0.09	0.20
**Minimum age for cirrhosis (HBeAg+)**	-0.18	0.02	0.21	0.005	0.00	0.96	0.02	0.75
**Rate ratio for HCC in men**	-0.29	<0.001	0.20	0.006	-0.22	0.003	-0.09	0.24
**Mortality rate from CC**	0.06	0.38	-0.20	0.007	0.41	<0.001	-0.45	<0.001
**Rate from HBeAg+ infection to HBeAg+ CHB at age 0**	-0.26	<0.001	-0.19	0.008	-0.47	<0.001	-0.24	<0.001
**Coefficient for risk of chronic carriage (cs)**	-0.37	<0.001	0.19	0.01	-0.33	<0.001	0.04	0.61

Model parameter	Screening and treatment programme without monitoring				Screening and treatment programme with 5-yearly in <45 year olds			
**Mortality rate from DCC**	0.17	0.02	-0.18	0.01	0.23	0.002	-0.18	0.02
**Vaccine efficacy**	-0.25	<0.001	0.17	0.02	-0.38	<0.001	0.12	0.11
**Parameter for HBsAg loss**	0.04	0.59	-0.14	0.06	0.08	0.31	-0.23	0.001
**Mortality rate from HCC**	0.07	0.32	-0.13	0.08	0.14	0.06	-0.19	0.009
**Rate from DCC to HCC**	0.13	0.07	-0.12	0.12	0.17	0.03	-0.11	0.12
**Rate ratio for HCC in CC**	-0.18	0.01	0.08	0.29	-0.06	0.44	-0.23	0.001
**Rate ratio for HCC in HBeAg+ infection**	0.05	0.49	-0.08	0.30	0.03	0.65	-0.10	0.18
**Rate of decompensation**	-0.03	0.67	-0.07	0.35	0.12	0.10	-0.26	<0.001
**Rate ratio for HCC in HBeAg- CHB**	-0.07	0.32	-0.07	0.36	-0.14	0.07	-0.12	0.10
**Rate ratio for HCC in HBeAg+ CHB**	0.03	0.66	0.05	0.46	0.07	0.37	0.14	0.05
**Coefficient for progression through HBeAg+ compartments**	-0.19	0.01	0.03	0.64	-0.22	0.002	0.01	0.89
**Coefficient for progression to HCC in women**	-0.13	0.07	-0.02	0.76	-0.14	0.06	-0.22	0.003
**beta3**	0.11	0.15	0.00	0.97	0.13	0.08	0.13	0.08

CC = compensated cirrhosis, CHB = chronic hepatitis B, DCC = decompensated cirrhosis, HBeAg- = negative for hepatitis B e antigen, HBeAg+ = positive for hepatitis B e antigen, HCC = hepatocellular carcinoma, MTCT = mother-to-child transmission.

Among all parameters varied in the calibration, uncertainty in the incremental impact and cost-effectiveness ratio (CER) of the treatment programme without monitoring, as well as of the 5-yearly monitoring in <45-year-olds compared to no monitoring, was most associated with uncertainty in the progression rate from HBeAg-negative infection to HBeAg-negative CHB, and parameters relating to progression to, decompensation or mortality from cirrhosis (Table S2.1). At the lower estimates of these parameters inferred in the calibration, the treatment programme with 5-yearly monitoring in <45-year-olds would not be considered cost-effective under the cost-effectiveness threshold of US$404 per DALY averted, whereas the treatment programme without monitoring had a CER below the estimated cost-effectiveness threshold under almost all calibrated parameter estimates.

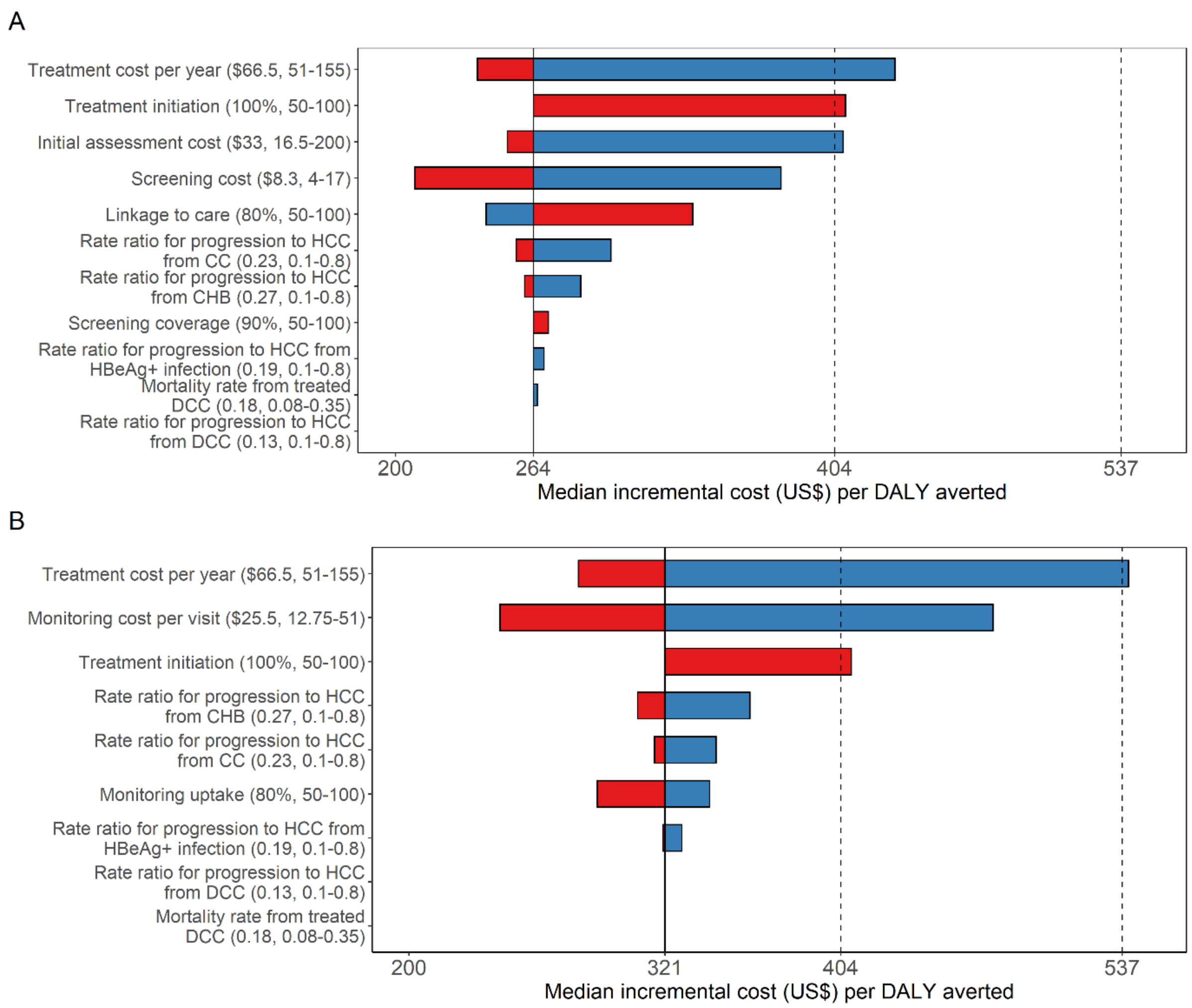

Figure S3.3. One-way sensitivity analysis of fixed model parameters. The effect of variations in the fixed parameters of costs and coverage at different stages of care, and of treatment effect on disease progression, are shown for (A) the median cost-effectiveness ratio of the screening and treatment programme without monitoring compared to the base case of no treatment and (B) of the screening and treatment programme with 5-yearly monitoring in <45 year olds compared to no monitoring. Costs are in units of US$ (2020 prices) per person, and the mortality rate from treated DCC is per person-year. The fixed estimate and the range over which parameters were varied is shown in brackets. Blue and red bars represent the change in ICER for an increase or decrease in the parameter value, respectively. The solid vertical line represents the median ICER in the primary analysis, and dashed vertical lines show estimates of the cost-effectiveness threshold in The Gambia of 0.52 and 0.69 times the GDP per capita. Monitoring parameters do not affect the treatment programme without monitoring in (A), and screening and initial assessment parameters do not affect the impact or cost of adding monitoring to the treatment programme (B), and are therefore omitted. CC = compensated cirrhosis, CHB = chronic hepatitis B, DCC = decompensated cirrhosis, HCC = hepatocellular carcinoma.

In one-way sensitivity analysis, projections of CERs for the same scenarios were robust to variations in parameters of treatment effect, and conclusions about the most effective cost-effective strategy were not affected by varying reductions in HCC progression on treatment between 20-90% (**Figure S3.3**). However, assumptions about uptake at different stages of

care had larger effects on CERs. Reductions in linkage to care and treatment initiation led to larger reductions in health impact than in costs due to the costs incurred at previous stages of care, and thereby increased the CER of the treatment programme without monitoring. Among coverage parameters, the largest increase in CER, up to US$409 (95% CrI 243-784) per DALY averted, occurred for a reduction of the proportion initiating treatment to 50%. With a reduced probability of linkage to care, the no monitoring strategy would be more strongly dominated by 5-yearly monitoring in <45-year-olds, while assumptions of lower screening coverage would result in both lower health impact and reduced total costs, thereby having only limited effect on ICERs (not shown).

Cost estimates at all stages of care had among the greatest effect on the cost-effectiveness of the screening and treatment programme overall, with CERs being most sensitive to an increased treatment cost within the assumed ranges, which includes the cost of tenofovir and annual monitoring (**Figure S3.3**). Conversely, the CER of the screening and treatment programme without monitoring and with 5-yearly monitoring in under 45-year-olds were most reduced by reductions in the cost of screening and monitoring, respectively.

Further comparison of the incremental cost-effectiveness ratios for all monitoring strategies under wide ranges of plausible cost estimates showed that at least one of the treatment strategies had a median ICER below current estimates of the cost-effectiveness threshold under all cost assumptions (**Figure S3.4**). Monitoring at any frequency would not be cost-effective if both the initial and monitoring assessments were very costly, but monitoring became more cost-effective compared to no monitoring for higher costs involved with initial identification of carriers (screening and assessment) or lower long-term costs involved with treatment and monitoring. 5-yearly monitoring in 15-45 year olds remained the most effective cost-effective strategy for most individual variations in the cost at different stages of care, but extending the 5-yearly monitoring to all ages became cost-effective if the cost of monitoring was halved. Nevertheless, more frequent monitoring remained unlikely to be cost-effective under current thresholds even for substantial reductions in both the cost of initial clinical assessment and monitoring (**Figure S3.4B**).

Discounting costs and impact at 0% or 5% or adopting a shorter time horizon did also not change the conclusion about 5-yearly monitoring in under 45 year olds being the most effective cost-effective strategy (not shown).

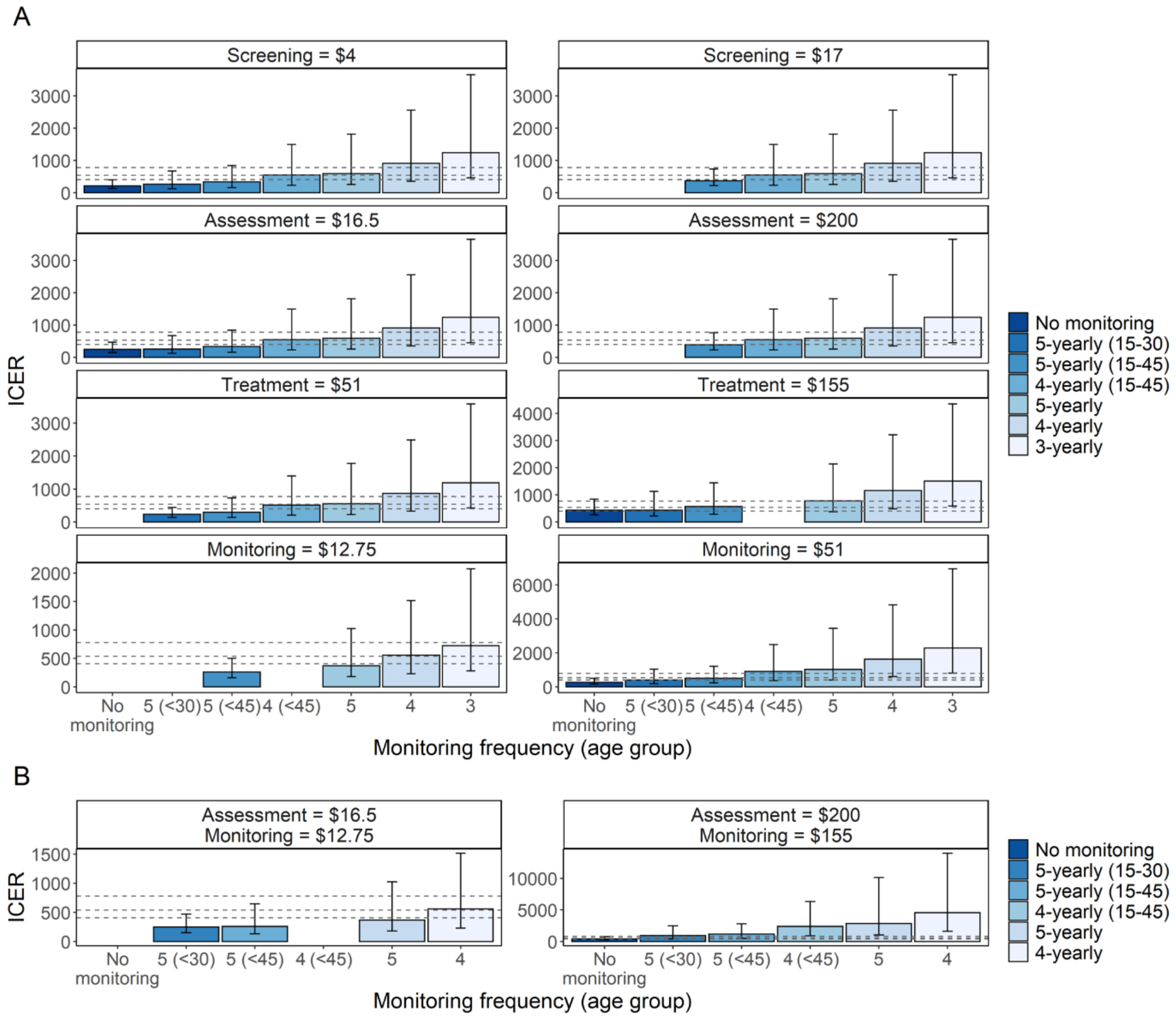

Figure S3.4. Sensitivity analysis of variation of incremental cost-effectiveness ratios (ICERs) over plausible cost ranges for all monitoring strategies. In (A), costs at each stage of care were varied individually, and (B) shows the effect of joint variation in the cost of initial assessment and monitoring. ICERs are in units of US$ (2020 prices) per DALY averted and cost estimates are per person (one-time for screening and assessment, annual for treatment and dependent on frequency for monitoring). Monitoring frequencies are in years and applied across all ages unless the age group is shown in brackets. Dashed lines indicate the estimated cost-effectiveness thresholds of 0.52 and 0.69 times the GDP per capita in The Gambia, as well as 1 times the GDP per capita. ICERs are calculated for two consecutive non-dominated strategies; 1- and 2-yearly (A), and 3-yearly (B) monitoring frequencies were not dominated but omitted from the figure for readability, and blank spaces occur if strategies are dominated for a given cost.

D. Sensitivity analysis of the impact the treatment programme on reducing HBV incidence

Due to the small percentage of chronic carriers being initiated on treatment, the model projected that the one-time screening and treatment programme would only have minimal effect on the incidence of new chronic HBV infections in the population (**Figure S2.5**). This was despite adults and in particular pregnant women representing the main source of transmission in the post-vaccination period in the model (not shown). The same was true in a sensitivity analysis assuming no onwards transmission from treated carriers, in which the screening and treatment programme only averted 7% (5-13%) of new chronic infections occurring by 2100, and 96% (88-100%) of DALYs averted by the treatment programme were averted in the treated cohort.

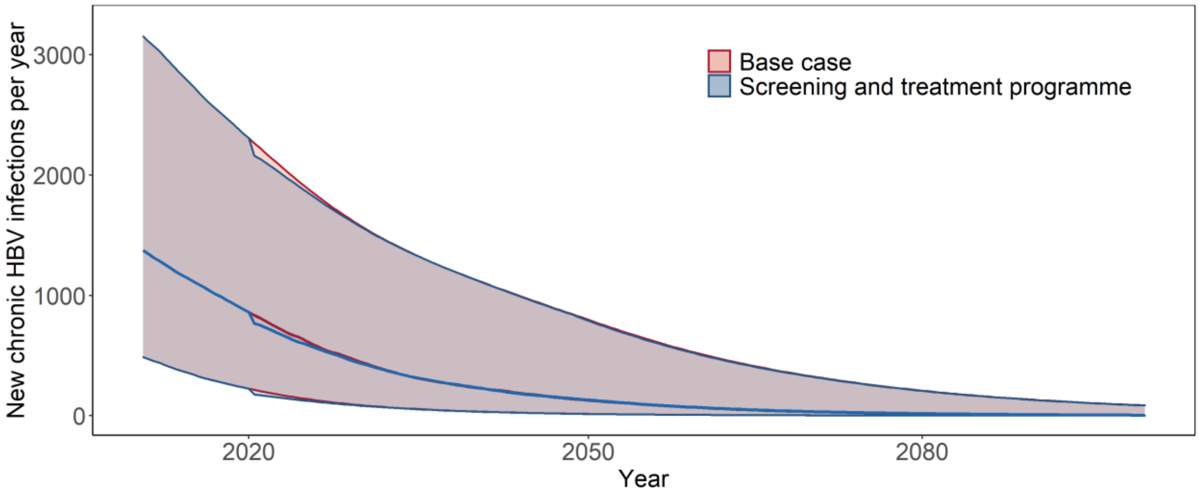

Figure S3.5. Impact of the screening and treatment programme without monitoring on the annual incidence of chronic HBV infections. The median line and 95% uncertainty bounds almost completely overlap between the two scenarios.

4. References

1. Edmunds WJ, Medley GF, Nokes DJ, Hall AJ, Whittle HC. The influence of age on the development of the hepatitis B carrier state. Proc Biol Sci **1993**; 253(1337): 197-201.
2. Keane E, Funk AL, Shimakawa Y. Systematic review with meta-analysis: the risk of mother-to-child transmission of hepatitis B virus infection in sub-Saharan Africa. Aliment Pharmacol Ther **2016**; 44(10): 1005-17.
3. Nayagam S, Thursz M, Sicuri E, Conteh L, Wiktor S, Low-Beer D, Hallett TB. Requirements for global elimination of hepatitis B: a modelling study. Lancet Infect Dis **2016**; 16(12): 1399-408.
4. Lin X, Robinson NJ, Thursz M, Rosenberg DM, Weild A, Pimenta JM, Hall AJ. Chronic hepatitis B virus infection in the Asia-Pacific region and Africa: review of disease progression. J Gastroenterol Hepatol **2005**; 20(6): 833-43.
5. Schmit N, Nayagam S, Thursz MR, Hallett TB. The global burden of chronic hepatitis B virus infection: comparison of country-level prevalence estimates from four research groups. Int J Epidemiol **2020**; 50(2): 560-9.
6. Matthews PC, Geretti AM, Goulder PJ, Klenerman P. Epidemiology and impact of HIV coinfection with hepatitis B and hepatitis C viruses in Sub-Saharan Africa. J Clin Virol **2014**; 61(1): 20-33.
7. Stockdale AJ, Chaponda M, Beloukas A, Phillips RO, Matthews PC, Papadimitropoulos A, King S, Bonnett L, Geretti AM. Prevalence of hepatitis D virus infection in sub-Saharan Africa: a systematic review and meta-analysis. Lancet Glob Health **2017**; 5(10): e992-e1003.
8. Shimakawa Y, Lemoine M, Njai HF, Bottomley C, Ndow G, Goldin RD, Jatta A, Jeng-Barry A, Wegmuller R, Moore SE, Baldeh I, Taal M, D'Alessandro U, Whittle H, Njie R, Thursz M, Mendy M. Natural history of chronic HBV infection in West Africa: a longitudinal population-based study from The Gambia. Gut **2016**; 65(12): 2007-16.
9. de Martel C, Maucort-Boulch D, Plummer M, Franceschi S. World-wide relative contribution of hepatitis B and C viruses in hepatocellular carcinoma. Hepatology **2015**; 62(4): 1190-200.
10. Ferlay J, Ervik M, Lam F, Colombet M, Mery L, Piñeros M, Znaor A, Soerjomataram I, Bray F. Global Cancer Observatory: Cancer Today. Available at: https://gco.iarc.fr/. Accessed 06/05/19.
11. Mokdad AA, Lopez AD, Shahraz S, Lozano R, Mokdad AH, Stanaway J, Murray CJ, Naghavi M. Liver cirrhosis mortality in 187 countries between 1980 and 2010: a systematic analysis. BMC Med **2014**; 12: 145.
12. Institute for Health Metrics and Evaluation. Global Burden of Disease Study 2017 Results Tool. Available at: http://ghdx.healthdata.org/gbd-results-tool. Accessed 04/10/2019.
13. Ree GH. Hepatitis associated antigen, cirrhosis and primary carcinoma of the liver in the Gambia. Trans R Soc Trop Med Hyg **1975**; 69(2): 263-5.
14. Ghosh S, Sow A, Guillot C, Jeng A, Ndow G, Njie R, Toure S, Diop M, Mboup S, Kane CT, Lemoine M, Thursz M, Zoulim F, Mendy M, Chemin I. Implementation of an in-house quantitative real-time polymerase chain reaction method for Hepatitis B virus quantification in West African countries. J Viral Hepat **2016**; 23(11): 897-904.
15. Lemoine M, Shimakawa Y, Njie R, Taal M, Ndow G, Chemin I, Ghosh S, Njai HF, Jeng A, Sow A, Toure-Kane C, Mboup S, Suso P, Tamba S, Jatta A, Sarr L, Kambi A, Stanger W, Nayagam S, Howell J, Mpabanzi L, Nyan O, Corrah T, Whittle H, Taylor-Robinson SD, D'Alessandro U, Mendy M, Thursz MR. Acceptability and feasibility of a screen-and-treat programme for hepatitis B virus infection in The Gambia: the Prevention of Liver Fibrosis and Cancer in Africa (PROLIFICA) study. Lancet Glob Health **2016**; 4(8): e559-67.

16. Kirkwood BR, Sterne JAC. Essential medical statistics. Malden, Mass.: Blackwell Science, **2003**.
17. R Core Team. R: A language and environment for statistical computing. Available at: https://www.R-project.org/.
18. Edmunds WJ, Medley GF, Nokes DJ, O'Callaghan CJ, Whittle HC, Hall AJ. Epidemiological patterns of hepatitis B virus (HBV) in highly endemic areas. Epidemiol Infect **1996**; 117(2): 313-25.
19. Stanaway JD, Flaxman AD, Naghavi M, Fitzmaurice C, Vos T, Abubakar I, Abu-Raddad LJ, Assadi R, Bhala N, Cowie B, Forouzanfour MH, Groeger J, Hanafiah KM, Jacobsen KH, James SL, MacLachlan J, Malekzadeh R, Martin NK, Mokdad AA, Mokdad AH, Murray CJL, Plass D, Rana S, Rein DB, Richardus JH, Sanabria J, Saylan M, Shahraz S, So S, Vlassov VV, Weiderpass E, Wiersma ST, Younis M, Yu C, El Sayed Zaki M, Cooke GS. The global burden of viral hepatitis from 1990 to 2013: findings from the Global Burden of Disease Study 2013. Lancet **2016**; 388(10049): 1081-8.
20. Edmunds WJ, Medley GF, Nokes DJ. The transmission dynamics and control of hepatitis B virus in The Gambia. Stat Med **1996**; 15(20): 2215-33.
21. Okpechi IG, Ameh OI, Bello AK, Ronco P, Swanepoel CR, Kengne AP. Epidemiology of Histologically Proven Glomerulonephritis in Africa: A Systematic Review and Meta-Analysis. PLoS One **2016**; 11(3): e0152203.
22. European Association for the Study of the Liver. Clinical Practice Guidelines on the management of hepatitis B virus infection. J Hepatol **2017**; 67(2): 370-98.
23. Breakwell L, Tevi-Benissan C, Childs L, Mihigo R, Tohme R. The status of hepatitis B control in the African region. Pan Afr Med J **2017**; 27(Suppl 3): 17.
24. World Health Organization. WHO/UNICEF Estimates of National Immunization Coverage. Available at: https://apps.who.int/immunization_monitoring/globalsummary/wucoveragecountrylist.html. Accessed 07/10/2019.
25. Marion SA, Tomm Pastore M, Pi DW, Mathias RG. Long-term follow-up of hepatitis B vaccine in infants of carrier mothers. Am J Epidemiol **1994**; 140(8): 734-46.
26. Pan CQ, Duan ZP, Bhamidimarri KR, Zou HB, Liang XF, Li J, Tong MJ. An algorithm for risk assessment and intervention of mother to child transmission of hepatitis B virus. Clin Gastroenterol Hepatol **2012**; 10(5): 452-9.
27. World Health Organization. Official country reported coverage estimates time series. Available at: https://apps.who.int/immunization_monitoring/globalsummary/timeseries/tscoveragehepb%5Fbd.html. Accessed 17/05/2019.
28. Miyahara R, Jasseh M, Gomez P, Shimakawa Y, Greenwood B, Keita K, Ceesay S, D'Alessandro U, Roca A. Barriers to timely administration of birth dose vaccines in The Gambia, West Africa. Vaccine **2016**; 34(29): 3335-41.
29. Moturi E, Tevi-Benissan C, Hagan JE, Shendale S, Mayenga D, Murokora D, Patel M, Hennessey K, Mihigo R. Implementing a Birth Dose of Hepatitis B Vaccine in Africa: Findings from Assessments in 5 Countries. J Immunol Sci **2018**; Suppl(5): 31-40.
30. Lemoine M, Thursz MR. Battlefield against hepatitis B infection and HCC in Africa. J Hepatol **2017**; 66(3): 645-54.
31. Ndow G, Gore ML, Shimakawa Y, Suso P, Jatta A, Tamba S, Sow A, Toure-Kane C, Sadiq F, Sabally S, Njie R, Thursz MR, Lemoine M. Hepatitis B testing and treatment in HIV patients in The Gambia-Compliance with international guidelines and clinical outcomes. PLoS One **2017**; 12(6): e0179025.
32. Polaris Observatory Collaborators. Global prevalence, treatment, and prevention of hepatitis B virus infection in 2016: a modelling study. Lancet Gastroenterol Hepatol **2018**; 3(6): 383-403.
33. Shimakawa Y, Bottomley C, Njie R, Mendy M. The association between maternal hepatitis B e antigen status, as a proxy for perinatal transmission, and the risk of hepatitis B e antigenaemia in Gambian children. BMC Public Health **2014**; 14: 532.

34. Gerlich WH. Reduction of infectivity in chronic hepatitis B virus carriers among healthcare providers and pregnant women by antiviral therapy. Intervirology **2014**; 57(3-4): 202-11.
35. Salkic NN, Zildzic M, Muminhodzic K, Pavlovic-Calic N, Zerem E, Ahmetagic S, Mott-Divkovic S, Alibegovic E. Intrafamilial transmission of hepatitis B in Tuzla region of Bosnia and Herzegovina. Eur J Gastroenterol Hepatol **2007**; 19(2): 113-8.
36. Nayagam S, Conteh L, Sicuri E, Shimakawa Y, Suso P, Tamba S, Njie R, Njai H, Lemoine M, Hallett TB, Thursz M. Cost-effectiveness of community-based screening and treatment for chronic hepatitis B in The Gambia: an economic modelling analysis. Lancet Glob Health **2016**; 4(8): e568-78.
37. Lin SM, Yu ML, Lee CM, Chien RN, Sheen IS, Chu CM, Liaw YF. Interferon therapy in HBeAg positive chronic hepatitis reduces progression to cirrhosis and hepatocellular carcinoma. J Hepatol **2007**; 46(1): 45-52.
38. Hsu YS, Chien RN, Yeh CT, Sheen IS, Chiou HY, Chu CM, Liaw YF. Long-term outcome after spontaneous HBeAg seroconversion in patients with chronic hepatitis B. Hepatology **2002**; 35(6): 1522-7.
39. Chen CJ, Yang HI. Natural history of chronic hepatitis B REVEALed. J Gastroenterol Hepatol **2011**; 26(4): 628-38.
40. Sun J, Robinson L, Lee NL, Welles S, Evans AA. No contribution of lifestyle and environmental exposures to gender discrepancy of liver disease severity in chronic hepatitis b infection: Observations from the Haimen City cohort. PLoS One **2017**; 12(4): e0175482.
41. Raffetti E, Fattovich G, Donato F. Incidence of hepatocellular carcinoma in untreated subjects with chronic hepatitis B: a systematic review and meta-analysis. Liver Int **2016**; 36(9): 1239-51.
42. Kirk GD, Lesi OA, Mendy M, Akano AO, Sam O, Goedert JJ, Hainaut P, Hall AJ, Whittle H, Montesano R. The Gambia Liver Cancer Study: Infection with hepatitis B and C and the risk of hepatocellular carcinoma in West Africa. Hepatology **2004**; 39(1): 211-9.
43. Chen CJ, Yang HI, Su J, Jen CL, You SL, Lu SN, Huang GT, Iloeje UH. Risk of hepatocellular carcinoma across a biological gradient of serum hepatitis B virus DNA level. JAMA **2006**; 295(1): 65-73.
44. Boglione L, Cusato J, Cariti G, Di Perri G, D'Avolio A. The E genotype of hepatitis B: clinical and virological characteristics, and response to interferon. J Infect **2014**; 69(1): 81-7.
45. Chu CM, Liaw YF. Incidence and risk factors of progression to cirrhosis in inactive carriers of hepatitis B virus. Am J Gastroenterol **2009**; 104(7): 1693-9.
46. D'Amico G, Garcia-Tsao G, Pagliaro L. Natural history and prognostic indicators of survival in cirrhosis: a systematic review of 118 studies. J Hepatol **2006**; 44(1): 217-31.
47. Hui AY, Chan HL, Leung NW, Hung LC, Chan FK, Sung JJ. Survival and prognostic indicators in patients with hepatitis B virus-related cirrhosis after onset of hepatic decompensation. J Clin Gastroenterol **2002**; 34(5): 569-72.
48. Thiele M, Gluud LL, Fialla AD, Dahl EK, Krag A. Large variations in risk of hepatocellular carcinoma and mortality in treatment naive hepatitis B patients: systematic review with meta-analyses. PLoS One **2014**; 9(9): e107177.
49. Mittal S, Kramer JR, Omino R, Chayanupatkul M, Richardson PA, El-Serag HB, Kanwal F. Role of Age and Race in the Risk of Hepatocellular Carcinoma in Veterans With Hepatitis B Virus Infection. Clin Gastroenterol Hepatol **2017**.
50. Attia KA, Ackoundou-N'guessan K C, N'Dri-Yoman AT, Mahassadi AK, Messou E, Bathaix YF, Kissi YH. Child-Pugh-Turcott versus Meld score for predicting survival in a retrospective cohort of black African cirrhotic patients. World J Gastroenterol **2008**; 14(2): 286-91.
51. Olubuyide IO. The natural history of primary liver cell carcinoma: a study of 89 untreated adult Nigerians. Cent Afr J Med **1992**; 38(1): 25-30.

52. Peto TJ, Mendy ME, Lowe Y, Webb EL, Whittle HC, Hall AJ. Efficacy and effectiveness of infant vaccination against chronic hepatitis B in the Gambia Hepatitis Intervention Study (1986-90) and in the nationwide immunisation program. BMC Infect Dis **2014**; 14: 7.
53. Fortuin M, Chotard J, Jack AD, Maine NP, Mendy M, Hall AJ, Inskip HM, George MO, Whittle HC. Efficacy of hepatitis B vaccine in the Gambian expanded programme on immunisation. The Lancet **1993**; 341(8853): 1129-31.
54. Viviani S, Jack A, Hall AJ, Maine N, Mendy M, Montesano R, Whittle HC. Hepatitis B vaccination in infancy in The Gambia: protection against carriage at 9 years of age. Vaccine **1999**; 17(23-24): 2946-50.
55. van der Sande MA, Waight PA, Mendy M, Zaman S, Kaye S, Sam O, Kahn A, Jeffries D, Akum AA, Hall AJ, Bah E, McConkey SJ, Hainaut P, Whittle HC. Long-term protection against HBV chronic carriage of Gambian adolescents vaccinated in infancy and immune response in HBV booster trial in adolescence. PLoS One **2007**; 2(8): e753.
56. Mendy M, Peterson I, Hossin S, Peto T, Jobarteh ML, Jeng-Barry A, Sidibeh M, Jatta A, Moore SE, Hall AJ, Whittle H. Observational study of vaccine efficacy 24 years after the start of hepatitis B vaccination in two Gambian villages: no need for a booster dose. PLoS One **2013**; 8(3): e58029.
57. World Health Organization. Hepatitis B vaccines: WHO position paper – July 2017. Wkly Epidemiol Rec **2017**; 92(27): 369–92.
58. United Nations Department of Economic and Social Affairs Population Division. World Population Prospects 2017. Available at: https://esa.un.org/unpd/wpp/. Accessed 25/04/2018.
59. Chang Y, Choe WH, Sinn DH, Lee JH, Ahn SH, Lee H, Shim JJ, Jun DW, Park SY, Nam JY, Cho EJ, Yu SJ, Lee DH, Lee JM, Kim YJ, Kwon SY, Paik SW, Yoon JH. Nucleos(t)ide Analogue Treatment for Patients With Hepatitis B Virus (HBV) e Antigen-Positive Chronic HBV Genotype C Infection: A Nationwide, Multicenter, Retrospective Study. J Infect Dis **2017**; 216(11): 1407-14.
60. Nguyen MH, Yang HI, Le A, Henry L, Nguyen N, Lee MH, Zhang J, Wong C, Wong C, Trinh H. Reduced Incidence of Hepatocellular Carcinoma in Cirrhotic and Noncirrhotic Patients With Chronic Hepatitis B Treated With Tenofovir-A Propensity Score-Matched Study. J Infect Dis **2019**; 219(1): 10-8.
61. Desalegn H, Aberra H, Berhe N, Medhin G, Mekasha B, Gundersen SG, Johannessen A. Predictors of mortality in patients under treatment for chronic hepatitis B in Ethiopia: a prospective cohort study. BMC Gastroenterol **2019**; 19(1): 74.
62. Beguelin C, Fall F, Seydi M, Wandeler G. The current situation and challenges of screening for and treating hepatitis B in sub-Saharan Africa. Expert Rev Gastroenterol Hepatol **2018**; 12(6): 537-46.
63. Aberra H, Desalegn H, Berhe N, Medhin G, Stene-Johansen K, Gundersen SG, Johannessen A. Early experiences from one of the first treatment programs for chronic hepatitis B in sub-Saharan Africa. BMC Infect Dis **2017**; 17(1): 438.
64. Shimakawa Y, Njie R, Ndow G, Vray M, Mbaye PS, Bonnard P, Sombie R, Nana J, Leroy V, Bottero J, Ingiliz P, Post G, Sanneh B, Baldeh I, Suso P, Ceesay A, Jeng A, Njai HF, Nayagam S, D'Alessandro U, Chemin I, Mendy M, Thursz M, Lemoine M. Development of a simple score based on HBeAg and ALT for selecting patients for HBV treatment in Africa. J Hepatol **2018**; 69(4): 776-84.
65. World Health Organization. Guidelines for the Prevention, Care and Treatment of Persons with Chronic Hepatitis B Infection. Geneva: World Health Organization, **2015**.
66. Medley GF, Lindop NA, Edmunds WJ, Nokes DJ. Hepatitis-B virus endemicity: heterogeneity, catastrophic dynamics and control. Nat Med **2001**; 7(5): 619-24.
67. Fattovich G, Bortolotti F, Donato F. Natural history of chronic hepatitis B: special emphasis on disease progression and prognostic factors. J Hepatol **2008**; 48(2): 335-52.

68. Hui CK, Leung N, Yuen ST, Zhang HY, Leung KW, Lu L, Cheung SK, Wong WM, Lau GK. Natural history and disease progression in Chinese chronic hepatitis B patients in immune-tolerant phase. Hepatology **2007**; 46(2): 395-401.
69. Kuniholm MH, Lesi OA, Mendy M, Akano AO, Sam O, Hall AJ, Whittle H, Bah E, Goedert JJ, Hainaut P, Kirk GD. Aflatoxin exposure and viral hepatitis in the etiology of liver cirrhosis in the Gambia, West Africa. Environ Health Perspect **2008**; 116(11): 1553-7.
70. Yang JD, Hainaut P, Gores GJ, Amadou A, Plymoth A, Roberts LR. A global view of hepatocellular carcinoma: trends, risk, prevention and management. Nat Rev Gastroenterol Hepatol **2019**; 16(10): 589-604.
71. Peak CM, Reilly AL, Azman AS, Buckee CO. Prolonging herd immunity to cholera via vaccination: Accounting for human mobility and waning vaccine effects. PLoS Negl Trop Dis **2018**; 12(2): e0006257.
72. Grossi G, Viganò M, Loglio A, Lampertico P. Hepatitis B virus long-term impact of antiviral therapy nucleot(s)ide analogues (NUCs). Liver Int **2017**; 37 Suppl 1: 45-51.
73. Tseng CH, Hsu YC, Chen TH, Ji F, Chen IS, Tsai YN, Hai H, Thuy LTT, Hosaka T, Sezaki H, Borghi JA, Cheung R, Enomoto M, Nguyen MH. Hepatocellular carcinoma incidence with tenofovir versus entecavir in chronic hepatitis B: a systematic review and meta-analysis. Lancet Gastroenterol Hepatol **2020**; 5(12): 1039-52.
74. Hill A, Gotham D, Cooke G, Bhagani S, Andrieux-Meyer I, Cohn J, Fortunak J. Analysis of minimum target prices for production of entecavir to treat hepatitis B in high- and low-income countries. J Virus Erad **2015**; 1(2): 103-10.
75. Desalegn H, Aberra H, Berhe N, Mekasha B, Stene-Johansen K, Krarup H, Pereira AP, Gundersen SG, Johannessen A. Treatment of chronic hepatitis B in sub-Saharan Africa: 1-year results of a pilot program in Ethiopia. BMC Med **2018**; 16(1): 234.
76. Aberra H, Desalegn H, Berhe N, Mekasha B, Medhin G, Gundersen SG, Johannessen A. The WHO guidelines for chronic hepatitis B fail to detect half of the patients in need of treatment in Ethiopia. J Hepatol **2019**; 70(6): 1065-71.
77. Marcellin P, Heathcote EJ, Buti M, Gane E, de Man RA, Krastev Z, Germanidis G, Lee SS, Flisiak R, Kaita K, Manns M, Kotzev I, Tchernev K, Buggisch P, Weilert F, Kurdas OO, Shiffman ML, Trinh H, Washington MK, Sorbel J, Anderson J, Snow-Lampart A, Mondou E, Quinn J, Rousseau F. Tenofovir disoproxil fumarate versus adefovir dipivoxil for chronic hepatitis B. N Engl J Med **2008**; 359(23): 2442-55.
78. Cuenca-Gomez JA, Lozano-Serrano AB, Cabezas-Fernandez MT, Soriano-Perez MJ, Vazquez-Villegas J, Estevez-Escobar M, Cabeza-Barrera I, Salas-Coronas J. Chronic hepatitis B genotype E in African migrants: response to nucleos(t)ide treatment in real clinical practice. BMC Infect Dis **2018**; 18(1): 568.
79. Marcellin P, Wong DK, Sievert W, Buggisch P, Petersen J, Flisiak R, Manns M, Kaita K, Krastev Z, Lee SS, Cathcart AL, Crans G, Op den Brouw M, Jump B, Gaggar A, Flaherty J, Buti M. Ten-year efficacy and safety of tenofovir disoproxil fumarate treatment for chronic hepatitis B virus infection. Liver Int **2019**.
80. Marcellin P, Gane E, Buti M, Afdhal N, Sievert W, Jacobson IM, Washington MK, Germanidis G, Flaherty JF, Aguilar Schall R, Bornstein JD, Kitrinos KM, Subramanian GM, McHutchison JG, Heathcote EJ. Regression of cirrhosis during treatment with tenofovir disoproxil fumarate for chronic hepatitis B: a 5-year open-label follow-up study. Lancet **2013**; 381(9865): 468-75.
81. Liu K, Choi J, Le A, Yip TC, Wong VW, Chan SL, Chan HL, Nguyen MH, Lim YS, Wong GL. Tenofovir disoproxil fumarate reduces hepatocellular carcinoma, decompensation and death in chronic hepatitis B patients with cirrhosis. Aliment Pharmacol Ther **2019**; 50(9): 1037-48.
82. Papatheodoridis GV, Chan HL, Hansen BE, Janssen HL, Lampertico P. Risk of hepatocellular carcinoma in chronic hepatitis B: assessment and modification with current antiviral therapy. J Hepatol **2015**; 62(4): 956-67.
83. Papatheodoridis GV, Dalekos GN, Yurdaydin C, Buti M, Goulis J, Arends P, Sypsa V, Manolakopoulos S, Mangia G, Gatselis N, Keskin O, Savvidou S, Hansen BE,

Papaioannou C, Galanis K, Idilman R, Colombo M, Esteban R, Janssen HL, Lampertico P. Incidence and predictors of hepatocellular carcinoma in Caucasian chronic hepatitis B patients receiving entecavir or tenofovir. J Hepatol **2015**; 62(2): 363-70.

84. Papatheodoridis GV, Idilman R, Dalekos GN, Buti M, Chi H, van Boemmel F, Calleja JL, Sypsa V, Goulis J, Manolakopoulos S, Loglio A, Siakavellas S, Keskin O, Gatselis N, Hansen BE, Lehretz M, de la Revilla J, Savvidou S, Kourikou A, Vlachogiannakos I, Galanis K, Yurdaydin C, Berg T, Colombo M, Esteban R, Janssen HLA, Lampertico P. The risk of hepatocellular carcinoma decreases after the first 5 years of entecavir or tenofovir in Caucasians with chronic hepatitis B. Hepatology **2017**; 66(5): 1444-53.
85. Pan CQ, Chan S, Trinh H, Yao A, Bae H, Lou L. Similar efficacy and safety of tenofovir in Asians and non-Asians with chronic hepatitis B. World J Gastroenterol **2015**; 21(18): 5524-31.
86. Wu CY, Lin JT, Ho HJ, Su CW, Lee TY, Wang SY, Wu C, Wu JC. Association of nucleos(t)ide analogue therapy with reduced risk of hepatocellular carcinoma in patients with chronic hepatitis B: a nationwide cohort study. Gastroenterology **2014**; 147(1): 143-51.e5.
87. Shimakawa Y, Boucheron P, Luong Nguyen LB, Lemoine M, Sombie R. Performance of two simplified HBV treatment criteria (TREAT-B score and WHO guidelines) in Burkina Faso, West Africa. J Hepatol **2019**.
88. Wong GL. Management of chronic hepatitis B patients in immunetolerant phase: what latest guidelines recommend. Clin Mol Hepatol **2018**; 24(2): 108-13.
89. Lee HW, Chon YE, Kim BK, Yip TC, Tse YK, Wong GL, Wong VW, Chan HL, Ahn SH. Negligible HCC risk during stringently defined untreated immune-tolerant phase of chronic hepatitis B. Eur J Intern Med **2021**; 84: 68-73.
90. Yeo YH, Ho HJ, Yang HI, Tseng TC, Hosaka T, Trinh HN, Kwak MS, Park YM, Fung JYY, Buti M, Rodríguez M, Treeprasertsuk S, Preda CM, Ungtrakul T, Charatcharoenwitthaya P, Li X, Li J, Zhang J, Le MH, Wei B, Zou B, Le A, Jeong D, Chien N, Kam L, Lee CC, Riveiro-Barciela M, Istratescu D, Sriprayoon T, Chong Y, Tanwandee T, Kobayashi M, Suzuki F, Yuen MF, Lee HS, Kao JH, Lok AS, Wu CY, Nguyen MH. Factors Associated With Rates of HBsAg Seroclearance in Adults With Chronic HBV Infection: A Systematic Review and Meta-analysis. Gastroenterology **2019**; 156(3): 635-46.e9.
91. Hadziyannis E, Hadziyannis S. Current practice and contrasting views on discontinuation of nucleos(t)ide analog therapy in chronic hepatitis B. Expert Rev Gastroenterol Hepatol **2020**; 14(4): 243-51.
92. Berg T, Simon KG, Mauss S, Schott E, Heyne R, Klass DM, Eisenbach C, Welzel TM, Zachoval R, Felten G, Schulze-Zur-Wiesch J, Cornberg M, Op den Brouw ML, Jump B, Reiser H, Gallo L, Warger T, Petersen J. Long-term response after stopping tenofovir disoproxil fumarate in non-cirrhotic HBeAg-negative patients - FINITE study. J Hepatol **2017**; 67(5): 918-24.
93. Papatheodoridis GV, Rigopoulou EI, Papatheodoridi M, Zachou K, Xourafas V, Gatselis N, Hadziyannis E, Vlachogiannakos J, Manolakopoulos S, Dalekos GN. DARING-B: discontinuation of effective entecavir or tenofovir disoproxil fumarate long-term therapy before HBsAg loss in non-cirrhotic HBeAg-negative chronic hepatitis B. Antivir Ther **2018**; 23(8): 677-85.
94. Buti M, Wong DK, Gane E, Flisiak R, Manns M, Kaita K, Janssen HLA, Op den Brouw M, Jump B, Kitrinos K, Crans G, Flaherty J, Gaggar A, Marcellin P. Safety and efficacy of stopping tenofovir disoproxil fumarate in patients with chronic hepatitis B following at least 8 years of therapy: a prespecified follow-up analysis of two randomised trials. Lancet Gastroenterol Hepatol **2019**; 4(4): 296-304.
95. Su CW, Wu CY, Lin JT, Ho HJ, Wu JC. Nucleos(t)ide analogue continuous therapy associated with reduced adverse outcomes of chronic hepatitis B. J Chin Med Assoc **2020**; 83(2): 125-33.

96. Song J, Yang F, Wang S, Tikande S, Deng Y, Tang W, Cao G. Efficacy and safety of antiviral treatment on blocking the mother-to-child transmission of hepatitis B virus: A meta-analysis. J Viral Hepat **2019**; 26(3): 397-406.
97. Terrault NA, Lok ASF, McMahon BJ, Chang KM, Hwang JP, Jonas MM, Brown RS, Jr., Bzowej NH, Wong JB. Update on prevention, diagnosis, and treatment of chronic hepatitis B: AASLD 2018 hepatitis B guidance. Hepatology **2018**; 67(4): 1560-99.
98. Ford N, Scourse R, Lemoine M, Hutin Y, Bulterys M, Shubber Z, Donchuk D, Wandeler G. Adherence to Nucleos(t)ide Analogue Therapies for Chronic Hepatitis B Infection: A Systematic Review and Meta-Analysis. Hepatol Commun **2018**; 2(10): 1160-7.
99. Lintusaari J, Gutmann MU, Dutta R, Kaski S, Corander J. Fundamentals and Recent Developments in Approximate Bayesian Computation. Syst Biol **2017**; 66(1): e66-e82.
100. Toni T, Welch D, Strelkowa N, Ipsen A, Stumpf MP. Approximate Bayesian computation scheme for parameter inference and model selection in dynamical systems. J R Soc Interface **2009**; 6(31): 187-202.
101. van der Vaart E, Prangle D, Sibly RM. Taking error into account when fitting models using Approximate Bayesian Computation. Ecol Appl **2018**; 28(2): 267-74.
102. McKinley TJ, Vernon I, Andrianakis I, McCreesh N, Oakley JE, Nsubuga RN, Goldstein M, White RG. Approximate Bayesian Computation and simulation-based inference for complex stochastic epidemic models. Statistical science **2018**; 33(1): 4-18.
103. McKinley T, Cook AR, Deardon R. Inference in epidemic models without likelihoods. The International Journal of Biostatistics **2009**; 5(1).
104. Minter A, Retkute R. Approximate Bayesian Computation for infectious disease modelling. Epidemics **2019**; 29: 100368.
105. World Health Organization. Global and Country Estimates of immunization coverage and chronic HBV infection. Available at: http://whohbsagdashboard.com. Accessed 15/07/21.
106. Mendy ME, McConkey SJ, Sande van der MA, Crozier S, Kaye S, Jeffries D, Hall AJ, Whittle HC. Changes in viral load and HBsAg and HBeAg status with age in HBV chronic carriers in The Gambia. Virol J **2008**; 5: 49.
107. Diarra M, Konate A, Soukho A, Dicko M, Kalle A, Doumbia K, Sow H, Traore HA, Maiga MY. [Changing aspects of cirrhotic disease in a hepato-gastroenterology service in Mali]. Mali med **2010**; 25(1): 42-6.
108. Gouas DA, Villar S, Ortiz-Cuaran S, Legros P, Ferro G, Kirk GD, Lesi OA, Mendy M, Bah E, Friesen MD, Groopman J, Chemin I, Hainaut P. TP53 R249S mutation, genetic variations in HBX and risk of hepatocellular carcinoma in The Gambia. Carcinogenesis **2012**; 33(6): 1219-24.
109. Mendy ME, Welzel T, Lesi OA, Hainaut P, Hall AJ, Kuniholm MH, McConkey S, Goedert JJ, Kaye S, Rowland-Jones S, Whittle H, Kirk GD. Hepatitis B viral load and risk for liver cirrhosis and hepatocellular carcinoma in The Gambia, West Africa. J Viral Hepat **2010**; 17(2): 115-22.
110. Maucort-Boulch D, de Martel C, Franceschi S, Plummer M. Fraction and incidence of liver cancer attributable to hepatitis B and C viruses worldwide. Int J Cancer **2018**; 31: 31.
111. Bah E, Carrieri MP, Hainaut P, Bah Y, Nyan O, Taal M. 20-years of population-based cancer registration in hepatitis B and liver cancer prevention in the Gambia, West Africa. PLoS One **2013**; 8(9): e75775.
112. Webb P, Bain C, Page A. Essential Epidemiology: An Introduction for Students and Health Professionals (3rd ed.). Cambridge: Cambridge University Press, **2016**.
113. Ryder RW, Whittle HC, Sanneh AB, Ajdukiewicz AB, Tulloch S, Yvonnet B. Persistent hepatitis B virus infection and hepatoma in The Gambia, west Africa. A case-control study of 140 adults and their 603 family contacts. Am J Epidemiol **1992**; 136(9): 1122-31.
114. Raza SA, Clifford GM, Franceschi S. Worldwide variation in the relative importance of hepatitis B and hepatitis C viruses in hepatocellular carcinoma: a systematic review. Br J Cancer **2007**; 96(7): 1127-34.

115. de Martel C, Georges D, Bray F, Ferlay J, Clifford GM. Global burden of cancer attributable to infections in 2018: a worldwide incidence analysis. Lancet Glob Health **2020**; 8(2): e180-e90.
116. GBD 2017 Cirrhosis Collaborators. The global, regional, and national burden of cirrhosis by cause in 195 countries and territories, 1990-2017: a systematic analysis for the Global Burden of Disease Study 2017. Lancet Gastroenterol Hepatol **2020**; 5(3): 245-66.
117. Global Burden of Disease Collaborative Network. Global Burden of Disease Study 2017 (GBD 2017) Disability Weights. Available at: http://ghdx.healthdata.org/record/ihme-data/gbd-2017-disability-weights. Accessed 04/10/19.
118. Turner HC, Lauer JA, Tran BX, Teerawattananon Y, Jit M. Adjusting for Inflation and Currency Changes Within Health Economic Studies. Value Health **2019**; 22(9): 1026-32.
119. Tordrup D, Hutin Y, Stenberg K, Lauer JA, Hutton DW, Toy M, Scott N, Chhatwal J, Ball A. Cost-Effectiveness of Testing and Treatment for Hepatitis B Virus and Hepatitis C Virus Infections: An Analysis by Scenarios, Regions, and Income. Value Health **2020**; 23(12): 1552-60.
120. Turner HC, Toor J, Hollingsworth TD, Anderson RM. Economic Evaluations of Mass Drug Administration: The Importance of Economies of Scale and Scope. Clin Infect Dis **2018**; 66(8): 1298-303.
121. Shimakawa Y, Lemoine M, Bottomley C, Njai HF, Ndow G, Jatta A, Tamba S, Bojang L, Taal M, Nyan O, D'Alessandro U, Njie R, Thursz M, Hall AJ. Birth order and risk of hepatocellular carcinoma in chronic carriers of hepatitis B virus: a case-control study in The Gambia. Liver Int **2015**; 35(10): 2318-26.
